# Comparative anatomical and transcriptomic insights into *Vaccinium corymbosum* flower bud and fruit throughout development

**DOI:** 10.1186/s12870-021-03067-6

**Published:** 2021-06-24

**Authors:** Li Yang, Liangmiao Liu, Zhuoyi Wang, Yu Zong, Lei Yu, Yongqaing Li, Fanglei Liao, Manman Chen, Kailing Cai, Weidong Guo

**Affiliations:** 1 College of Chemistry and Life Sciences, Zhejiang Normal University, Jinhua, Zhejiang 321004 P. R. China; 2 Zhejiang Provincial Key Laboratory of Biotechnology on Specialty Economic Plants, Zhejiang Normal University, Jinhua, Zhejiang 321004 P. R. China; 3 Zhejiang College of Security Technology, Wenzhou, Zhejiang 325000 P. R. China

**Keywords:** Cell proliferation, Cell expansion, Fruit development, Comparative transcriptome, *TIFY* genes, Blueberry

## Abstract

**Background:**

Blueberry (*Vaccinium* spp.) is characterized by the production of berries that are smaller than most common fruits, and the underlying mechanisms of fruit size in blueberry remain elusive. *V. corymbosum* ‘O’Neal’ and ‘Bluerain’ are commercial southern highbush blueberry cultivars with large- and small-size fruits, respectively, which mature ‘O’Neal’ fruits are 1 ~ 2-fold heavier than those of ‘Bluerain’. In this study, the ontogenetical patterns of ‘O’Neal’ and ‘Bluerain’ hypanthia and fruits were compared, and comparative transcriptomic analysis was performed during early fruit development.

**Results:**

*V. corymbosum* ‘O’Neal’ and ‘Bluerain’ hypanthia and fruits exhibited intricate temporal and spatial cell proliferation and expansion patterns. Cell division before anthesis and cell expansion after fertilization were the major restricting factors, and outer mesocarp was the key tissue affecting fruit size variation among blueberry genotypes. Comparative transcriptomic and annotation analysis of differentially expressed genes revealed that the plant hormone signal transduction pathway was enriched, and that jasmonate-related *TIFYs* genes might be the key components orchestrating other phytohormones and influencing fruit size during early blueberry fruit development.

**Conclusions:**

These results provided detailed ontogenetic evidence for determining blueberry fruit size, and revealed the important roles of phytohormone signal transductions involving in early fruit development. The *TIFY* genes could be useful as markers for large-size fruit selection in the current breeding programs of blueberry.

**Supplementary Information:**

The online version contains supplementary material available at 10.1186/s12870-021-03067-6.

## Background

Fruits are a unique reproductive structure of angiosperms that promote seed dispersal, and provide abundant nutrients for human beings. Following long-term natural selection and artificial domestication, fruits have therefore taken on diverse forms and characteristics, including being fleshy or dry, indehiscent or dehiscent, and having apocarpous or syncarpous carpels [1, 2]. Among these adaptive characteristics, fruit size/weight is one vital agronomic trait for plant evolution and crop improvement, and is essential for yield, quality and consumer acceptance. A typical example is the tomato fruit, one of the successfully domesticated crops, which was increased more than 100-fold in weight from only a few grams to approximately 1 kg [2, 3]. Studies have identified several critical regulators of fruit size/weight, including climate, management, genotype, nutrition, fruit load and interactions of external and internal factors [4]. Moreover, final fruit size/weight is determined by cell proliferation and expansion, which is involved in successive processes of floral meristem, gynoecium formation, pollination and fertilization, locule and seed formation, as well as fruit growth and development [2, 5–7]. From the cytological standpoint, cell proliferation activity is the building block for fruit composition, whereas cell volume determines its final size [4, 8].

At present, important advances have been made in the model plants *Arabidopsis thaliana*, *Solanum lycopersicum* and *Oryza sativa*, and multiple genes, regulators and signalling pathways affecting fruit size/weight have been identified, such as *Fruit weight 2.2* (*FW2.2/CNR*), *Fruit weight 3.2* (*FW3.2/KLUH*), *Fruit weight 11.3* (*FW11.3/CSR*), *ENO* (*Excessive Number of Floral Organs*), etc. [2, 3, 6, 9–16]. Most of fruit size/weight regulators have ancient origins and relatively conserved functions among species with close genetic relationships, significant differences in expression patterns or biological functions were also identified in different species or cultivars, nevertheless. For instance, *FW2.2* (*fruit weight 2.2*), the first isolated quantitative trait locus from *S. lycopersicum*, contributes to approximately 30% of fruit weight variation by negatively regulating cell division [2, 12]; however, the highest difference in *FW2.2* transcript abundance between *Vaccinium corymbosum* cultivars with large and small-size fruits was not occurred during flower bud enlargement and early fruit development (the fastest stages for cell proliferation), indicating that complex mechanisms are involved in blueberry fruit development and fruit size/weight variation [17]. Therefore, identifying more regulators and their biological functions will help us to further interpret the fundamental mechanisms of fruit size/weight as well as develop practical applications to control these traits.

Blueberry (*Vaccinium* spp.), one of major genera in the tribe Vacciniae of the Ericaceae, produces a small false berry with high levels of bioactive metabolites, that have been discovered to be beneficial to human health, with antioxidant, anti-inflammatory, anticarcinogenic, antiobesity properties, and neuroprotective activities [18, 19]. However, blueberry fruit is smaller than most domesticated fruit, such as tomato, kiwifruit, and grape, whereas the systematic anatomical and molecular regulatory mechanisms of fruit size/weight remained elusive. In this work, temporal and spatial patterns of cell proliferation and expansion in the *V. corymbosum* cultivars ‘O’Neal’ and ‘Bluerain’, which represent large and small fruit, respectively, during hypanthia (receptacle and inferior tissues of flower bud, and form fruit after pollination and fertilization) and fruit development were counted and compared. In addition, comparative transcriptomic analysis was performed in these cultivars during anthesis and early fruit development. These results provide detailed ontogenetic evidence for the spatiotemporal patterns of cell proliferation and expansion and help to identify potential genes involved in regulating blueberry fruit size/weight.

## Results

### Description of *V. corymbosum* flower bud and fruit development

**Fig. 1:**
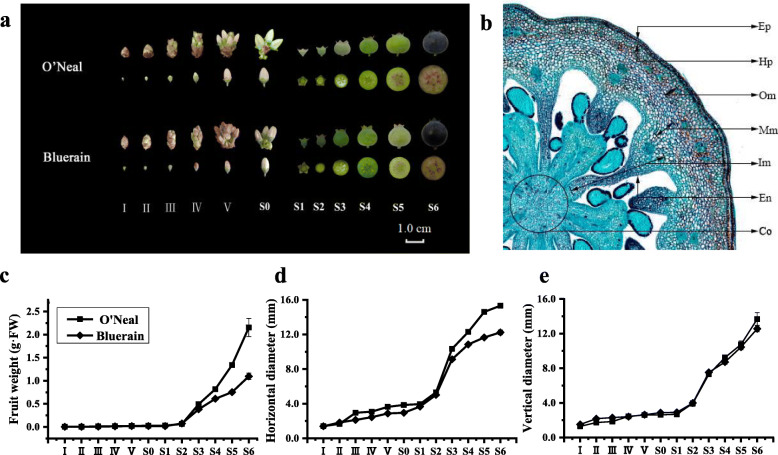
Developmental stages of *V. corymbosum* ‘O’Neal’ and ‘Bluerain’ flower buds and fruits. **a** Developmental stages of ‘O’Neal’ and ‘Bluerain’ flower buds and fruits. **b** Microscopic structures of ‘O’Neal’ fruit (stage S0) analyzed by equatorial paraffin section. **c**, **d**, **e** Quantification of fruit weight, horizontal and vertical diameters at different developmental stages. Co: columella; Ep: epidermis; Hp: hypodermis; Om: outer mesocarp; Mm: middle mesocarp; Im: inner mesocarp; En: endocarp

Following our previous study [20], the developmental process of ‘O’Neal’ and ‘Bluerain’ flower buds and fruits were divided into 12 stages (Fig. 1). At stage I, plump buds were enclosed by brown bracts, and the widest horizontal diameters of ‘O’Neal’ and ‘Bluerain’ hypanthia were approximately 1.40 mm. Individual flowers expanded beyond the bracts from stage IV, while the equatorially horizontal diameters of hypanthia from ‘O’Neal’ and ‘Bluerain’ were approximately 3.08 and 2.44 mm, respectively. The flower at the early-bloom stage was defined as the stage S0 or anthesis. After pollination and fertilization (stages S0-S1), fruit exhibited a double-sigmoidal growth pattern, which was categorized into three phases [21, 22], an early and first rapid growth phase (stages S2-S3, approximately 12 d), a long, slow-growing period (stages S3-S4, 24 ~ 30 d), and a second rapid growth phase (stages S5-S6, approximately 6 d). From stage S5, anthocyanin initially accumulated in the exocarp, and fruit gradually became dark purple or blue, maturing at stage S6. In total, the fruit growth period of ‘O’Neal’ and ‘Bluerain’ lasted approximately 74 d from anthesis.

### Time-course analysis of cell proliferation and expansion patterns throughout *V. corymbosum* hypanthia/fruit development

**Fig. 2:**
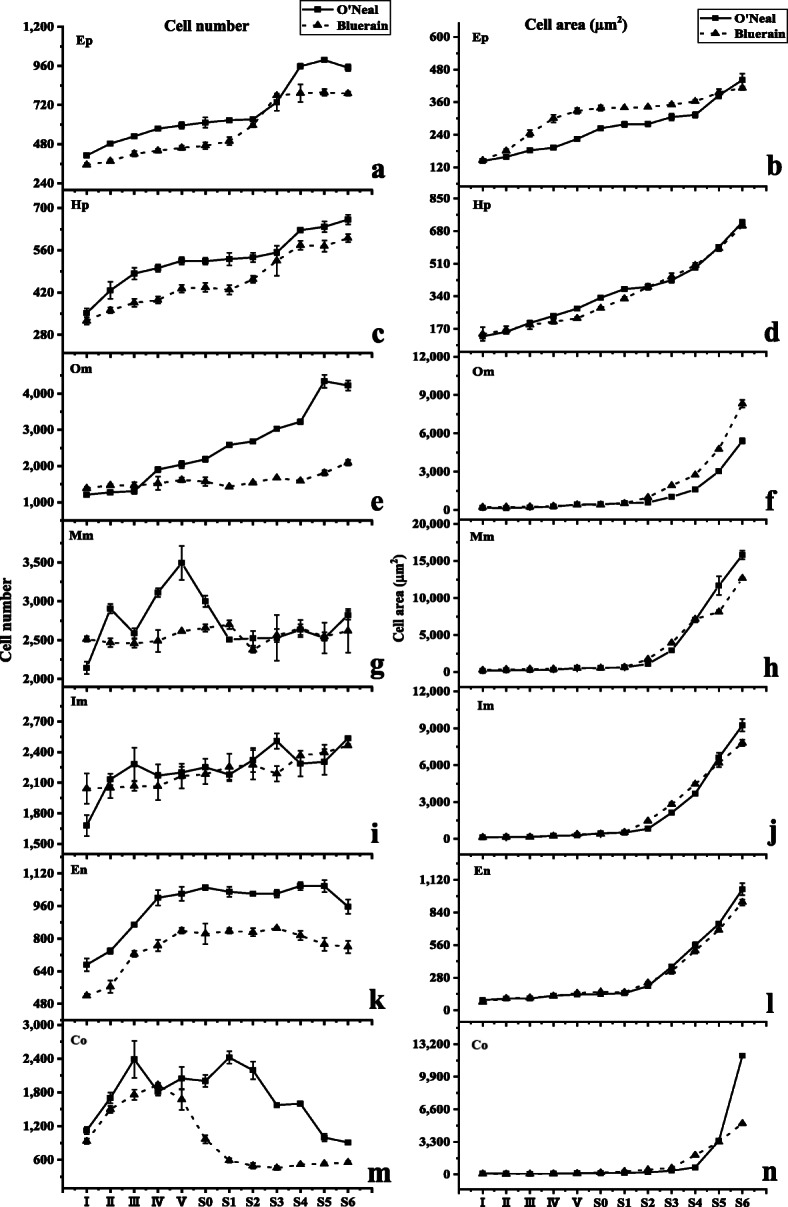
Spatiotemporal change of cell number and cell area throughout *V. corymbosum* ‘O’Neal’ and ‘Bluerain’ hypanthia/fruit development. Co: columella; Ep: epidermis; Hp: hypodermis; Om: outer mesocarp; Mm: middle mesocarp; Im: inner mesocarp; En: endocarp

Referring to the delimitation standards of Cano-Medrano and Darnell [23] and Renaudin et al. [24], equatorial hypanthia and fruit of blueberry were manually divided into 7 parts (Fig. 1b), including a single cell layer of the epidermis (Ep), hypodermis (Hp) and endocarp (En) and multiple cell layers of outer mesocarp (Om), middle mesocarp (Mm), inner mesocarp (Im) and columella (Co). The mean cell number and cell area in each representative tissue were measured, calculated and analyzed as follows (Fig. 2).

The cell shapes, along with cell proliferation and expansion patterns in the epidermis and hypodermis throughout ‘O’Neal’ and ‘Bluerain’ hypanthia and fruit development were similar (Fig. 2a-d). At stage I, epidermal and hypodermal cells were rectangular, and the anticlinal walls were longer than the periclinal walls. Thereafter epidermal and hypodermal cells gradually became square (stage IV) and rectangular again (stage S0, Fig. S1). The cell numbers of ‘O’Neal’ epidermis and hypodermis at stage I were slightly greater than those of ‘Bluerain’ (Fig. 2a and c), although the horizontal diameters of hypanthia were similar (Fig. 1d), whereafter, rapid cell proliferation occurred at stages I-III. After a long period of growth arrest, epidermal and hypodermal cells showed a second rapid cell proliferation again from stages S1/S2, and then remained constant at stages S4/S5. Notably, ‘Bluerain’ epidermis cells were larger than ‘O’Neal’ cells throughout development, except in stages I and S6, while the area of ‘O’Neal’ and ‘Bluerain’ hypodermal cells were approximately similar (Fig. 2b and d).

Figure 2e showed that the cell proliferation patterns of ‘O’Neal’ and ‘Bluerain’ outer mesocarp were dramatically different. Throughout development, the cell number of ‘O’Neal’ outer mesocarp was nearly increased approximately 3, 000 (~ 1000 cells before anthesis, and ~ 2, 000 cells after anthesis), but approximately 700 in ‘Bluerain’ (~ 200 cells before anthesis, and ~ 500 cells after anthesis). The cell area of both cultivars displayed a slight increase before stage S0, and a rapid expansion from stage S1 until fruit maturity. The mean cell area of outer mesocarp was increased ~ 30.6-fold in ‘O’Neal’ throughout development, and ~ 36.6-fold in ‘Bluerain’ (Fig. 2f). In addition, the total outer mesocarp area at stage S6 was approximately 28.61mm^2^ in ‘O’Neal’ and 18.12 mm^2^ in ‘Bluerain’, which was 12.0 and 9.3% of the area of the mature fruit, respectively (Figs. S2 and S3).

The initial cell numbers of middle and inner mesocarp were higher than those of other hypanthia/fruit tissues; however, the cell numbers of both cultivars maintained 2100 ~ 2500 throughout development (Fig. 2g and i). Similar to outer mesocarp, the middle and inner mesocarp cells also continuously expanded (Fig. 2h and j). In total, the mean cell area of middle and inner mesocarp was increased 80.1 ~ 91.0-fold in ‘O’Neal’ and 48.6 ~ 56.4-fold in ‘Bluerain’, respectively (Fig. 2h). It was worth mentioning that total middle mesocarp area at stage S6 were approximately 35.0% (~ 85.40 mm^2^) in ‘O’Neal’ mature fruit and 24.4% (~ 47.51 mm^2^) in ‘Bluerain’ (Figs. S2 and S3). Similarly, the total inner mesocarp area at stage S6 was approximately 33.3% (~ 74.35 mm^2^) in ‘O’Neal’ and 14.5% (~ 28.21 mm^2^) in ‘Bluerain’.

The shapes, and the increasing patterns of cell numbers and area of ‘O’Neal’ and ‘Bluerain’ endocarp throughout development basically followed those of epidermal cells (Fig. 2k and l), but endocarp cell numbers in ‘O’Neal’ were significantly higher than those in ‘Bluerain’ throughout development. The mean endocarp cell area of the two cultivars increased 11 ~ 12-fold throughout flower bud and fruit development, but increased ~ 6.5-fold in ‘O’Neal’ and ~ 4.9-fold in ‘Bluerain during fruit development.

Compared with other hypanthium/fruit tissues, columella displayed a distinctive cell proliferation pattern (Fig. 2m). The cell number of ‘O’Neal’ columella increased rapidly at the early stages of flower bud enlargement (stages I ~ III), and thereafter decreased dramatically from stage S1, whereas the cell number of ‘Bluerain’ increased rapidly at stages I ~ IV, decreased from stages IV to S1, and then remained constantly after stage S2. Meanwhile, columella cells expanded slightly before anthesis, and then dramatically increased afterwards (Fig. 2n). Throughout development, the mean columella cell area was increased ~ 122-fold in ‘O’Neal’, and ~ 76.5-fold in ‘Bluerain’.

### Developmental stage-based RNA-seq profiles of *V. corymbosum* during early fruit development

**Fig. 3:**
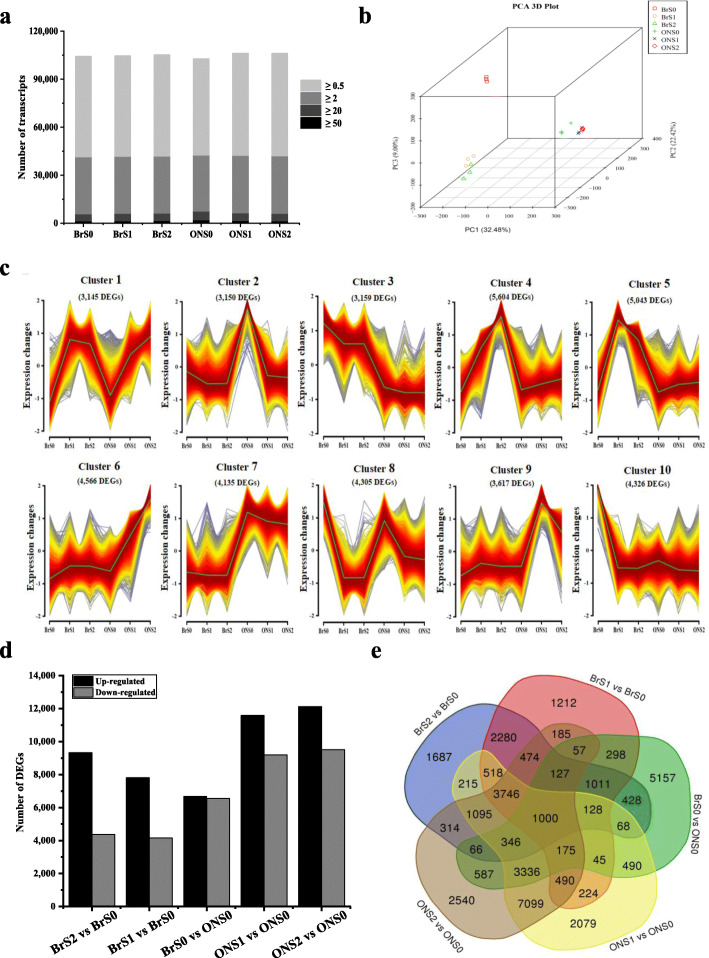
Comparative transcriptomic analysis of *V. corymbosum* ‘O’Neal’ and ‘Bluerain’ hypanthia/fruit during early fruit development. **a** Numbers of detected expressed genes for each sample. **b** Principal component analysis (PCA) of 18 transcriptomic profiles. **c** Cluster analysis of DEG expression patterns. **d** Up- and down-regulated DEGs identified in the 5 comparisons. **e** Venn diagrams of DEGs

According to the cytological data described above, cell division was arrested at anthesis, and cell expansion was initiated dramatically from stage S1 or S2 (Figs. 2 and S3), indicating early fruit growth stages were key phases to affect blueberry fruit size/weight. To elucidate the possible molecular basis, along with cell proliferation and expansion variation during early fruit development of cultivars with different fruit size, RNA-seq analysis was conducted to generate transcriptomic profiles. Hypanthia/fruits at stages S0, S1 and S2 of ‘O’Neal’ and ‘Bluerain’ were sequenced and analyzed (Tables S1 and S2). After removing low-quality reads, adaptor sequences, and sequence contaminants, a total of 7.64 ~ 9.99 Gb of clean bases were obtained from each library. The Q30 of raw data, an indicator of high-quality reads, ranged from 92.40 to 93.33%. The total length, average length and N50 of assembled unigenes were 579,400,266 bp, 1069 bp and 1569 bp, respectively. Matched ratios of total and unique mapped reads onto the highbush blueberry ‘Draper’ genome were in the range of 87.79 ~ 91.30% and 57.19 ~ 60.06%, respectively. In addition, approximately 145,525 expressed genes were identified, including 110,765 annotated genes and 16,966 gene loci that were not been previously annotated in the reference ‘Draper’ transcriptome (Fig. 3a).

PCA revealed a clear clustering of transcriptomic profiles, corresponding to cultivars and developmental stages (Fig. 3b). Eighteen libraries were mainly classified into two groups by cultivar, while samples at early fruit development (stages S1 and S2) were clustered together, suggesting that a program of fruit developmental differentiation was initiated between stages S0 and S1. To validate the reliability of transcriptomic profiles, the relative expression levels of 15 randomly selected genes were verified by qPCR (Fig. S4). The results showed that the expression abundance of selected genes was closely correlated with the RNA-seq data, indicating that the transcriptomic data were reliable. Gene expression clustering was also performed to identify the differentially expressed genes (DEGs) with similar expression patterns. Through this analysis, 10 clusters and approximately 41,000 DEGs exhibited dynamic expression changes during ‘O’Neal’ and ‘Bluerain’ early fruit development (Fig. 3c), and the huge number of clusters and diverse expression patterns indicated that fruit growth and development at early stages was a complex biological process.

### Functional classification and enrichment analysis of DEGs during *V. corymbosum* early fruit development

The expression, indicated by FPKM values, exhibited correlations (Spearman correlation coefficient = 0.73 ~ 0.84) among three biological replicates (Fig. S5) and was used to screen the transcriptional differences. Approximately 40% of expressed genes in each transcriptomic group had FPKM values lower than 0.5, and more than 6.5% of expressed genes had FPKM values more than 20 (Fig. 3a and Table S2). In this study, the specifically expressed genes with a fold change expression ratio of mean FPKM ≥3 (log_2_FoldChange ≥ 1.58) and Padj. < 0.01 in each comparison were chosen for further study. With this standard, a total of 11,970, 13,503, 13,219, 21,054 and 21,637 DEGs were identified from transcripts of ‘Bluerain’ at stages S1 and S2 compared with BrS0 expressed genes, along with transcripts of BrS0, ONS1 and ONS2 compared with ONS0 expressed genes. In addition, the numbers of up-regulated DEGs were obviously higher than down-regulated DEGs, except the numbers were similar in the comparisons betweem BrS0 and ONS0 (Fig. 3d and e).

The DEGs were then screened by KEGG enrichment analysis to identify the related functional and metabolic pathways and were assigned to approximate 360 predicted pathways. In the top 10 pathways, the annotated DEGs shared among 5 comparisons (Table S3 and Fig. S6) were highly enriched in the pathways of “biosynthesis of secondary metabolites (ko01110)”, “glycolysis/gluconeogenesis (ko00010)” and “plant hormone signal transduction (ko04075)”. In addition, numerous DEGs were enriched in the GO classification terms “response to hormone (GO: 0009725)”, “response to abscisic acid (ABA, GO: 0009737)”, “response to cytokinin (CTK, GO: 0009735)”, and “regulation of jasmonate mediated signaling pathway” (GO: 2000022)” (Fig. S7).

### DEGs associated with the plant hormone signal transduction pathway

It was generally known that phytohormones at imperceptibly low concentration play crucial roles in coordinating almost all aspects of plant growth and development. In this study, approximately 390 up-regulated DEGs and 167 down-regulated DEGs affiliated with the plant hormone signal transduction (ko04075) pathway were filtered, including 21 up-regulated and 1 down-regulated DEGs that were expressed across both cultivars and various developmental stages (Tables S4, S5). The most representative hormone signal transduction-related DEGs were associated with auxin (103 transcripts), followed by salicylic acid (SA), ABA, CTK, ethylene (ETH), brassinosteroid (BA), jasmonate (JA) and gibberellin (GA).

Although the DEG numbers involved in the auxin signal transduction pathway were highest, their FPKM values were relatively low (Table S5). The expression levels of most of auxin-related DEGs, as well as genes encoding auxin-responsive IAA (Aux/IAA) and indole-3-acetic-amido synthetase GH3-like genes in ‘Bluerain’ at stages S0 and S1 were higher than those of ‘O’Neal’, indicating that fewer auxin signals in ‘Bluerain’ hypanthia and fruit were delivered, thus limited cell proliferation and seed development. The expression levels of most GA-, ABA-, ETH-related signal transduction genes in the ‘Bluerain’ hypanthia and fruits were also higher than those in ‘O’Neal’, especially at stage S2, indicating that these phytohormone signals might affect fruit growth through cell expansion, not cell division.

**Fig. 4:**
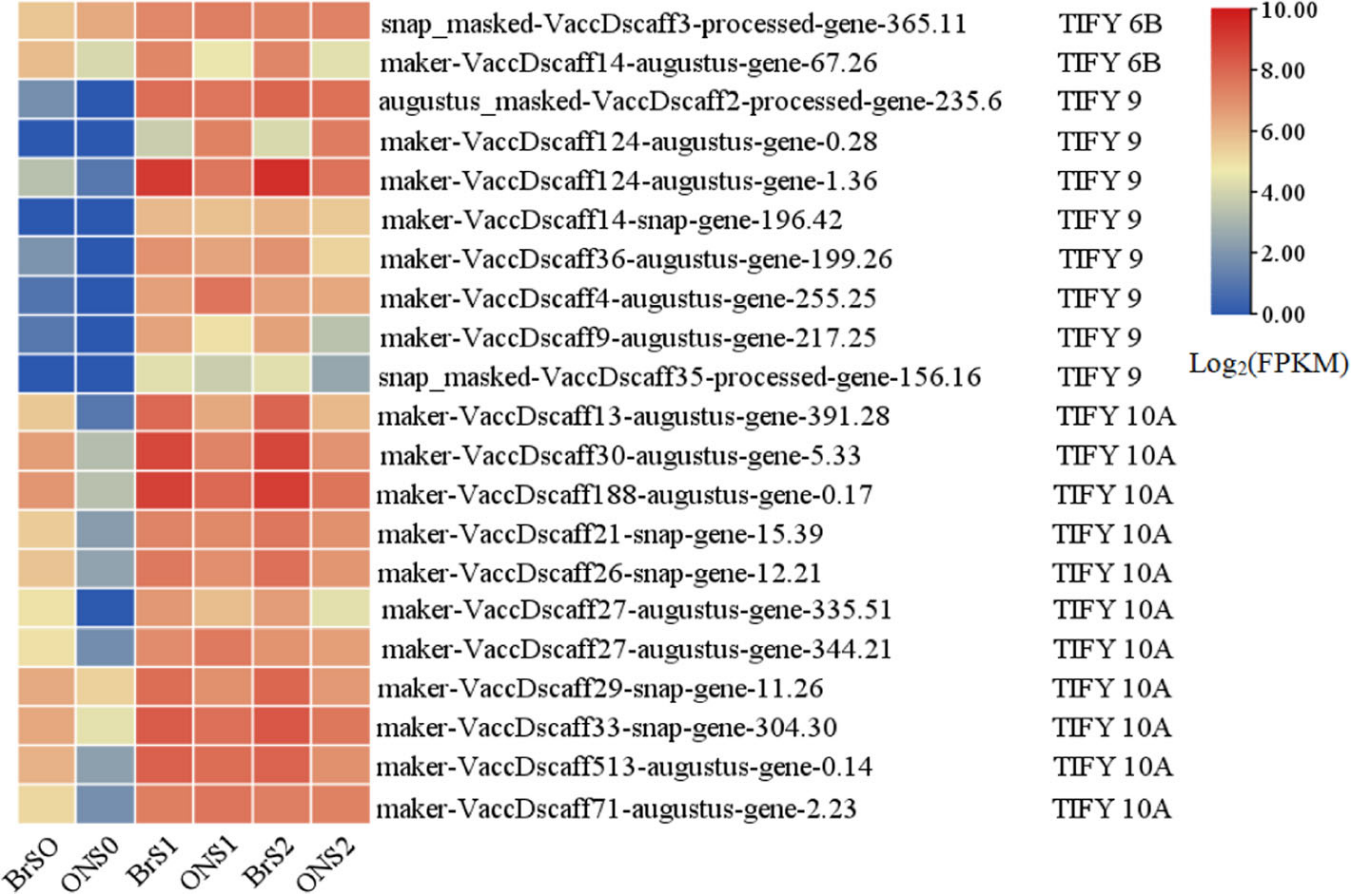
Expression profile of *TIFYs* of *V. corymbosum* ‘O’Neal’ and ‘Bluerain’ hypanthia/fruit during early fruit development. Each stage of early fruit development in ‘O’Neal’ and ‘Bluerain’ was listed horizontally. The color represented the expression level (Log_2_FPKM) of DEGs, and the FPKM value was the fragments per kilobase of transcript per million fragments

It was interesting that the expression levels of most DEGs responding to JA signaling, especially those encoding TIFY (or jasmonate ZIM-domain protein, JAZ) subfamily members *TIFY9* and *TIFY10A*, were obviously higher than those related to other phytohormones, particularly in ‘Bluerain’ fruit at stages S1 and S2 (Fig. 4 and Table S5), suggesting that JA might be the key phytohormone influencing size during early fruit development and might play central roles in orchestrating other phytohormones to improve fruit growth and development.

## Discussion

The false berry of *V. corymbosum* mainly originates from an inferior ovary, sepals and hypanthium [25]. Mesocarp and columella tissues are the primary edible portions (Fig. 1b), similar to model crop *S. lycopersicum*. In the recent past, researches on blueberry fruit have largely focused on ripening process, especially with regard to fruit quality and storage [25–30]. However, much less is explored about the cytological variation throughout flower bud and fruit growth among cultivated genotypes, along with its molecular regulatory mechanisms during early fruit development. In the case of this study, a global spatiotemporal analysis of cell proliferation and expansion during *V. corymbosum* ‘O’Neal’ and ‘Bluerain’ flower bud and fruit development and comparative transcriptomic analysis during anthesis and early fruit development were carried out. These results illustrated the specific spatiotemporal cell proliferation and expansion patterns associated with blueberry fruit ontogeny, and multiple plant hormone signal transduction pathways were found to be involved in early blueberry fruit development, possibly influencing fruit size/weight among cultivars.

### *V. corymbosum* hypanthia/fruit exhibited intricate tissue- or cell layer-specific cell proliferation and expansion patterns throughout development

**Fig. 5:**
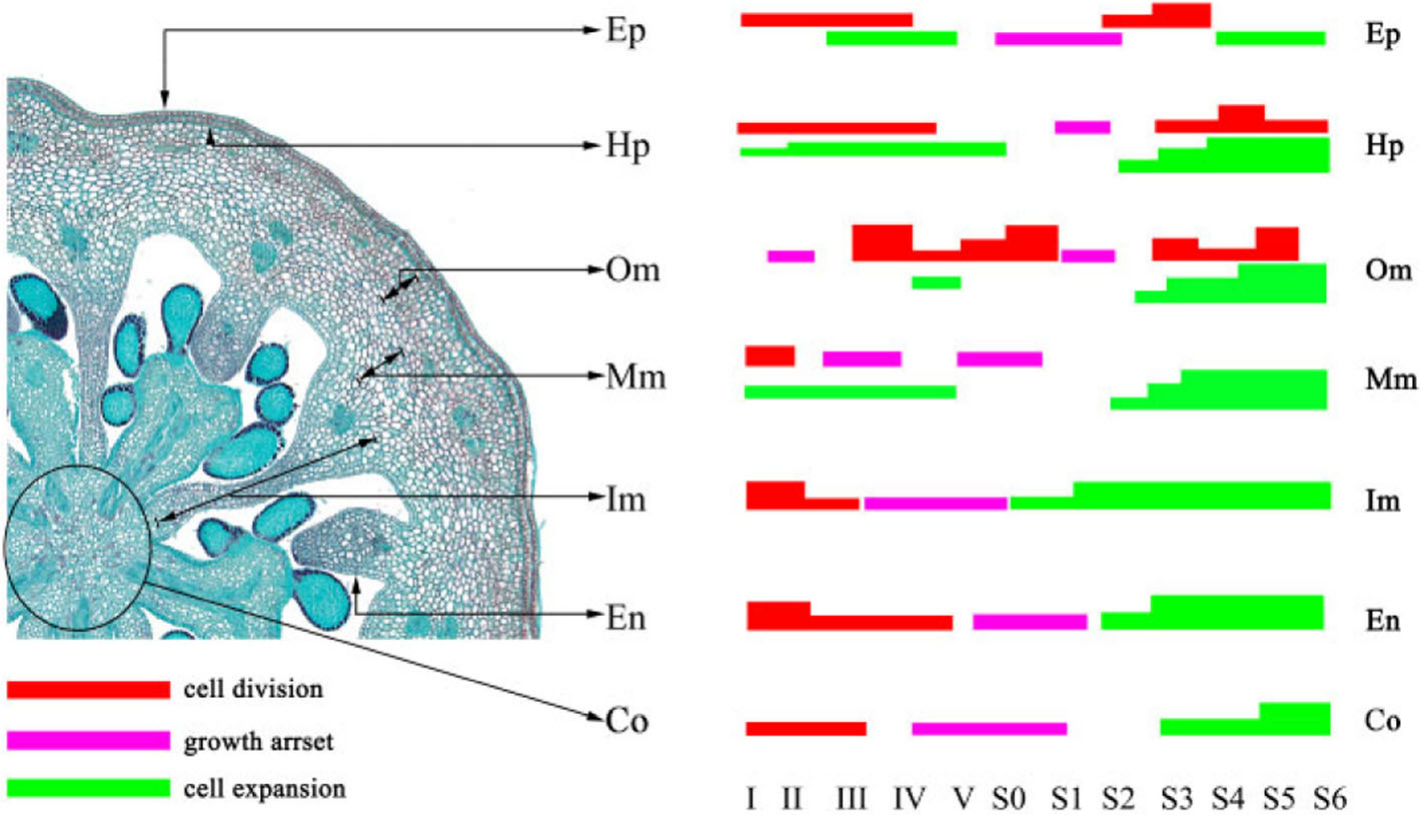
Spatiotemporal growth and developmental models of *V. corymbosum* hypanthia/fruit. Co: columella; Ep: epidermis; Hp: hypodermis; Om: outer mesocarp; Mm: middle mesocarp; Im: inner mesocarp; En: endocarp

In previous studies, fruit initiated cell division immediately after pollination and fertilization, and then experienced a long cell expansion period until maturity with a long/short overlap among varieties [4, 24, 31]. However, the cell proliferation and expansion of ‘O’Neal’ and ‘Bluerain’ hypanthia/fruits did not follow the ‘routine’ trend, and exhibited intricate spatiotemporal developmental patterns (Fig. 5). For instance, the cell numbers of middle and inner mesocarps remained constant, and columella cell numbers increased dramatically before anthesis, and then gradually decreased during fruit development, whereas its cell volume markedly increased after fertilization. These results indicated that programmed developmental differentiation, including cell proliferation and expansion, was involved in *V. corymbosum* pericarp growth (Fig. 2). Zhang et al. had reported that most spatiotemporally expressed genes, correlated with cell division and embryo formation, were involved in regulating early tomato fruit development [32]. Moreover, the development and ripening of tomato fruit are regulated by the timing and distribution of gene regulatory and structural networks, and exhibit spatial and developmental gradients [33]. These studies demonstrated that fruit growth was precisely modulated by complex temporal and spatial regulatory mechanisms and networks among species.

### Cell number before anthesis was a fundamental factor influencing *V. corymbosum* fruit size

16 ~ 18 d after anthesis, presumably in response to pollination and fertilization, the hypanthia/young fruit of ‘O’Neal’ and ‘Bluerain’ exhibited a dramatic morphological change. In general, cell division and expansion were initiated in an orderly manner after fertilization, while the final cell number and volume in the mature fruit were determined by the cell number and volume at anthesis, and the rate and duration of cell division and cell expansion thereafter [34, 35]. Following the increasing tendency of cell layers [22], the mean cell number present in hypanthia at stage I was approximately 59.7% (‘O’Neal’) and 82.8% (‘Bluerain’) of the final fruit cell number, even as these ratios reached 91.2% (‘O’Neal’) and 90.9% (‘Bluerain’) at anthesis, respectively, indicating that not only the initiated cell number, but also the cell proliferation ability in hypanthia before anthesis were vital factors determining final fruit size. Similar cell proliferation trends have been reported in apple, grape, *Rubus*, certain *Ribes* [35] and kiwifruit [36], and factors that affect cell proliferation during flower bud enlargement might have especially crucial impacts on fruit size [23]. Although Johnson et al. [37] demonstrated that cell number primarily facilitated variation in fruit size among 20 *V. ashei* genotypes, cell number in the mature fruit was not significantly positively correlated with cell number at bloom, indicating that the magnitude and activity of cell proliferation before anthesis were superordinate factors determining variation in final cell number and fruit size among blueberry genotypes.

### Cell expansion after fertilization also played important role in determining *V. corymbosum* fruit size

Although cell number was conformed as an important factor contributing to fruit size variation, some researchers suggested that cell expansion produced a greater effect on the size of fleshy fruits [1, 23, 35]. In this study, the cell areas of outer, middle and inner mesocarps, and columella expanded logarithmically from stage S1 until maturity, while the final cell areas of the epidermis, hypodermis and endocarp were relatively small (Fig. 2). In *V. ashei* ‘Beckyblue’ fruits, cell expansion was the primary factor for final fruit size, which agrees with the results for grape and cucumber [23]. However, for a wide range of fruit species, such as apple, strawberry, peach, apricot, pear and loquat, the cell number produced in the period immediately after pollination was the main factor related to final fruit size [7, 23, 31, 38], indicating that more complex regulatory mechanisms were involved in fruit growth and size after fertilization.

### Outer mesocarp was the key tissue determining fruit size variation among *V. corymbosum* genotypes

The mesocarp cell number in *V. ashei* fruit accounted for up to 75% of the total pericarp cell number, and the cell sizes of the middle and inner mesocarps of ripe pollinated fruits were dramatically larger than those of ripe GA_3_-induced parthenocapic fruits, indicating that more phytohormones, other than GAs, were induced or produced by seeds [23]. In this study, mesocarp tissues (including outer, middle and inner mesocarps) of ‘O’Neal’ and ‘Bluerain’ at stage S0 accounted for approximately 80.3 and 46.2% of mature fruit tissues, respectively. The cell numbers of the middle mesocarp and inner mesocarp were similar, but the final cell number of the ‘O’Neal’ outer mesocarp was 1.0-fold higher than that of ‘Bluerain’, suggesting outer mesocarp was the key tissue determining fruit size variation among genotypes. In fact, the seed numbers of mature ‘O’Neal’ fruit was approximately 1.7-fold greater than those of ‘Bluerain’ fruit [17], indicating that phytohormones, which might be induced or produced by seeds, were therefore likely to promote fruit size increase, especially for mesocarp tissues [39].

### JA might be the key phytohormone that orchestrates and balances other phytohormones and influences *V. corymbosum* fruit size during early development

Phytohormones are organic signaling molecules that coordinate cellular activities, pattern formation, vegetative and reproductive development, and biotic and abiotic stress responses. In fact, fruit growth is actually controlled by a complex hormonal regulatory network, and the importance of phytohormones for flower and fruit development has been well elucidated in the model plants *Arabidopsis* and tomato [40–42]. However, the concrete biological functions of various phytohormones during blueberry flower bud enlargement and fruit development are scarcely reported. In this study, comparative transcriptomic analysis was performed to profile three early developmental stages of ‘Bluerain’ and ‘O’Neal’ fruit, and the expression levels of most genes involved in the phytohormone signal transduction pathway were obviously different. Many of these genes, proteins and transcription factors associated with phytohormone signal transduction in the young ‘Bluerain’ fruit showed higher expression, except auxin-related genes at stages S1 and S2, CTK-related genes at stages S0 and S1, GA-, ABA- and ETH-related genes at anthesis, indicating that auxin and CTK signals promoted more cell proliferation in the ‘O’Neal’ young fruit, whereas ripening-related signals (ABA and ETH) not only arrested cell division at anthesis, but also restricted cell proliferation in the young ‘Bluerain’ fruit.

**Fig. 6:**
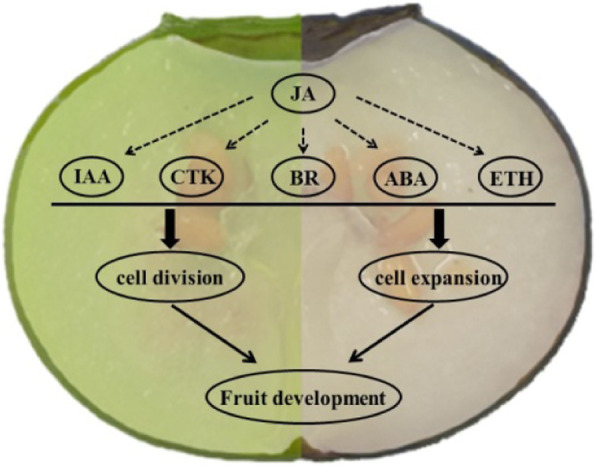
JA might be a master regulator for *V. corymbosum* fruit size/weight during early development

JA is one of primary defense compounds, and controls cell cycle, root extension, leaf growth and senescence, stomatal closure, mutualistic interactions, secondary metabolism (especially anthocyanin accumulation), etc. [43–47]. For instance, expression of *Arabidopsis DEFECTIVE IN ANTHER DEHISCENCE* 1 (*DAD1*, encodes the initial enzyme for JA biosynthesis) gene in the stamen filaments promoted water transport by the synchronization of anther dehiscence, pollen maturation and flower opening [48], and silence of *DAD1* decreased fruit and seed size through altering *Jatropha curcas* flower and fruit development together with lower endogenous JA and JA-Ile levels [49]. In addition, *S. lycopersicum* JA insensitive 1–1 (*jai*, also called *coi1* in *A. thaliana*) mutant delayed senescence of petals, styles and glabrous ovaries [43]. In the present case, JA signal-related genes were somewhat predominantly expressed, particularly in the young fertilized ‘Bluerain’ fruit, suggesting that JA (mainly coordinated by *TIFY9* and *TIFY10A*) might orchestrate cell proliferation and expansion in fruit, in association with other phytohormones, and influence fruit size during early development (Fig. 6).

In fact, the FPKM values of blueberry *VcFW2.2*, *VcFW11.3*, *VcFW3.2* and *VcENO* orthologs (key regulatory genes for *S. lycopersicum* fruit weight/size variation) were checked, but its expression levels were relative low, even undetectable (Table S5), indicating that these genes were not key regulatory genes for blueberry fruit weight/size, or the tissues sampled were not the similar timepoint.

## Conclusions

Based on the ontogenetical and comparative transcriptomic evidence, southern highbush blueberry fruit exhibited intricate temporal and spatial cell proliferation and expansion patterns, and cell division before anthesis and cell expansion after fertilization were the major determinants, while outer mesocarp was the key tissue determining final fruit size/weight variation among genotypes. Moreover, multiple hormone signal transduction pathways were involved in early fruit development, and JA might be the key phytohormone that orchestrates and balances other phytohormones and influences blueberry fruit size. These data will facilitate more precise physical and molecular characterization of blueberry fruit developmental processes.

## Methods

### Plant materials

6 (or 8)-year-old *V. corymbosum* ‘O’Neal’ (bred in North Carolina in 1987) and ‘Bluerain’ (identified in Dalian University of Technology in 2010) plants under natural conditions were used in this study. According to Yang et al. [20], flower buds and fruits at different developmental stages (Fig. 1a) were randomly tagged, collected and processed. Specifically, flower buds from stages IV to S0 were stripped of corollas, stamens and pistils, and then used for further analysis. At least three biological replicates were used to minimize variation during processing.

### Paraffin section preparation, cell number and cell area measurement

The preparation and microscopic observation of histological sections were performed according to Yang et al. [20]. The equatorial cell number of a specific layer or tissue at different developmental stages was counted manually and by ImageJ software. The mean equatorial cell area was determined by dividing the delimited area by the cell number.

### RNA sequencing (RNA-seq) analysis

Transcriptomic sequencing and bioinformatic analysis was conducted on the hypanthia and young fruits of ‘O’Neal’ and ‘Bluerain’ during anthesis and early developmental period (Novogene Science and Technology Co., Ltd.; Origingene Biomedical Technology Co., Ltd.). In brief, total RNA was assessed by agarose gel electrophoresis, a NanoPhotometer spectrophotometer (Implecn, California, USA) and an Agilent Bioanalyzer 2100 system (Agilent Technologies, California, USA). Sequencing libraries were generated with purified mRNA by using the NEBNext Ultra™ RNA Library Prep Kit, and sequenced on an Illumina HiSeq-Xten platform in paired-end mode with a read length of 250 ~ 300 bp. Raw reads were first processed by removing reads containing adapters, reads containing > 10% poly-N, and low-quality reads (≤ 20 nucleotides).

Sequence assembly and annotation were accomplished using HISAT2 and StringTie softwares [50, 51] with the reference blueberry genome ‘Draper’ v1.0 (Genome Database of Vaccinium) [52]. Principal component analysis (PCA) was performed in the R package and plotted with the scatterplot3d library [53]. Adjusted *P*-values (Padj. < 0.01) of multiple tests were corrected using Benjamini and Yekutieli’s approach for controlling the false discovery rate (FDR) [54].

Fragments per Kb per million fragment (FPKM) values were calculated by script RSEM [55]. Differential expression analysis (DEGs, fold change ≥3 and Padj. < 0.01) across five pairwise comparisons of BrS1 vs BrS0, BrS2 vs BrS0, BrS0 vs ONS0, ONS1 vs ONS0 and, ONS2 vs ONS0 was performed with StringTie software, and annotated with the Kyoto Encyclopedia of Genes and Genomes (KEGG, www.kegg.jp/kegg/kegg1.html) and Gene Ontology (GO, http://geneontology.org/) databases. A Venn diagram of DEGs was generated with online software (http://bioinformatics.psb.ugent.be/webtools/Venn/). A heatmap was drawn by TBtools softeware (v1.05) [56], and the R package Mfuzz (version 2.34.0) was performed for cluster analysis of developmental expression patterns [57].

### Quantitative real-time PCR (qPCR) validation

Total RNA extraction and 1st-strand cDNA synthesis were performed according to our previous report [20]. Synthesized 1st-strand cDNAs were diluted 3-fold for qPCR validation. Specific primers (Table S6) of 15 randomly selected genes were designed by Primer-BLAST (https://www.ncbi.nlm.nih.gov/tools/primer-blast/), and *VcGAPDH* was used to normalize the amount of cDNA among samples [58]. qPCR reactions were performed on an ABI StepOne Plus™ RT-PCR system (Applied Biosystems Co., Ltd., Beijing, China). The PCR system, procedures and data analysis were performed as described in Liu et al. [59].

### Statistical analysis

Statistical analyses were performed using SPSS 17.0 software. The data were expressed as mean value and standard deviation (mean ± SD, *n* ≥ 3). Statistical significance was evaluated via independent sample t test (confidence interval = 95%) using SPSS17.0 software. Unless otherwise specified, figures were plotted by Origin Pro.8.6 software.

## Supplementary Information

**Additional file 1: Table S1.** Summary of transcriptomic data of *V. corymbosum* ‘O’Neal’ and ‘Bluerain’ during early fruit development. **Table S2.** FPKM distribution of expressed genes in the transcriptomic files during early *V. corymbosum* ‘O’Neal’ and ‘Bluerain’ fruit development. **Table S3.** KEGG function classification (Top 10 pathways) of DEGs for each comparison during early *V. corymbosum* ‘O’Neal’ and ‘Bluerain’ fruit development. **Table S4.** Specific DEGs involved in plant hormone signal transduction (ko04075) pathway for each comparison. **Table S5.** The expression levels and foldchanges of specific DEGs related to plant hormone signal transduction (ko04075) pathway. **Table S6.** Primers used in this study. **Figure S1.** Equatorial sections of *V. corymbosum* ‘O’Neal’ and ‘Bluerain’ hypanthia/fruits at different developmental stages. **Figure S2.** Total area of outer mesocarp, middle mesocarp and inner mesocarp during *V. corymbosum* ‘O’Neal’ and ‘Bluerain’ flower bud and fruit development. **Figure S3.** Total cell number and fruit area increase patterns during *V. corymbosum* ‘O’Neal’ and ‘Bluerain’ flower bud and fruit development. **Figure S4.** Relative expression levels of 15 randomly selected DEGs determined by qPCR. **Figure S5.** Spearman correlation coefficient of transcriptomic profiles of early *V. corymbosum* ‘O’Neal’ and ‘Bluerain’ fruit development. **Figure S6.** KEGG function classification of DEGs associated with early *V. corymbosum* ‘O’Neal’ and ‘Bluerain’ fruit development. **Figure S7.** GO function classification of the DEGs involved in biological processes during early *V. corymbosum* ‘O’Neal’ and ‘Bluerain’ fruit development.

## Data Availability

In this study, all materials were obtained from Zhejiang Normal University (Jinhua, Zhejiang Province, China), and the sample collection were complied with relevant institutional, national, and international guidelines and legislation. The raw transcriptomic data can be accessed from the NCBI Sequence Read Archive (SRA) platform (http://www.ncbi.nlm.nih.gov/sra/) under accession numbers SRX9831796 ~ SRX9831798 (BrS0), SRX9847572 ~ SRX9847574 (BrS1), SRX9862519 ~ SRX9862521 (BrS2), SRX9847944 ~ SRX9847946 (ONS0), SRX9854591 ~ SRX9854593 (ONS1), and SRX9956371 ~ SRX9956373 (ONS2).

## Abbreviations

ABA: Abscisic acid
BR: Brassinosteroid
CTK: Cytokinin
Co: Columella
DEGs: Differential expression genes
En: Endocarp
Ep: Epidermis
ETH: Ethylene
FDR: False discovery rate
FPKM: Fragments per Kb per million fragments
GA: Gibberellin
GO: Gene Ontology
Hp: Hypodermis
Im: Inner mesocarp
JA: Jasmonate
KEGG: Kyoto Encyclopedia of Genes and Genomes Ortholog database
Mm: Middle mesocarp
Om: Outer mesocarp
PCA: Principal components analysis
qPCR: Quantitative real-time PCR
SA: Salicylic acid
TIFY: Jasmonate ZIM-domain protein
