# Supplementary material for: Comparative anatomical and transcriptomic insights into Vaccinium corymbosum flower bud and fruit throughout development
Source: BMC Plant Biol. 2021 Jun 24;21:289. doi: 10.1186/s12870-021-03067-6 (PMC8223347; doi:10.1186/s12870-021-03067-6)
Supplement: Supplementary file 1 — Additional file 1: Table S1. Summary of transcriptomic data of V. corymbosum ‘O’Neal’ and ‘Bluerain’ during early fruit development. Table S2. FPKM distribution of expressed genes in the transcriptomic files during early V. corymbosum ‘O’Neal’ and ‘Bluerain’ fruit development. Table S3. KEGG function classification (Top 10 pathways) of DEGs for each comparison during early V. corymbosum ‘O’Neal’ and ‘Bluerain’ fruit development. Table S4. Specific DEGs involved in plant hormone signal transduction (ko04075) pathway for each comparison. Table S5. The expression levels and foldchanges of specific DEGs related to plant hormone signal transduction (ko04075) pathway. Table S6. Primers used in this study. Figure S1. Equatorial sections of V. corymbosum ‘O’Neal’ and ‘Bluerain’ hypanthia/fruits at different developmental stages. Figure S2. Total area of outer mesocarp, middle mesocarp and inner mesocarp during V. corymbosum ‘O’Neal’ and ‘Bluerain’ flower bud and fruit development. Figure S3. Total cell number and fruit area increase patterns during V. corymbosum ‘O’Neal’ and ‘Bluerain’ flower bud and fruit development. Figure S4. Relative expression levels of 15 randomly selected DEGs determined by qPCR. Figure S5. Spearman correlation coefficient of transcriptomic profiles of early V. corymbosum ‘O’Neal’ and ‘Bluerain’ fruit development. Figure S6. KEGG function classification of DEGs associated with early V. corymbosum ‘O’Neal’ and ‘Bluerain’ fruit development. Figure S7. GO function classification of the DEGs involved in biological processes during early V. corymbosum ‘O’Neal’ and ‘Bluerain’ fruit development. [file 12870_2021_3067_MOESM1_ESM.doc]

**Comparative anatomical and transcriptomic insights into *Vaccinium corymbosum* flower bud and fruit throughout development**

Li Yang^1,2,†,*^, Liangmiao Liu^1^, Zhuoyi Wang^1^, Yu Zong^1,2^, Lei Yu^1^, Yongqaing Li^1,2^, Fanglei Liao^1,2^, Manman Chen^3^, Kailing Cai^1^, Weidong Guo^1,2^,*[[1]](#footnote-2)

S**upplementary files**

**Table S1.** Summary of transcriptomic data of *V. corymbosum* 'O'Neal' and 'Bluerain' during early fruit development.

**Table S2.** FPKM distribution of expressed genes in the transcriptomic files during early *V. corymbosum* 'O'Neal' and 'Bluerain' fruit development.

**Table S3.** KEGG function classification (Top 10 pathways) of DEGs for each comparison during early *V. corymbosum* 'O'Neal' and 'Bluerain' fruit development.

**Table S4.** Specific DEGs involved in plant hormone signal transduction (ko04075) pathway for each comparison.

**Table S5.** The expression levels and foldchanges of specific DEGs related to plant hormone signal transduction (ko04075) pathway.

**Table S6.** The expression levels and foldchanges of key homologous regulatory genes involved in *Solanum lycopersicum* fruit weight/size variation.

**Table S7.** Primers used in this study.

**Fig. S1.** Equatorial sections of *V. corymbosum* 'O'Neal' and 'Bluerain' hypanthia/fruits at different developmental stages.

**Fig. S2.** Total area of outer mesocarp, middle mesocarp and inner mesocarp during *V. corymbosum* 'O'Neal' and 'Bluerain' flower bud and fruit development.

**Fig. S3**. Total cell number and fruit area increase patterns during *V. corymbosum* 'O'Neal' and 'Bluerain' flower bud and fruit development.

**Fig. S4.** Relative expression levels of 15 randomly selected DEGs determined by qPCR.

**Fig. S5.** Spearman correlation coefficient of transcriptomic profiles of early *V. corymbosum* 'O'Neal' and 'Bluerain' fruit development.

**Fig. S6.** KEGG function classification of DEGs associated with early *V. corymbosum* 'O'Neal' and 'Bluerain' fruit development.

**Fig. S7.** GO function classification of the DEGs involved in biological processes during early *V. corymbosum* 'O'Neal' and 'Bluerain' fruit development.

**Table S1.** Summary of transcriptomic data of *V. corymbosum* 'O'Neal' and 'Bluerain' during early fruit development.

| Samples | Raw  Reads | Clean  Reads | Clean Bases (Gb) | Error rate  (%) | Q30  (%) | GC Content (%) | Reads aligned onto the 'Draper' genome | |
| --- | --- | --- | --- | --- | --- | --- | --- | --- |
|  |  |  |  |  |  |  | Total mapped | Uniquely mapped |
| ONS0_1 | 52,957,444 | 52,933,156 | 7.91 | 0.026 | 93.16 | 46.31 | 48,330,011 (91.30%) | 30,274,073 (57.19%) |
| ONS0_2 | 65,342,744 | 65,319,356 | 9.75 | 0.027 | 92.85 | 46.06 | 59,455,237 (91.02%) | 37,054,717 (56.73%) |
| ONS0_3 | 53,545,034 | 53,525,490 | 7.99 | 0.026 | 93.31 | 46.00 | 48,830,623 (91.23%) | 30,609,667 (57.19%) |
| ONS1_1 | 57,187,454 | 57,168,880 | 8.54 | 0.026 | 93.33 | 46.14 | 52,069,026 (91.08%) | 33,600,433 (58.77%) |
| ONS1_2 | 54,173,488 | 54,153,208 | 8.09 | 0.026 | 93.09 | 46.13 | 49,364,616 (91.18%) | 32,029,455 (59.15%) |
| ONS1_3 | 55,772,360 | 55,743,750 | 8.32 | 0.026 | 93.11 | 46.12 | 50,683,748 (90.92%) | 32,763,840 (58.78%) |
| ONS2_1 | 60,523,710 | 60,496,090 | 9.04 | 0.027 | 92.85 | 45.84 | 54,788,025 (90.57%) | 36,080,316 (59.64%) |
| ONS2_2 | 55,478,568 | 55,459,114 | 8.29 | 0.027 | 92.54 | 46.23 | 50,354,257 (90.80%) | 32,683,589 (58.93%) |
| ONS2_3 | 56,723,832 | 56,702,582 | 8.48 | 0.026 | 93.09 | 46.18 | 51,705,806 (91.19%) | 33,886,819 (59.76%) |
| BrS0_1 | 59,163,334 | 59,137,114 | 8.83 | 0.027 | 92.82 | 45.27 | 52,645,044 (89.02%) | 34,076,633 (57.62%) |
| BrS0_2 | 58,938,286 | 58,918,156 | 8.81 | 0.027 | 92.90 | 45.37 | 52,638,809 (89.34%) | 34,315,029 (58.24%) |
| BrS0_3 | 66,862,766 | 66,839,860 | 9.99 | 0.027 | 92.78 | 45.63 | 59,975,659 (89.73%) | 38,984,504 (58.32%) |
| BrS1_1 | 59,677,176 | 59,656,766 | 8.92 | 0.027 | 92.70 | 45.96 | 52,942,708 (88.75%) | 35,227,801 (59.05%) |
| BrS1_2 | 56,796,622 | 56,776,764 | 8.49 | 0.027 | 92.40 | 45.86 | 50,668,773 (89.24%) | 33,931,445 (59.76%) |
| BrS1_3 | 54,479,360 | 54,461,732 | 8.14 | 0.027 | 92.72 | 46.14 | 48,901,813 (89.79%) | 32,565,418 (59.80%) |
| BrS2_1 | 51,128,244 | 51,105,486 | 7.64 | 0.027 | 92.91 | 46.18 | 45708920 (89.44%) | 30,696,121 (60.06%) |
| BrS2_2 | 53,273,158 | 53,248,050 | 7.96 | 0.027 | 92.56 | 45.92 | 46,744,140 (87.79%) | 31,506,393 (59.17%) |
| BrS2_3 | 52,611,220 | 52,593,098 | 7.86 | 0.026 | 93.10 | 46.08 | 47,007,053 (89.38%) | 31,239,566 (59.40%) |

Notes: ONS0, ONS1 and ONS2 represented the samples at stages S0, S1 and S2 of 'O'Neal', respectively; and BrS0, BrS1 and BrS2 represented the samples at stages S0, S1 and S2 of 'Bluerain'. "_1", "_2" and "_3" represented the replications.

**Table S2.** FPKM distribution of expressed genes in the transcriptomic files during early *V. corymbosum* 'O'Neal' and 'Bluerain' fruit development.

| **Sample** | **0.5 ≤ FPKM** < **2** | **2 ≤ FPKM** < **20** | **20 ≤ FPKM** < **50** | **50 ≤ FPKM** |
| --- | --- | --- | --- | --- |
| ONS0 | 25,110 (41.72%) | 29,535 (49.07%) | 3,512 (5.84%) | 2,030 (3.37%) |
| ONS1 | 27,847 (43.62%) | 31,233 (48.92%) | 3,116 (4.88%) | 1,648 (2.58%) |
| ONS2 | 28,200 (43.95%) | 31,435 (48.99%) | 2,992 (4.66%) | 1,542 (2.40%) |
| BrS0 | 27,187 (43.27%) | 31,203 (49.66%) | 3,022 (4.81%) | 1,417 (2.26%) |
| BrS1 | 27,260 (43.33%) | 31,043 (49.34%) | 3,123 (4.96%) | 1,490 (2.37%) |
| BrS2 | 27,631 (43.63%) | 31,021 (48.98%) | 3,071 (4.85%) | 1,610 (2.54%) |

**Table S3.** KEGG function classification (Top 10 pathways) of DEGs for each comparison during early *V. corymbosum* 'O'Neal' and 'Bluerain' fruit development.

| **BrS0 vs ONS0**  Biosynthesis of amino acids  Biosynthesis of antibiotics  Biosynthesis of secondary metabolites  Glycolysis / Gluconeogenesis  Carbon metabolism  Microbial metabolism in diverse environments  Fatty acid degradation  **Plant hormone signal transduction**  Starch and sucrose metabolism  Metabolic pathways  **BrS1 vs BrS0**  Biosynthesis of secondary metabolites  Metabolic pathways  Photosynthesis  **Plant hormone signal transduction**  Phenylpropanoid biosynthesis  Flavonoid biosynthesis  Glycolysis / Gluconeogenesis  Carbon metabolism  Cutin, suberine and wax biosynthesis  Starch and sucrose metabolism | **ONS1 vs ONS0**  **Plant hormone signal transduction**  Biosynthesis of amino acids  Biosynthesis of secondary metabolites  Glycolysis/Gluconeogenesis  Carbon metabolism  Biosynthesis of antibiotics  Plant−pathogen interaction  Microbial metabolism in diverse environments  Influenza A  MAPK signaling pathway − plant |
| --- | --- |
| **BrS2 vs BrS0**  Biosynthesis of secondary metabolites  Metabolic pathways  Glycolysis / Gluconeogenesis  **Plant hormone signal transduction**  Photosynthesis  Carbon metabolism  Flavonoid biosynthesis  Phenylpropanoid biosynthesis  Starch and sucrose metabolism  Microbial metabolism in diverse environments | **ONS2 vs ONS0**  **Plant hormone signal transduction**  Biosynthesis of secondary metabolites  Glycolysis / Gluconeogenesis  Biosynthesis of amino acids  Amino sugar and nucleotide sugar metabolism  Glycine, serine and threonine metabolism  Plant−pathogen interaction  Influenza A  Microbial metabolism in diverse environments  MAPK signaling pathway − plant |

**Table S4. Specific DEGs involved in plant hormone signal transduction (ko04075) pathway for each comparison.**

| **Comparison** | **Number** | **DEGs** | **Functional annotation** |
| --- | --- | --- | --- |
| **Down** |  |  |  |
| BrS2 vs BrS0  BrS1 vs BrS0  BrS0 vs ONS0  ONS1 vs ONS0  ONS2 vs ONS0  BrS2 vs BrS0  BrS1 vs BrS0 | 1  13  10 | maker-VaccDscaff22-augustus-gene-238.25  maker-VaccDscaff38-augustus-gene-161.11 maker-VaccDscaff15-snap-gene-362.39 maker-VaccDscaff20-augustus-gene-43.48 maker-VaccDscaff23-snap-gene-355.33 maker-VaccDscaff9-snap-gene-226.23 maker-VaccDscaff46-snap-gene-106.24 maker-VaccDscaff12-snap-gene-56.27 augustus_masked-VaccDscaff20-processed-gene-43.7 maker-VaccDscaff33-augustus-gene-45.29 maker-VaccDscaff48-augustus-gene-71.46 maker-VaccDscaff1-augustus-gene-152.15 maker-VaccDscaff22-augustus-gene-355.20 maker-VaccDscaff31-snap-gene-168.18  maker-VaccDscaff21-augustus-gene-2.29 maker-VaccDscaff17-augustus-gene-265.28 maker-VaccDscaff21-augustus-gene-331.37 maker-VaccDscaff46-augustus-gene-177.34 maker-VaccDscaff19-augustus-gene-43.40 maker-VaccDscaff43-augustus-gene-31.37 maker-VaccDscaff47-snap-gene-169.37 maker-VaccDscaff31-snap-gene-332.46 maker-VaccDscaff7-snap-gene-5.36 maker-VaccDscaff47-augustus-gene-168.31 | Transcription factor TGA9  Auxin response factor 9  Indole-3-acetic acid-amido synthetase GH3.1  Auxin transporter-like protein 4  Auxin response factor 5  Histidine kinase 3  Transcription factor TGA9  Auxin response factor 5  Coronatine-insensitive protein 1  Serine/threonine-protein kinase CTR1  Auxin transporter-like protein 4  Serine/threonine-protein kinase BSK3  Indole-3-acetic acid-amido synthetase GH3.6  Two-component response regulator ORR22  Auxin-responsive protein IAA27  Two-component response regulator ARR5  Two-component response regulator ARR8  Auxin-induced protein AUX28  Auxin transporter protein 1  Auxin-induced protein AUX28  Auxin-induced protein 22D  Serine/threonine-protein kinase  Serine/threonine-protein kinase At4g35230  Auxin-induced protein AUX28 |
|  |  |  |  |
| BrS0 vs ONS0 | 22 | snap_masked-VaccDscaff2-processed-gene-394.25 maker-VaccDscaff8-snap-gene-111.37 maker-VaccDscaff2-augustus-gene-356.30 maker-VaccDscaff24-augustus-gene-350.41 maker-VaccDscaff5-snap-gene-323.24 maker-VaccDscaff17-snap-gene-122.34 maker-VaccDscaff8-augustus-gene-276.30 maker-VaccDscaff29-snap-gene-197.22 snap_masked-VaccDscaff24-processed-gene-76.15 maker-VaccDscaff22-augustus-gene-100.26 maker-VaccDscaff27-augustus-gene-84.18 maker-VaccDscaff1301-augustus-gene-0.5 maker-VaccDscaff44-augustus-gene-170.17 maker-VaccDscaff10-augustus-gene-90.15 maker-VaccDscaff24-augustus-gene-350.40 augustus_masked-VaccDscaff40-processed-gene-34.7 maker-VaccDscaff8-augustus-gene-370.27 maker-VaccDscaff3-snap-gene-17.37 augustus_masked-VaccDscaff23-processed-gene-307.0 snap_masked-VaccDscaff30-processed-gene-28.17 maker-VaccDscaff27-snap-gene-91.31 snap_masked-VaccDscaff24-processed-gene-20.11 | Pathogenesis-related leaf protein 4  Indole-3-acetic acid-amido synthetase GH3.10  Mitogen-activated protein kinase kinase 5  Transcription factor TGA2.2  Indole-3-acetic acid-amido synthetase GH3.10  Brassinosteroid Insensitive 1-associated receptor kinase 1  Serine/threonine-protein kinase BSK3  Serine/threonine-protein kinase CTR1  Serine/threonine-protein kinase BSK3  Two-component response regulator ARR8  Brassinosteroid Insensitive 1-associated receptor kinase 1  Two-component response regulator ARR8  Histidine kinase 4  Indole-3-acetic acid-amido synthetase GH3.10  Transcription factor HBP-1b  F-box protein GID2  Histidine-containing phosphotransfer protein 1  Pathogenesis-related leaf protein 4  F-box protein GID2  DELLA protein RGL1  Serine/threonine-protein kinase SRK2A  Histidine-containing phosphotransfer protein AHP1 |
|  |  |  |  |
| ONS1 vs ONS0  ONS2 vs ONS0 | 6  16 | maker-VaccDscaff44-augustus-gene-182.26 maker-VaccDscaff45-snap-gene-80.23 maker-VaccDscaff27-snap-gene-117.30 maker-VaccDscaff21-snap-gene-209.35 maker-VaccDscaff21-augustus-gene-363.31 maker-VaccDscaff41-augustus-gene-47.25  maker-VaccDscaff12-augustus-gene-351.26 maker-VaccDscaff9-augustus-gene-5.32 augustus_masked-VaccDscaff11-processed-gene-28.4 maker-VaccDscaff1-augustus-gene-185.20 maker-VaccDscaff35-snap-gene-180.33 maker-VaccDscaff40-augustus-gene-249.14 maker-VaccDscaff23-augustus-gene-34.21 augustus_masked-VaccDscaff2-processed-gene-15.3 maker-VaccDscaff4-snap-gene-5.49 maker-VaccDscaff18-snap-gene-189.26 maker-VaccDscaff35-snap-gene-165.18 maker-VaccDscaff14-snap-gene-322.46 maker-VaccDscaff37-snap-gene-274.42 augustus_masked-VaccDscaff22-processed-gene-333.6 maker-VaccDscaff48-augustus-gene-100.30 augustus_masked-VaccDscaff3-processed-gene-373.5 | Protein TIFY2  Brassinosteroid Insensitive 1-associated receptor  Transcription factor PIF3  Serine/threonine-protein kinase CTR1  Peter Pan-like protein  Abscisic acid insensitive 5-like protein 5  EIN3-binding F-box protein 1  Two-component response regulator ARR1  Protein Ethylene Insensitive 3  Protein Ethylene Insensitive 2  Two-component response regulator ARR11  EIN3-binding F-box protein 1  EIN3-binding F-box protein 2  Abscisic acid receptor PYR1  Two-component response regulator ARR1  EIN3-binding F-box protein 1  Probable histidine kinase 3  Brassinosteroid insensitive 1-associated receptor  Two-component response regulator ORR22  Ethylene-responsive transcription factor 1B  Protein phosphatase 2C 51  Abscisic acid receptor PYL1 |
|  |  |  |  |
| **Up** |  |  |  |
| BrS2 vs BrS0  BrS1 vs BrS0  BrS0 vs ONS0  ONS1 vs ONS0  ONS2 vs ONS0  BrS2 vs BrS0 | 21  12 | maker-VaccDscaff2-augustus-gene-411.30 augustus_masked-VaccDscaff2-processed-gene-235.6 maker-VaccDscaff27-augustus-gene-335.51 maker-VaccDscaff1050-augustus-gene-0.2 snap_masked-VaccDscaff35-processed-gene-156.16 maker-VaccDscaff124-augustus-gene-1.36 maker-VaccDscaff513-augustus-gene-0.14 maker-VaccDscaff30-augustus-gene-5.33 maker-VaccDscaff18-snap-gene-70.38 maker-VaccDscaff26-snap-gene-12.21 maker-VaccDscaff27-augustus-gene-344.21 maker-VaccDscaff33-snap-gene-304.30 maker-VaccDscaff36-augustus-gene-68.22 maker-VaccDscaff4-augustus-gene-255.25 maker-VaccDscaff36-augustus-gene-199.26 maker-VaccDscaff188-augustus-gene-0.17 maker-VaccDscaff9-augustus-gene-342.31 maker-VaccDscaff17-augustus-gene-121.23 maker-VaccDscaff71-augustus-gene-2.23 maker-VaccDscaff13-augustus-gene-391.28 maker-VaccDscaff9-augustus-gene-217.25  snap_masked-VaccDscaff3-processed-gene-365.11 maker-VaccDscaff17-augustus-gene-20.24 maker-VaccDscaff34-augustus-gene-253.27 maker-VaccDscaff14-augustus-gene-341.56 maker-VaccDscaff27-augustus-gene-84.21 maker-VaccDscaff25-snap-gene-217.25 maker-VaccDscaff9-augustus-gene-244.22 maker-VaccDscaff30-snap-gene-33.47 maker-VaccDscaff17-snap-gene-122.34 maker-VaccDscaff11-augustus-gene-236.26 maker-VaccDscaff35-augustus-gene-29.25 maker-VaccDscaff26-snap-gene-322.41 | Abscisic acid-insensitive 5-like  Protein TIFY 9  Protein TIFY 10A  Indole-3-acetic acid-amido synthetase GH3.6  Protein TIFY 9  Protein TIFY 9  Protein TIFY 10A  Protein TIFY 10A  Serine/threonine-protein kinase SAPK2  Protein TIFY 10A  Protein TIFY 10A  Protein TIFY 10A  Two-component response regulator ARR5  Protein TIFY 9  Protein TIFY 9  Protein TIFY 10A  Two-component response regulator ARR5  Brassinosteroid Insensitive 1-associated receptor kinase 1  Protein TIFY 10A  Protein TIFY 10A  Protein TIFY 9  Protein TIFY 6B  Serine/threonine-protein kinase BSK3  Probable serine/threonine-protein kinase BSK3  Pathogenesis-related leaf protein 4  Brassinosteroid Insensitive 1-associated receptor kinase 1  Serine/threonine-protein kinase BSK  Two-component response regulator ORR26  BTB/POZ domain and ankyrin repeat-containing NPR1  Brassinosteroid Insensitive 1-associated receptor kinase 1  Regulatory protein NPR3  Auxin-responsive protein IAA14  Two-component response regulator ARR8 |
| BrS1 vs BrS0  BrS0 vs ONS0 | 22  44 | maker-VaccDscaff18-augustus-gene-83.29 maker-VaccDscaff12-augustus-gene-351.26 maker-VaccDscaff30-augustus-gene-271.51 maker-VaccDscaff43-augustus-gene-101.28 maker-VaccDscaff6-augustus-gene-323.25 maker-VaccDscaff22-augustus-gene-100.26 maker-VaccDscaff47-augustus-gene-104.20 maker-VaccDscaff7-augustus-gene-33.28 maker-VaccDscaff46-augustus-gene-140.22 maker-VaccDscaff10-snap-gene-292.18 augustus_masked-VaccDscaff14-processed-gene-50.7 maker-VaccDscaff28-snap-gene-12.29 augustus_masked-VaccDscaff22-processed-gene-333.6 maker-VaccDscaff37-augustus-gene-189.24 snap_masked-VaccDscaff11-processed-gene-368.26 augustus_masked-VaccDscaff3-processed-gene-373.5 maker-VaccDscaff37-snap-gene-220.62 maker-VaccDscaff35-augustus-gene-62.23 maker-VaccDscaff39-augustus-gene-97.23 maker-VaccDscaff114-augustus-gene-1.33 augustus_masked-VaccDscaff2-processed-gene-15.3 maker-VaccDscaff6-snap-gene-422.30  snap_masked-VaccDscaff13-processed-gene-392.12 maker-VaccDscaff30-snap-gene-3.34 maker-VaccDscaff20-augustus-gene-11.27 maker-VaccDscaff160-snap-gene-1.36 maker-VaccDscaff20-snap-gene-284.23 augustus_masked-VaccDscaff40-processed-gene-165.5 maker-VaccDscaff46-augustus-gene-177.34 augustus_masked-VaccDscaff19-processed-gene-5.12 maker-VaccDscaff14-augustus-gene-390.37 maker-VaccDscaff14-snap-gene-322.46 maker-VaccDscaff47-snap-gene-169.37 maker-VaccDscaff48-augustus-gene-71.46 maker-VaccDscaff39-augustus-gene-0.29 maker-VaccDscaff13-snap-gene-365.59 augustus_masked-VaccDscaff41-processed-gene-154.5 maker-VaccDscaff45-augustus-gene-36.20 maker-VaccDscaff22-snap-gene-356.44 maker-VaccDscaff29-augustus-gene-142.29 maker-VaccDscaff13-augustus-gene-134.25 maker-VaccDscaff33-augustus-gene-195.31 maker-VaccDscaff20-augustus-gene-12.22 augustus_masked-VaccDscaff23-processed-gene-118.7 maker-VaccDscaff1-augustus-gene-21.22 maker-VaccDscaff34-augustus-gene-232.37 maker-VaccDscaff21-augustus-gene-331.37 augustus_masked-VaccDscaff166-processed-gene-2.12 maker-VaccDscaff13-snap-gene-10.43 maker-VaccDscaff34-snap-gene-193.28 maker-VaccDscaff14-augustus-gene-67.26 maker-VaccDscaff11-augustus-gene-371.31 maker-VaccDscaff4-augustus-gene-336.22 augustus_masked-VaccDscaff17-processed-gene-66.2 maker-VaccDscaff46-snap-gene-106.24 maker-VaccDscaff1-augustus-gene-431.25 maker-VaccDscaff19-augustus-gene-43.40 snap_masked-VaccDscaff159-processed-gene-2.21 maker-VaccDscaff34-augustus-gene-232.36 maker-VaccDscaff27-augustus-gene-311.31 maker-VaccDscaff21-augustus-gene-141.31 maker-VaccDscaff6-augustus-gene-262.13 maker-VaccDscaff1-augustus-gene-47.31 maker-VaccDscaff1-augustus-gene-152.15 augustus_masked-VaccDscaff9-processed-gene-366.5 maker-VaccDscaff39-snap-gene-81.46 | Transcription factor TGA3  EIN3-binding F-box protein 2  Serine/threonine-protein kinase SAPK2  Protein phosphatase 2C  Transcription factor TGA1  Two-component response regulator ARR8  Protein phosphatase 2C 37  BES1/BZR1 homolog protein 2  Protein phosphatase 2C 37  Ethylene-responsive transcription factor 1B  Abscisic acid receptor PYL1  Auxin response factor 9  Ethylene-responsive transcription factor 1B  Transcription factor TGA1  Ethylene-responsive transcription factor 1B  Abscisic acid receptor PYL1  Transcription factor TGA1  Mitogen-activated protein kinase homolog NTF4  Transcription factor TGA1  Gibberellin receptor GID1B  Abscisic acid receptor PYL1  Xyloglucan endotransglucosylase/hydrolase protein 23  Auxin response factor 7  Auxin response factor 19  Protein phosphatase 2C 51  Xyloglucan endotransglucosylase/hydrolase protein 23  EIN3-binding F-box protein 2  Auxin-responsive protein SAUR36  Auxin-induced protein AUX28  Auxin-responsive protein SAUR32  Serine/threonine-protein kinase BSK  Brassinosteroid Insensitive 1-associated receptor kinase 1  Auxin-induced protein 22D  Auxin transporter-like protein 4  Xyloglucan endotransglucosylase/hydrolase 2  BTB/POZ domain and ankyrin repeat-containing NPR1  Auxin-responsive protein SAUR36  Auxin transporter-like protein 4  Histidine-containing phosphotransfer protein AHP1  Protein phosphatase 2C 37  Serine/threonine-protein kinase SRK2E  Protein phosphatase 2C 37  Abscisic acid receptor PYL8  Auxin-responsive protein SAUR36  Two-component response regulator ARR2  Brassinosteroid Insensitive 1-associated receptor kinase 1  Two-component response regulator ARR8  Auxin-responsive protein SAUR72  Transcription factor TGA1  Transcription factor PIF3  Protein TIFY 6B  Auxin-induced protein 22D  Two-component response regulator ARR5  Auxin transporter-like protein 4  Transcription factor TGA9  Indole-3-acetic acid-amido synthetase GH3.6  Auxin transporter protein 1  Auxin response factor 19  Brassinosteroid Insensitive 1-associated receptor kinase 1  Auxin-responsive protein IAA17  Protein phosphatase 2C 37  Auxin response factor 9  Histidine-containing phosphotransfer protein AHP1  Serine/threonine-protein kinase BSK3  Auxin-responsive protein  Serine/threonine-protein kinase SAPK2 |
| ONS1 vs ONS0 | 14 | augustus_masked-VaccDscaff17-processed-gene-121.4 snap_masked-VaccDscaff6-processed-gene-273.12 maker-VaccDscaff20-augustus-gene-363.21 maker-VaccDscaff43-augustus-gene-18.33 augustus_masked-VaccDscaff14-processed-gene-341.10 maker-VaccDscaff8-augustus-gene-370.27 augustus_masked-VaccDscaff32-processed-gene-100.7 augustus_masked-VaccDscaff34-processed-gene-70.1 maker-VaccDscaff15-augustus-gene-231.23 maker-VaccDscaff7-augustus-gene-20.25 augustus_masked-VaccDscaff50-processed-gene-8.9 maker-VaccDscaff3-augustus-gene-197.25 maker-VaccDscaff39-augustus-gene-0.27 maker-VaccDscaff43-snap-gene-247.35 | Brassinosteroid Insensitive 1-associated receptor  Ethylene-insensitive protein 3  Auxin response factor 11  Indole-3-acetic acid-amido synthetase GH3  Pathogenesis-related protein 1A  Histidine-containing phosphotransfer protein 1  Auxin-responsive protein SAUR50  Auxin-responsive protein SAUR32  Regulatory protein NPR3  Cyclin-D3-2  Auxin-responsive protein SAUR71  Histidine kinase 3  Xyloglucan endotransglucosylase/hydrolase protein  Serine/threonine-protein kinase BSK7 |
|  |  |  |  |
| ONS2 vs ONS0 | 24 | augustus_masked-VaccDscaff13-processed-gene-21.8 maker-VaccDscaff17-augustus-gene-265.28 maker-VaccDscaff23-augustus-gene-312.33 maker-VaccDscaff239-snap-gene-0.62 maker-VaccDscaff30-augustus-gene-220.32 maker-VaccDscaff266-augustus-gene-0.64 augustus_masked-VaccDscaff13-processed-gene-7.3 augustus_masked-VaccDscaff14-processed-gene-381.8 snap_masked-VaccDscaff31-processed-gene-294.12 maker-VaccDscaff36-augustus-gene-16.42 maker-VaccDscaff3-augustus-gene-357.27 maker-VaccDscaff33-snap-gene-15.29 maker-VaccDscaff20-augustus-gene-155.11 maker-VaccDscaff19-augustus-gene-248.32 maker-VaccDscaff26-snap-gene-135.26 maker-VaccDscaff41-augustus-gene-40.31 maker-VaccDscaff27-snap-gene-82.32 maker-VaccDscaff33-augustus-gene-205.23 maker-VaccDscaff14-snap-gene-147.28 augustus_masked-VaccDscaff12-processed-gene-26.1 maker-VaccDscaff5-augustus-gene-212.22 snap_masked-VaccDscaff4-processed-gene-279.10 maker-VaccDscaff30-augustus-gene-222.17 augustus_masked-VaccDscaff12-processed-gene-55.3 | DELLA protein GAI  Two-component response regulator ARR5  Cyclin-D3-3  Auxin transporter-like protein 2  BES1/BZR1 homolog protein 2  Cyclin-D3-1  Brassinosteroid LRR receptor kinase  Auxin-responsive protein SAUR50  Auxin-responsive protein IAA8  Auxin-responsive protein IAA26  DELLA protein GAI1  Transcription factor TGA10  Histidine kinase 4  Transport inhibitor response 1-like protein  Coronatine-insensitive protein 1  Cyclin-D3-3  Auxin-induced protein X10A  Coronatine-insensitive protein 1  Auxin transporter-like protein 3  Auxin-responsive protein SAUR50  Auxin transporter-like protein 2  Two-component response regulator ARR11  BES1/BZR1 homolog protein 2  Auxin-responsive protein SAUR50 |

**Table S5.** The expression levels and foldchanges of specific DEGs related to plant hormone signal transduction (ko04075) pathway.

| **Gene_ID** | **FPKM** | |  | **FPKM** | |  | **FPKM** | |  |  |
| --- | --- | --- | --- | --- | --- | --- | --- | --- | --- | --- |
|  | **BrSO** | **ONS0** | **Log_2_(ratio)(BrS0/ONS0)** | **BrS1** | **ONS1** | **Log_2_(ratio)(BrS1/ONS1)** | **BrS2** | **ONS2** | **Log_2_(ratio)(BrS2/ONS2)** | **Functional annotation** |
| **Auxin-related DEGs** |  |  |  |  |  |  |  |  |  |  |
| maker-VaccDscaff19-augustus-gene-43.40 | 10.37 | 0.61 | 4.09 | 3.35 | 0.70 | 2.26 | 3.09 | 0.53 | 2.54 | Auxin transporter protein 1 |
| maker-VaccDscaff48-augustus-gene-71.45 | 2.77 | 1.08 | 1.36 | 0.68 | 0.66 | 0.04 | 0.68 | 0.60 | 0.19 | Auxin transporter protein 1 |
| maker-VaccDscaff5-augustus-gene-212.22 | 1.30 | 1.81 | -0.47 | 1.04 | 2.77 | -1.42 | 1.43 | 7.67 | -2.42 | Auxin transporter-like protein 2 |
| maker-VaccDscaff1-augustus-gene-212.15 | 7.39 | 4.64 | 0.67 | 8.91 | 0.31 | 4.84 | 10.78 | 1.06 | 3.34 | Auxin transporter-like protein 2 |
| maker-VaccDscaff14-snap-gene-147.28 | 0.33 | 0.03 | 3.27 | 0.08 | 0.30 | -1.93 | 0.03 | 0.95 | -4.96 | Auxin transporter-like protein 3 |
| augustus_masked-VaccDscaff17-processed-gene-66.2 | 5.87 | 1.61 | 1.87 | 1.33 | 0.79 | 0.75 | 1.17 | 1.60 | -0.45 | Auxin transporter-like protein 4 |
| maker-VaccDscaff48-augustus-gene-71.46 | 18.90 | 5.63 | 1.75 | 7.02 | 5.76 | 0.29 | 5.34 | 4.95 | 0.11 | Auxin transporter-like protein 4 |
| maker-VaccDscaff19-augustus-gene-43.41 | 53.18 | 22.61 | 1.23 | 19.60 | 4.34 | 2.17 | 14.69 | 4.75 | 1.63 | Auxin transporter-like protein 4 |
| maker-VaccDscaff20-augustus-gene-43.48 | 8.12 | 3.01 | 1.43 | 3.44 | 5.05 | -0.55 | 1.87 | 4.42 | -1.24 | Auxin transporter-like protein 4 |
| maker-VaccDscaff27-augustus-gene-27.21 | 3.22 | 9.42 | -1.55 | 0.71 | 2.55 | -1.84 | 0.65 | 3.71 | -2.52 | Auxin transporter-like protein 4 |
| maker-VaccDscaff28-augustus-gene-311.42 | 35.07 | 40.74 | -0.22 | 11.63 | 8.29 | 0.49 | 8.89 | 8.05 | 0.14 | Auxin transporter-like protein 4 |
| maker-VaccDscaff28-snap-gene-312.54 | 19.40 | 11.21 | 0.79 | 4.72 | 2.35 | 1.01 | 4.07 | 2.86 | 0.51 | Auxin transporter-like protein 4 |
| maker-VaccDscaff34-augustus-gene-264.25 | 6.93 | 4.40 | 0.66 | 1.23 | 1.19 | 0.05 | 1.05 | 1.23 | -0.23 | Auxin transporter-like protein 4 |
| maker-VaccDscaff45-augustus-gene-36.20 | 6.92 | 1.93 | 1.84 | 2.06 | 1.92 | 0.10 | 1.63 | 4.27 | -1.39 | Auxin transporter-like protein 4 |
| maker-VaccDscaff239-snap-gene-0.62 | 1.29 | 1.12 | 0.21 | 0.49 | 1.36 | -1.47 | 0.70 | 3.67 | -2.38 | Auxin transporter-like protein 5 |
| maker-VaccDscaff15-augustus-gene-159.11 | 5.56 | 3.60 | 0.63 | 8.00 | 0.37 | 4.42 | 8.65 | 0.64 | 3.77 | Protein Transport Inhibitor Response TIR1 |
| maker-VaccDscaff19-augustus-gene-248.32 | 1.30 | 2.51 | -0.96 | 1.64 | 3.49 | -1.09 | 2.63 | 9.05 | -1.78 | Protein Transport Inhibitor Response TIR1 |
| maker-VaccDscaff24-augustus-gene-214.28 | 4.78 | 4.83 | -0.02 | 7.12 | 0.90 | 2.99 | 7.81 | 1.49 | 2.39 | Protein Transport Inhibitor Response TIR1 |
| maker-VaccDscaff1-snap-gene-141.13 | 1.85 | 0.04 | 5.63 | 0.82 | 0.57 | 0.53 | 1.54 | 1.38 | 0.15 | Auxin-responsive protein IAA13 |
| maker-VaccDscaff5-augustus-gene-128.15 | 0.71 | 0.84 | -0.24 | 0.16 | 2.79 | -4.16 | 0.82 | 4.58 | -2.48 | Auxin-responsive protein IAA13 |
| maker-VaccDscaff8-snap-gene-285.27 | 1.14 | 0.26 | 2.16 | 0.51 | 1.29 | -1.35 | 0.69 | 1.67 | -1.28 | Auxin-responsive protein IAA13 |
| maker-VaccDscaff1-augustus-gene-93.20 | 2.13 | 0.66 | 1.70 | 11.38 | 6.46 | 0.82 | 38.10 | 15.04 | 1.34 | Auxin-responsive protein IAA14 |
| maker-VaccDscaff5-augustus-gene-105.11 | 0.99 | 0.60 | 0.72 | 4.45 | 7.17 | -0.69 | 15.81 | 15.89 | -0.01 | Auxin-responsive protein IAA14 |
| maker-VaccDscaff10-augustus-gene-319.37 | 1.00 | 0.53 | 0.92 | 3.38 | 9.58 | -1.50 | 13.32 | 21.53 | -0.69 | Auxin-responsive protein IAA14 |
| maker-VaccDscaff34-snap-gene-34.29 | 5.04 | 4.15 | 0.28 | 3.55 | 25.16 | -2.83 | 6.56 | 30.24 | -2.21 | Auxin-responsive protein IAA14 |
| maker-VaccDscaff35-augustus-gene-29.25 | 0.94 | 1.06 | -0.17 | 2.09 | 0.35 | 2.58 | 5.30 | 1.58 | 1.75 | Auxin-responsive protein IAA14 |
| maker-VaccDscaff36-augustus-gene-278.31 | 0.50 | 0.20 | 1.33 | 0.90 | 0.16 | 2.54 | 2.39 | 1.10 | 1.12 | Auxin-responsive protein IAA14 |
| maker-VaccDscaff4-augustus-gene-59.29 | 3.48 | 0.48 | 2.87 | 6.64 | 0.18 | 5.24 | 18.32 | 1.49 | 3.62 | Auxin-responsive protein IAA14 |
| maker-VaccDscaff8-augustus-gene-332.14 | 0.41 | 0.52 | -0.35 | 2.14 | 3.32 | -0.64 | 9.15 | 6.96 | 0.39 | Auxin-responsive protein IAA14 |
| maker-VaccDscaff9-augustus-gene-36.33 | 1.58 | 0.10 | 4.03 | 2.78 | 0.03 | 6.57 | 8.95 | 0.02 | 8.72 | Auxin-responsive protein IAA14 |
| maker-VaccDscaff17-augustus-gene-348.31 | 12.34 | 3.40 | 1.86 | 9.16 | 32.59 | -1.83 | 17.19 | 46.90 | -1.45 | Auxin-responsive protein IAA17 |
| maker-VaccDscaff27-augustus-gene-311.31 | 3.89 | 0.09 | 5.47 | 2.76 | 0.00 | / | 6.41 | 0.00 | / | Auxin-responsive protein IAA17 |
| maker-VaccDscaff4-snap-gene-426.46 | 1.98 | 0.05 | 5.29 | 2.91 | 3.76 | -0.37 | 2.27 | 4.02 | -0.83 | Auxin-responsive protein IAA17 |
| maker-VaccDscaff9-snap-gene-390.42 | 1.08 | 0.02 | 6.12 | 2.07 | 0.99 | 1.05 | 2.12 | 1.31 | 0.69 | Auxin-responsive protein IAA17 |
| maker-VaccDscaff17-augustus-gene-358.28 | 11.19 | 8.01 | 0.48 | 25.92 | 0.09 | 8.14 | 8.61 | 0.01 | 9.52 | Auxin-responsive protein IAA26 |
| maker-VaccDscaff35-augustus-gene-307.25 | 5.04 | 1.68 | 1.58 | 7.06 | 5.13 | 0.46 | 4.08 | 7.05 | -0.79 | Auxin-responsive protein IAA26 |
| maker-VaccDscaff36-augustus-gene-16.42 | 2.73 | 1.37 | 0.99 | 5.08 | 4.03 | 0.33 | 2.84 | 6.13 | -1.11 | Auxin-responsive protein IAA26 |
| maker-VaccDscaff4-snap-gene-419.43 | 3.44 | 1.53 | 1.17 | 5.74 | 7.88 | -0.46 | 2.94 | 12.06 | -2.04 | Auxin-responsive protein IAA26 |
| maker-VaccDscaff9-snap-gene-396.39 | 3.52 | 0.68 | 2.37 | 6.67 | 6.41 | 0.06 | 3.93 | 10.00 | -1.35 | Auxin-responsive protein IAA26 |
| maker-VaccDscaff13-snap-gene-321.31 | 10.31 | 7.75 | 0.41 | 12.40 | 29.12 | -1.23 | 23.95 | 35.57 | -0.57 | Auxin-responsive protein IAA27 |
| maker-VaccDscaff21-augustus-gene-2.29 | 5.86 | 7.02 | -0.26 | 1.90 | 5.52 | -1.54 | 10.73 | 17.95 | -0.74 | Auxin-responsive protein IAA27 |
| maker-VaccDscaff27-augustus-gene-318.43 | 24.29 | 19.99 | 0.28 | 15.61 | 0.00 | / | 17.62 | 0.10 | 7.43 | Auxin-responsive protein IAA27 |
| maker-VaccDscaff32-augustus-gene-10.32 | 5.10 | 14.06 | -1.46 | 1.96 | 0.51 | 1.94 | 2.79 | 0.28 | 3.31 | Auxin-responsive protein IAA27 |
| maker-VaccDscaff9-augustus-gene-394.16 | 35.35 | 40.45 | -0.19 | 26.73 | 1.25 | 4.42 | 27.33 | 1.37 | 4.31 | Auxin-responsive protein IAA27 |
| snap_masked-VaccDscaff31-processed-gene-294.12 | 6.18 | 8.01 | -0.38 | 4.26 | 18.54 | -2.12 | 4.87 | 26.97 | -2.47 | Auxin-responsive protein IAA8 |
| maker-VaccDscaff18-augustus-gene-271.38 | 1.43 | 3.39 | -1.24 | 8.46 | 17.37 | -1.04 | 10.52 | 24.83 | -1.24 | Auxin-responsive protein IAA9 |
| maker-VaccDscaff31-augustus-gene-33.35 | 9.24 | 13.05 | -0.50 | 26.90 | 46.21 | -0.78 | 33.13 | 66.73 | -1.01 | Auxin-responsive protein IAA9 |
| maker-VaccDscaff7-snap-gene-309.31 | 3.09 | 4.72 | -0.61 | 6.27 | 15.93 | -1.34 | 8.04 | 23.13 | -1.52 | Auxin-responsive protein IAA9 |
| maker-VaccDscaff27-snap-gene-82.32 | 0.27 | 0.25 | 0.10 | 1.88 | 0.72 | 1.38 | 0.99 | 2.43 | -1.29 | Auxin-induced protein 15A |
| maker-VaccDscaff11-augustus-gene-371.31 | 4.91 | 0.83 | 2.56 | 6.30 | 0.68 | 3.21 | 14.09 | 1.68 | 3.07 | Auxin-induced protein 22D |
| maker-VaccDscaff19-augustus-gene-374.28 | 4.74 | 0.75 | 2.66 | 4.01 | 17.90 | -2.16 | 8.21 | 38.08 | -2.21 | Auxin-induced protein 22D |
| maker-VaccDscaff24-augustus-gene-31.42 | 8.52 | 0.94 | 3.18 | 8.53 | 11.13 | -0.38 | 18.73 | 19.10 | -0.03 | Auxin-induced protein 22D |
| maker-VaccDscaff43-augustus-gene-31.40 | 30.68 | 7.15 | 2.10 | 10.31 | 23.88 | -1.21 | 22.80 | 23.03 | -0.01 | Auxin-induced protein 22D |
| maker-VaccDscaff47-snap-gene-169.37 | 27.64 | 3.12 | 3.15 | 8.61 | 7.94 | 0.12 | 17.86 | 7.64 | 1.22 | Auxin-induced protein 22D |
| augustus_masked-VaccDscaff12-processed-gene-56.0 | 1.66 | 1.76 | -0.08 | 0.17 | 0.25 | -0.58 | 0.00 | 0.22 | / | Auxin-induced protein 6B |
| snap_masked-VaccDscaff23-processed-gene-356.11 | 4.69 | 1.80 | 1.38 | 0.27 | 0.20 | 0.44 | 0.00 | 0.27 | / | Auxin-induced protein 6B |
| maker-VaccDscaff43-augustus-gene-31.37 | 1.37 | 0.24 | 2.54 | 0.20 | 0.38 | -0.93 | 1.06 | 1.14 | -0.10 | Auxin-induced protein AUX28 |
| maker-VaccDscaff46-augustus-gene-177.34 | 0.83 | 0.12 | 2.84 | 0.09 | 0.13 | -0.49 | 0.48 | 0.28 | 0.77 | Auxin-induced protein AUX28 |
| maker-VaccDscaff47-augustus-gene-168.31 | 0.67 | 0.13 | 2.33 | 0.09 | 0.09 | 0.04 | 0.50 | 0.33 | 0.59 | Auxin-induced protein AUX28 |
| maker-VaccDscaff17-snap-gene-119.32 | 0.32 | 0.06 | 2.40 | 1.80 | 0.69 | 1.39 | 1.18 | 1.60 | -0.44 | Auxin-induced protein X10A |
| augustus_masked-VaccDscaff19-processed-gene-5.12 | 29.91 | 4.64 | 2.69 | 32.43 | 4.73 | 2.78 | 22.36 | 2.67 | 3.07 | Auxin-responsive protein SAUR32 |
| augustus_masked-VaccDscaff20-processed-gene-7.2 | 127.32 | 15.74 | 3.02 | 137.14 | 67.36 | 1.03 | 131.26 | 36.22 | 1.86 | Auxin-responsive protein SAUR32 |
| augustus_masked-VaccDscaff21-processed-gene-31.0 | 0.10 | 4.02 | -5.39 | 0.06 | 0.04 | 0.61 | 0.06 | 0.00 | / | Auxin-responsive protein SAUR32 |
| augustus_masked-VaccDscaff48-processed-gene-104.2 | 67.88 | 3.94 | 4.11 | 92.98 | 65.00 | 0.52 | 76.55 | 34.93 | 1.13 | Auxin-responsive protein SAUR32 |
| augustus_masked-VaccDscaff23-processed-gene-118.7 | 4.17 | 0.83 | 2.33 | 3.03 | 1.92 | 0.66 | 1.48 | 2.26 | -0.61 | Auxin-responsive protein SAUR36 |
| augustus_masked-VaccDscaff40-processed-gene-165.5 | 2.23 | 0.45 | 2.31 | 3.20 | 0.92 | 1.81 | 1.15 | 0.80 | 0.52 | Auxin-responsive protein SAUR36 |
| augustus_masked-VaccDscaff41-processed-gene-154.5 | 2.31 | 0.64 | 1.86 | 2.64 | 1.00 | 1.40 | 1.13 | 1.06 | 0.09 | Auxin-responsive protein SAUR36 |
| snap_masked-VaccDscaff22-processed-gene-41.8 | 14.67 | 5.73 | 1.36 | 3.79 | 3.59 | 0.08 | 3.03 | 3.99 | -0.40 | Auxin-responsive protein SAUR36 |
| augustus_masked-VaccDscaff14-processed-gene-381.8 | 2.26 | 0.90 | 1.33 | 3.25 | 1.45 | 1.17 | 4.54 | 7.70 | -0.76 | Auxin-responsive protein SAUR50 |
| augustus_masked-VaccDscaff12-processed-gene-26.1 | 0.71 | 0.37 | 0.92 | 0.47 | 1.66 | -1.82 | 0.21 | 11.14 | -5.72 | Auxin-responsive protein SAUR50 |
| augustus_masked-VaccDscaff12-processed-gene-55.3 | 0.70 | 0.37 | 0.90 | 0.52 | 1.63 | -1.65 | 0.24 | 10.88 | -5.49 | Auxin-responsive protein SAUR50 |
| augustus_masked-VaccDscaff30-processed-gene-107.2 | 9.11 | 26.00 | -1.51 | 10.08 | 5.02 | 1.00 | 6.04 | 4.67 | 0.37 | Auxin-responsive protein SAUR50 |
| augustus_masked-VaccDscaff151-processed-gene-2.2 | 2.06 | 0.53 | 1.97 | 1.88 | 3.93 | -1.06 | 1.18 | 1.61 | -0.45 | Auxin-responsive protein SAUR72 |
| augustus_masked-VaccDscaff166-processed-gene-2.12 | 3.04 | 0.65 | 2.22 | 0.22 | 0.46 | -1.05 | 0.11 | 0.53 | -2.22 | Auxin-responsive protein SAUR72 |
| augustus_masked-VaccDscaff62-processed-gene-4.7 | 9.99 | 4.50 | 1.15 | 0.80 | 0.52 | 0.62 | 0.20 | 0.26 | -0.33 | Auxin-responsive protein SAUR72 |
| augustus_masked-VaccDscaff78-processed-gene-3.7 | 2.48 | 2.27 | 0.13 | 0.20 | 0.62 | -1.61 | 0.23 | 0.75 | -1.74 | Auxin-responsive protein SAUR72 |
| maker-VaccDscaff14-augustus-gene-351.35 | 55.27 | 2.05 | 4.75 | 25.29 | 13.60 | 0.90 | 19.92 | 13.31 | 0.58 | Indole-3-acetic acid-amido synthetase GH3.1 |
| maker-VaccDscaff15-snap-gene-362.39 | 0.90 | 0.00 | / | 7.97 | 0.00 | / | 0.22 | 0.00 | / | Indole-3-acetic acid-amido synthetase GH3.1 |
| maker-VaccDscaff2-augustus-gene-404.32 | 20.95 | 2.66 | 2.98 | 12.08 | 22.39 | -0.89 | 9.97 | 22.59 | -1.18 | Indole-3-acetic acid-amido synthetase GH3.1 |
| maker-VaccDscaff3-augustus-gene-11.36 | 34.20 | 6.22 | 2.46 | 17.68 | 32.22 | -0.87 | 15.32 | 29.03 | -0.92 | Indole-3-acetic acid-amido synthetase GH3.1 |
| maker-VaccDscaff10-augustus-gene-90.15 | 0.56 | 2.64 | -2.24 | 0.38 | 1.39 | -1.87 | 0.56 | 1.12 | -0.99 | Indole-3-acetic acid-amido synthetase GH3.10 |
| maker-VaccDscaff5-snap-gene-323.24 | 0.32 | 1.56 | -2.28 | 0.20 | 0.88 | -2.17 | 0.30 | 0.79 | -1.41 | Indole-3-acetic acid-amido synthetase GH3.10 |
| maker-VaccDscaff8-snap-gene-111.37 | 0.50 | 2.62 | -2.38 | 0.25 | 2.21 | -3.16 | 0.52 | 2.76 | -2.41 | Indole-3-acetic acid-amido synthetase GH3.10 |
| maker-VaccDscaff1050-augustus-gene-0.2 | 0.76 | 0.23 | 1.74 | 5.83 | 4.73 | 0.30 | 4.00 | 5.32 | -0.41 | Indole-3-acetic acid-amido synthetase GH3.6 |
| maker-VaccDscaff1-augustus-gene-431.25 | 0.46 | 0.00 | / | 0.53 | 0.13 | 2.04 | 0.17 | 0.10 | 0.82 | Indole-3-acetic acid-amido synthetase GH3.6 |
| maker-VaccDscaff22-augustus-gene-355.20 | 1.38 | 0.71 | 0.97 | 0.49 | 0.58 | -0.27 | 0.44 | 0.96 | -1.12 | Indole-3-acetic acid-amido synthetase GH3.6 |
| maker-VaccDscaff47-augustus-gene-180.31 | 1.14 | 2.16 | -0.93 | 0.36 | 0.52 | -0.55 | 0.56 | 0.63 | -0.17 | Indole-3-acetic acid-amido synthetase GH3.6 |
| maker-VaccDscaff6-augustus-gene-55.29 | 0.67 | 0.26 | 1.34 | 6.49 | 4.84 | 0.43 | 3.85 | 5.74 | -0.58 | Indole-3-acetic acid-amido synthetase GH3.6 |
| snap_masked-VaccDscaff159-processed-gene-2.21 | 18.13 | 1.76 | 3.37 | 4.79 | 1.99 | 1.26 | 4.36 | 2.42 | 0.85 | Auxin response factor 19 |
| maker-VaccDscaff30-snap-gene-3.34 | 12.75 | 1.53 | 3.06 | 2.80 | 2.20 | 0.35 | 2.66 | 3.02 | -0.19 | Auxin response factor 19 |
| snap_masked-VaccDscaff71-processed-gene-4.10 | 16.81 | 15.06 | 0.16 | 3.51 | 3.30 | 0.09 | 3.28 | 4.92 | -0.58 | Auxin response factor 19 |
| maker-VaccDscaff12-snap-gene-56.27 | 4.48 | 5.08 | -0.18 | 2.19 | 2.70 | -0.30 | 0.90 | 5.47 | -2.60 | Auxin response factor 5 |
| maker-VaccDscaff23-snap-gene-355.33 | 13.13 | 6.26 | 1.07 | 7.71 | 4.03 | 0.94 | 3.87 | 7.61 | -0.98 | Auxin response factor 5 |
| maker-VaccDscaff23-augustus-gene-349.16 | 9.72 | 7.14 | 0.45 | 5.47 | 2.27 | 1.27 | 2.64 | 5.15 | -0.97 | Auxin response factor 5 |
| snap_masked-VaccDscaff13-processed-gene-392.12 | 9.84 | 1.31 | 2.91 | 1.99 | 2.28 | -0.20 | 1.94 | 2.68 | -0.46 | Auxin response factor 7 |
| maker-VaccDscaff28-snap-gene-12.29 | 1.24 | 1.96 | -0.66 | 4.26 | 1.69 | 1.33 | 1.81 | 1.15 | 0.65 | Auxin response factor 9 |
| maker-VaccDscaff38-augustus-gene-161.11 | 10.11 | 5.40 | 0.91 | 4.94 | 5.44 | -0.14 | 3.28 | 5.70 | -0.79 | Auxin response factor 9 |
| maker-VaccDscaff49-augustus-gene-18.25 | 0.93 | 0.38 | 1.29 | 2.97 | 1.90 | 0.65 | 1.38 | 1.25 | 0.14 | Auxin response factor 9 |
| maker-VaccDscaff6-augustus-gene-262.13 | 4.30 | 1.32 | 1.70 | 2.33 | 1.97 | 0.25 | 1.94 | 2.77 | -0.51 | Auxin response factor 9 |
| augustus_masked-VaccDscaff14-processed-gene-381.3 | 0.95 | 0.80 | 0.25 | 2.50 | 2.91 | -0.22 | 1.48 | 4.70 | -1.67 | Auxin-responsive protein |
| augustus_masked-VaccDscaff35-processed-gene-282.5 | 0.67 | 2.23 | -1.74 | 0.97 | 0.18 | 2.46 | 0.33 | 0.08 | 2.05 | Auxin-responsive protein |
| augustus_masked-VaccDscaff9-processed-gene-366.5 | 6.71 | 1.71 | 1.97 | 13.69 | 0.78 | 4.13 | 2.46 | 0.12 | 4.32 | Auxin-responsive protein |
| maker-VaccDscaff16-snap-gene-123.42 | 7.43 | 13.61 | -0.87 | 20.28 | 45.92 | -1.18 | 27.87 | 63.96 | -1.20 | Auxin-responsive protein |
| **Cytokinin-related DEGs** |  |  |  |  |  |  |  |  |  |  |
| maker-VaccDscaff4-augustus-gene-263.18 | 6.82 | 10.46 | -0.62 | 4.32 | 4.27 | 0.02 | 1.97 | 3.28 | -0.73 | Histidine kinase 3 |
| maker-VaccDscaff35-snap-gene-165.18 | 4.81 | 6.27 | -0.38 | 3.36 | 2.29 | 0.56 | 2.15 | 2.05 | 0.07 | Histidine kinase 3 |
| maker-VaccDscaff9-snap-gene-226.23 | 4.54 | 1.66 | 1.45 | 2.31 | 1.76 | 0.39 | 1.35 | 1.45 | -0.11 | Histidine kinase 3 |
| maker-VaccDscaff20-augustus-gene-155.11 | 0.20 | 0.36 | -0.86 | 0.21 | 0.50 | -1.26 | 0.15 | 1.39 | -3.18 | Histidine kinase 4 |
| maker-VaccDscaff28-augustus-gene-168.15 | 0.17 | 0.01 | 3.77 | 0.20 | 0.31 | -0.63 | 0.15 | 0.65 | -2.07 | Histidine kinase 4 |
| maker-VaccDscaff44-augustus-gene-170.17 | 0.12 | 0.39 | -1.69 | 0.13 | 0.17 | -0.37 | 0.08 | 0.51 | -2.74 | Histidine kinase 4 |
| maker-VaccDscaff51-snap-gene-21.65 | 2.07 | 0.34 | 2.61 | 3.96 | 2.86 | 0.47 | 3.41 | 3.40 | 0.01 | Histidine-containing phosphotransfer protein 1 |
| maker-VaccDscaff8-augustus-gene-370.27 | 0.63 | 5.19 | -3.04 | 0.96 | 19.70 | -4.36 | 1.59 | 14.56 | -3.19 | Histidine-containing phosphotransfer protein 1 |
| snap_masked-VaccDscaff24-processed-gene-20.11 | 0.77 | 3.71 | -2.26 | 1.44 | 2.91 | -1.02 | 1.04 | 3.93 | -1.92 | Histidine-containing phosphotransfer protein 1 |
| maker-VaccDscaff1-augustus-gene-47.31 | 1.52 | 0.31 | 2.31 | 2.68 | 0.63 | 2.08 | 3.39 | 0.40 | 3.09 | Histidine-containing phosphotransfer protein 1 |
| maker-VaccDscaff22-snap-gene-356.44 | 0.66 | 0.04 | 4.09 | 0.66 | 0.35 | 0.89 | 0.69 | 0.16 | 2.14 | Histidine-containing phosphotransfer protein 1 |
| maker-VaccDscaff5-augustus-gene-39.35 | 1.65 | 2.42 | -0.55 | 3.56 | 22.56 | -2.67 | 8.45 | 13.99 | -0.73 | Histidine-containing phosphotransfer protein 1 |
| maker-VaccDscaff7-augustus-gene-189.30 | 0.99 | 0.41 | 1.28 | 1.05 | 1.28 | -0.30 | 1.44 | 2.04 | -0.50 | Histidine-containing phosphotransfer protein 1 |
| maker-VaccDscaff4-snap-gene-5.49 | 2.82 | 12.83 | -2.19 | 2.83 | 2.18 | 0.37 | 0.32 | 2.00 | -2.65 | Two-component response regulator ARR1 |
| maker-VaccDscaff9-augustus-gene-5.32 | 8.22 | 12.73 | -0.63 | 6.71 | 4.94 | 0.44 | 7.05 | 4.17 | 0.76 | Two-component response regulator ARR1 |
| maker-VaccDscaff4-snap-gene-9.54 | 6.49 | 1.49 | 2.12 | 5.16 | 6.54 | -0.34 | 5.46 | 5.53 | -0.02 | Two-component response regulator ARR1 |
| maker-VaccDscaff1-augustus-gene-21.22 | 7.83 | 1.72 | 2.19 | 19.59 | 1.76 | 3.48 | 10.22 | 2.01 | 2.35 | Two-component response regulator ARR2 |
| maker-VaccDscaff8-snap-gene-394.21 | 5.21 | 4.22 | 0.30 | 13.48 | 13.07 | 0.04 | 6.47 | 13.94 | -1.11 | Two-component response regulator ARR2 |
| maker-VaccDscaff36-augustus-gene-68.22 | 2.83 | 0.29 | 3.28 | 12.78 | 8.53 | 0.58 | 24.52 | 7.57 | 1.70 | Two-component response regulator ARR5 |
| maker-VaccDscaff36-augustus-gene-75.30 | 8.71 | 11.62 | -0.41 | 86.42 | 63.58 | 0.44 | 156.40 | 57.78 | 1.44 | Two-component response regulator ARR5 |
| maker-VaccDscaff17-augustus-gene-265.28 | 3.20 | 1.45 | 1.15 | 0.98 | 3.93 | -2.00 | 2.65 | 6.11 | -1.20 | Two-component response regulator ARR5 |
| maker-VaccDscaff27-augustus-gene-241.33 | 1.21 | 0.55 | 1.13 | 0.03 | 2.27 | -6.36 | 0.56 | 3.38 | -2.60 | Two-component response regulator ARR5 |
| maker-VaccDscaff34-augustus-gene-112.18 | 13.50 | 1.63 | 3.05 | 5.89 | 4.21 | 0.48 | 9.43 | 6.58 | 0.52 | Two-component response regulator ARR5 |
| maker-VaccDscaff36-augustus-gene-71.27 | 3.32 | 4.60 | -0.47 | 42.31 | 67.26 | -0.67 | 84.84 | 63.94 | 0.41 | Two-component response regulator ARR5 |
| maker-VaccDscaff9-augustus-gene-342.31 | 2.98 | 0.93 | 1.68 | 24.86 | 10.56 | 1.24 | 36.89 | 8.36 | 2.14 | Two-component response regulator ARR5 |
| maker-VaccDscaff1301-augustus-gene-0.5 | 0.72 | 2.30 | -1.67 | 1.95 | 1.48 | 0.40 | 1.57 | 1.03 | 0.61 | Two-component response regulator ARR8 |
| maker-VaccDscaff21-augustus-gene-331.37 | 12.06 | 2.59 | 2.22 | 3.83 | 1.58 | 1.28 | 18.62 | 6.93 | 1.43 | Two-component response regulator ARR8 |
| maker-VaccDscaff22-augustus-gene-100.26 | 0.59 | 2.70 | -2.20 | 1.91 | 1.20 | 0.67 | 1.52 | 0.90 | 0.74 | Two-component response regulator ARR8 |
| maker-VaccDscaff26-snap-gene-322.41 | 6.58 | 5.81 | 0.18 | 4.36 | 4.47 | -0.04 | 20.64 | 15.24 | 0.44 | Two-component response regulator ARR8 |
| snap_masked-VaccDscaff1-processed-gene-280.9 | 4.62 | 3.56 | 0.38 | 1.95 | 0.01 | 7.41 | 3.94 | 0.09 | 5.39 | Two-component response regulator ARR9 |
| maker-VaccDscaff31-snap-gene-168.18 | 0.71 | 0.28 | 1.32 | 0.36 | 0.51 | -0.51 | 0.07 | 0.74 | -3.50 | Two-component response regulator ORR22 |
| maker-VaccDscaff153-augustus-gene-1.40 | 0.82 | 0.08 | 3.29 | 2.64 | 0.36 | 2.87 | 1.87 | 0.63 | 1.56 | Two-component response regulator ORR23 |
| maker-VaccDscaff37-snap-gene-274.42 | 0.22 | 0.46 | -1.07 | 0.32 | 0.03 | 3.21 | 0.10 | 0.08 | 0.38 | Two-component response regulator ORR24 |
| snap_masked-VaccDscaff4-processed-gene-279.10 | 0.55 | 1.10 | -0.99 | 0.99 | 2.31 | -1.22 | 1.56 | 6.36 | -2.03 | Two-component response regulator ORR26 |
| maker-VaccDscaff35-snap-gene-180.33 | 0.53 | 0.84 | -0.66 | 1.03 | 0.04 | 4.69 | 1.46 | 0.00 | / | Two-component response regulator ORR26 |
| maker-VaccDscaff9-augustus-gene-244.22 | 0.45 | 0.09 | 2.33 | 1.18 | 0.08 | 3.96 | 1.59 | 0.07 | 4.49 | Two-component response regulator ORR26 |
| maker-VaccDscaff14-augustus-gene-137.27 | 0.19 | 0.09 | 1.06 | 2.42 | 8.32 | -1.78 | 7.39 | 21.41 | -1.53 | Two-component response regulator ORR4 |
| maker-VaccDscaff25-augustus-gene-215.12 | 0.10 | 0.10 | -0.04 | 0.82 | 1.54 | -0.92 | 1.85 | 3.04 | -0.72 | Two-component response regulator ORR4 |
| maker-VaccDscaff2-augustus-gene-196.15 | 0.13 | 0.04 | 1.88 | 1.73 | 2.20 | -0.35 | 5.55 | 4.27 | 0.38 | Two-component response regulator ORR4 |
| maker-VaccDscaff3-augustus-gene-246.16 | 0.32 | 0.07 | 2.25 | 2.46 | 4.41 | -0.84 | 7.19 | 14.07 | -0.97 | Two-component response regulator ORR4 |
| **Gibberellin-related DEGs** |  |  |  |  |  |  |  |  |  |  |
| augustus_masked-VaccDscaff23-processed-gene-307.0 | 1.28 | 4.41 | -1.78 | 2.08 | 22.39 | -3.43 | 2.17 | 33.99 | -3.97 | F-box protein GID2 |
| augustus_masked-VaccDscaff18-processed-gene-7.7 | 73.39 | 90.59 | -0.30 | 16.67 | 48.56 | -1.54 | 8.17 | 23.78 | -1.54 | F-box protein GID2 |
| augustus_masked-VaccDscaff40-processed-gene-34.7 | 1.38 | 10.13 | -2.88 | 2.46 | 5.29 | -1.10 | 3.46 | 7.69 | -1.15 | F-box protein GID2 |
| augustus_masked-VaccDscaff7-processed-gene-13.6 | 204.12 | 370.66 | -0.86 | 34.90 | 46.89 | -0.43 | 17.32 | 22.41 | -0.37 | F-box protein GID2 |
| augustus_masked-VaccDscaff7-processed-gene-19.5 | 59.50 | 77.30 | -0.38 | 22.10 | 12.94 | 0.77 | 12.31 | 7.09 | 0.80 | F-box protein GID2 |
| maker-VaccDscaff114-augustus-gene-1.33 | 4.86 | 4.01 | 0.28 | 17.77 | 10.81 | 0.72 | 5.50 | 2.76 | 1.00 | Gibberellin receptor GID1B |
| maker-VaccDscaff11-augustus-gene-383.35 | 11.08 | 5.93 | 0.90 | 42.92 | 22.99 | 0.90 | 12.46 | 5.75 | 1.12 | Gibberellin receptor GID1B |
| maker-VaccDscaff24-augustus-gene-19.32 | 12.33 | 6.52 | 0.92 | 49.11 | 25.72 | 0.93 | 14.05 | 6.86 | 1.03 | Gibberellin receptor GID1B |
| maker-VaccDscaff51-augustus-gene-20.32 | 14.04 | 1.83 | 2.94 | 54.80 | 27.52 | 0.99 | 16.51 | 5.71 | 1.53 | Gibberellin receptor GID1B |
| maker-VaccDscaff27-augustus-gene-156.15 | 9.36 | 12.79 | -0.45 | 3.77 | 3.32 | 0.18 | 3.05 | 3.68 | -0.27 | Gibberellin receptor GID1C |
| maker-VaccDscaff45-augustus-gene-162.21 | 8.48 | 10.92 | -0.37 | 3.28 | 2.22 | 0.56 | 2.70 | 0.91 | 1.58 | Gibberellin receptor GID1C |
| augustus_masked-VaccDscaff13-processed-gene-21.8 | 9.34 | 6.77 | 0.46 | 12.42 | 11.96 | 0.06 | 10.50 | 25.45 | -1.28 | DELLA protein GAI |
| maker-VaccDscaff3-augustus-gene-357.27 | 1.74 | 3.58 | -1.04 | 2.05 | 9.43 | -2.20 | 1.94 | 13.02 | -2.75 | DELLA protein GAI1 |
| snap_masked-VaccDscaff30-processed-gene-28.17 | 2.09 | 9.98 | -2.26 | 14.13 | 118.55 | -3.07 | 12.07 | 82.76 | -2.78 | DELLA protein RGL1 |
| maker-VaccDscaff34-snap-gene-193.28 | 6.35 | 1.83 | 1.80 | 3.95 | 1.98 | 0.99 | 3.85 | 2.15 | 0.84 | Transcription factor PIF3 |
| maker-VaccDscaff45-snap-gene-115.21 | 1.44 | 5.30 | -1.89 | 0.84 | 0.16 | 2.43 | 1.03 | 0.23 | 2.18 | Transcription factor PIF3 |
| **Ethylene-related DEGs** |  |  |  |  |  |  |  |  |  |  |
| maker-VaccDscaff1-augustus-gene-104.11 | 26.72 | 48.90 | -0.87 | 38.31 | 14.00 | 1.45 | 21.21 | 6.47 | 1.71 | Ethylene receptor 2 |
| maker-VaccDscaff5-snap-gene-88.22 | 20.87 | 78.20 | -1.91 | 22.13 | 44.13 | -1.00 | 11.59 | 20.76 | -0.84 | Ethylene receptor 2 |
| maker-VaccDscaff33-augustus-gene-45.29 | 9.82 | 9.33 | 0.07 | 4.41 | 5.36 | -0.28 | 2.04 | 7.18 | -1.82 | Serine/threonine-protein kinase CTR1 |
| maker-VaccDscaff21-snap-gene-249.39 | 3.80 | 10.28 | -1.44 | 2.04 | 0.14 | 3.91 | 2.26 | 0.01 | 7.60 | Serine/threonine-protein kinase CTR1 |
| maker-VaccDscaff29-snap-gene-197.22 | 1.75 | 5.40 | -1.63 | 3.00 | 3.23 | -0.11 | 3.27 | 3.60 | -0.14 | Serine/threonine-protein kinase CTR1 |
| augustus_masked-VaccDscaff15-processed-gene-37.4 | 3.19 | 4.46 | -0.49 | 3.57 | 1.00 | 1.84 | 2.40 | 0.56 | 2.11 | Ethylene Insensitive 3-like |
| augustus_masked-VaccDscaff37-processed-gene-159.4 | 1.86 | 0.41 | 2.17 | 1.42 | 2.26 | -0.68 | 2.13 | 1.14 | 0.90 | Ethylene Insensitive 3-like |
| augustus_masked-VaccDscaff39-processed-gene-154.3 | 2.47 | 0.36 | 2.77 | 1.87 | 2.45 | -0.39 | 2.95 | 1.09 | 1.43 | Ethylene Insensitive 3-like |
| augustus_masked-VaccDscaff11-processed-gene-28.4 | 6.80 | 13.64 | -1.00 | 8.13 | 7.01 | 0.21 | 4.69 | 3.52 | 0.41 | Ethylene Insensitive 3-like 3 |
| snap_masked-VaccDscaff17-processed-gene-92.9 | 4.98 | 3.43 | 0.54 | 5.50 | 0.98 | 2.48 | 2.68 | 0.89 | 1.58 | Ethylene-insensitive protein 2 |
| snap_masked-VaccDscaff45-processed-gene-7.19 | 4.60 | 4.67 | -0.02 | 5.51 | 0.90 | 2.62 | 2.72 | 0.96 | 1.50 | Ethylene-insensitive protein 2 |
| maker-VaccDscaff10-augustus-gene-225.23 | 3.35 | 0.33 | 3.36 | 1.64 | 1.90 | -0.21 | 2.16 | 1.94 | 0.15 | Ethylene-insensitive protein 2 |
| maker-VaccDscaff1-augustus-gene-185.20 | 10.00 | 9.53 | 0.07 | 7.33 | 0.82 | 3.17 | 7.43 | 2.64 | 1.49 | Ethylene-Insensitive protein 2 |
| maker-VaccDscaff16-augustus-gene-215.21 | 18.05 | 21.19 | -0.23 | 20.48 | 3.35 | 2.61 | 12.92 | 1.22 | 3.40 | EIN3-binding F-box protein 2 |
| maker-VaccDscaff12-augustus-gene-351.26 | 11.22 | 19.68 | -0.81 | 34.50 | 29.50 | 0.23 | 7.14 | 3.70 | 0.95 | EIN3-binding F-box protein 2 |
| maker-VaccDscaff18-snap-gene-189.26 | 7.99 | 4.93 | 0.70 | 9.75 | 2.60 | 1.90 | 6.48 | 1.04 | 2.63 | EIN3-binding F-box protein 2 |
| maker-VaccDscaff20-snap-gene-284.23 | 5.61 | 1.38 | 2.02 | 15.31 | 2.53 | 2.60 | 8.70 | 1.59 | 2.46 | EIN3-binding F-box protein 2 |
| maker-VaccDscaff23-augustus-gene-34.21 | 14.54 | 31.49 | -1.11 | 42.42 | 31.83 | 0.41 | 9.06 | 3.81 | 1.25 | EIN3-binding F-box protein 2 |
| maker-VaccDscaff40-augustus-gene-249.14 | 12.56 | 14.86 | -0.24 | 35.98 | 17.06 | 1.08 | 8.10 | 2.18 | 1.89 | EIN3-binding F-box protein 2 |
| maker-VaccDscaff11-snap-gene-390.50 | 12.74 | 26.52 | -1.06 | 7.11 | 4.69 | 0.60 | 4.13 | 3.83 | 0.11 | Ethylene Response Sensor 1 |
| maker-VaccDscaff15-augustus-gene-382.31 | 21.11 | 34.79 | -0.72 | 11.35 | 4.47 | 1.34 | 6.76 | 3.88 | 0.80 | Ethylene Response Sensor 1 |
| maker-VaccDscaff24-augustus-gene-7.31 | 45.26 | 85.40 | -0.92 | 24.80 | 17.90 | 0.47 | 14.42 | 14.73 | -0.03 | Ethylene Response Sensor 1 |
| augustus_masked-VaccDscaff15-processed-gene-360.12 | 0.48 | 1.47 | -1.62 | 17.54 | 1.59 | 3.47 | 10.83 | 0.84 | 3.69 | Ethylene-responsive factor 1B |
| augustus_masked-VaccDscaff22-processed-gene-333.6 | 5.22 | 3.71 | 0.49 | 40.63 | 5.73 | 2.83 | 10.18 | 0.77 | 3.72 | Ethylene-responsive factor 1B |
| augustus_masked-VaccDscaff43-processed-gene-39.7 | 3.57 | 2.15 | 0.73 | 30.95 | 13.29 | 1.22 | 9.58 | 1.44 | 2.73 | Ethylene-responsive factor 1B |
| augustus_masked-VaccDscaff46-processed-gene-169.9 | 3.06 | 0.41 | 2.88 | 27.10 | 5.24 | 2.37 | 9.90 | 0.82 | 3.59 | Ethylene-responsive factor 1B |
| augustus_masked-VaccDscaff47-processed-gene-160.6 | 3.49 | 1.90 | 0.88 | 27.57 | 6.77 | 2.03 | 8.71 | 1.21 | 2.85 | Ethylene-responsive factor 1B |
| augustus_masked-VaccDscaff8-processed-gene-302.5 | 0.54 | 0.17 | 1.70 | 2.23 | 1.76 | 0.34 | 0.77 | 0.16 | 2.25 | Ethylene-responsive factor 1B |
| maker-VaccDscaff5-snap-gene-77.26 | 0.40 | 0.19 | 1.06 | 2.12 | 1.39 | 0.61 | 0.80 | 0.10 | 3.06 | Ethylene-responsive factor 1B |
| maker-VaccDscaff10-snap-gene-292.18 | 0.17 | 0.08 | 1.19 | 1.30 | 0.56 | 1.22 | 0.43 | 0.03 | 3.66 | Ethylene-responsive factor 1B |
| maker-VaccDscaff1-snap-gene-111.21 | 1.95 | 0.42 | 2.21 | 7.14 | 0.64 | 3.48 | 2.23 | 0.01 | 7.61 | Ethylene-responsive factor 1B |
| maker-VaccDscaff24-augustus-gene-34.39 | 0.67 | 0.66 | 0.03 | 24.84 | 7.65 | 1.70 | 15.65 | 2.92 | 2.42 | Ethylene-responsive factor 1B |
| snap_masked-VaccDscaff11-processed-gene-368.26 | 0.31 | 1.13 | -1.87 | 5.30 | 2.93 | 0.86 | 2.12 | 1.24 | 0.77 | Ethylene-responsive factor 1B |
| augustus_masked-VaccDscaff4-processed-gene-79.1 | 1.02 | 0.41 | 1.31 | 2.35 | 4.00 | -0.77 | 1.19 | 1.26 | -0.08 | Ethylene-responsive factor 2 |
| **Abscisic acid-related DEGs** |  |  |  |  |  |  |  |  |  |  |
| augustus_masked-VaccDscaff2-processed-gene-15.3 | 3.81 | 4.77 | -0.32 | 16.18 | 2.63 | 2.62 | 3.55 | 1.19 | 1.58 | Abscisic acid receptor PYL1 |
| augustus_masked-VaccDscaff14-processed-gene-50.7 | 1.78 | 1.74 | 0.03 | 9.15 | 0.51 | 4.18 | 1.36 | 0.18 | 2.89 | Abscisic acid receptor PYL1 |
| augustus_masked-VaccDscaff3-processed-gene-373.5 | 0.72 | 1.89 | -1.39 | 3.15 | 1.39 | 1.18 | 0.80 | 0.42 | 0.94 | Abscisic acid receptor PYL1 |
| augustus_masked-VaccDscaff20-processed-gene-36.4 | 3.48 | 3.82 | -0.13 | 1.34 | 0.32 | 2.08 | 0.44 | 0.11 | 2.03 | Abscisic acid receptor PYL4 |
| augustus_masked-VaccDscaff28-processed-gene-319.6 | 0.01 | 19.74 | -10.94 | 0.01 | 0.01 | 0.05 | 0.00 | 0.04 | / | Abscisic acid receptor PYL4 |
| augustus_masked-VaccDscaff45-processed-gene-54.1 | 33.44 | 84.30 | -1.33 | 3.73 | 11.55 | -1.63 | 7.93 | 3.47 | 1.19 | Abscisic acid receptor PYL4 |
| snap_masked-VaccDscaff27-processed-gene-14.16 | 48.64 | 28.56 | 0.77 | 5.28 | 12.80 | -1.28 | 11.52 | 4.43 | 1.38 | Abscisic acid receptor PYL4 |
| maker-VaccDscaff28-augustus-gene-339.21 | 8.00 | 26.32 | -1.72 | 10.35 | 6.06 | 0.77 | 8.13 | 5.28 | 0.62 | Abscisic acid receptor PYL8 |
| maker-VaccDscaff20-augustus-gene-12.22 | 36.40 | 11.43 | 1.67 | 30.14 | 7.58 | 1.99 | 17.61 | 8.18 | 1.11 | Abscisic acid receptor PYL8 |
| maker-VaccDscaff41-augustus-gene-184.24 | 7.37 | 7.24 | 0.03 | 5.03 | 0.79 | 2.66 | 2.15 | 0.61 | 1.82 | Abscisic acid receptor PYL8 |
| maker-VaccDscaff12-augustus-gene-302.25 | 19.26 | 20.77 | -0.11 | 10.05 | 2.19 | 2.20 | 5.78 | 1.83 | 1.66 | Abscisic acid receptor PYL9 |
| maker-VaccDscaff22-augustus-gene-7.18 | 21.38 | 36.81 | -0.78 | 85.45 | 87.75 | -0.04 | 140.89 | 63.88 | 1.14 | Abscisic acid receptor PYL9 |
| maker-VaccDscaff23-augustus-gene-89.30 | 20.31 | 47.15 | -1.22 | 10.90 | 5.23 | 1.06 | 6.02 | 4.44 | 0.44 | Abscisic acid receptor PYL9 |
| maker-VaccDscaff40-augustus-gene-196.22 | 0.53 | 2.62 | -2.31 | 0.32 | 1.25 | -1.97 | 0.19 | 0.86 | -2.16 | Abscisic acid receptor PYL9 |
| snap_masked-VaccDscaff1257-processed-gene-0.1 | 10.82 | 26.60 | -1.30 | 65.14 | 58.73 | 0.15 | 100.93 | 44.25 | 1.19 | Abscisic acid receptor PYL9 |
| snap_masked-VaccDscaff1279-processed-gene-0.1 | 21.28 | 29.39 | -0.47 | 71.41 | 64.79 | 0.14 | 126.20 | 47.88 | 1.40 | Abscisic acid receptor PYL9 |
| maker-VaccDscaff13-augustus-gene-15.39 | 1.91 | 0.49 | 1.96 | 5.50 | 4.80 | 0.20 | 2.60 | 7.70 | -1.57 | Serine/threonine-protein kinase SAPK1 |
| maker-VaccDscaff13-snap-gene-15.44 | 5.84 | 7.35 | -0.33 | 0.16 | 0.29 | -0.82 | 0.08 | 0.12 | -0.62 | Serine/threonine-protein kinase SAPK1 |
| maker-VaccDscaff42-augustus-gene-46.27 | 2.46 | 0.90 | 1.45 | 6.45 | 4.63 | 0.48 | 3.79 | 5.49 | -0.53 | Serine/threonine-protein kinase SAPK1 |
| maker-VaccDscaff42-augustus-gene-46.33 | 6.58 | 17.06 | -1.37 | 0.17 | 0.09 | 0.89 | 0.19 | 0.09 | 1.11 | Serine/threonine-protein kinase SAPK1 |
| maker-VaccDscaff16-snap-gene-187.25 | 4.49 | 5.64 | -0.33 | 1.98 | 1.16 | 0.77 | 1.91 | 0.50 | 1.93 | Serine/threonine-protein kinase SAPK10 |
| maker-VaccDscaff30-augustus-gene-271.51 | 0.49 | 0.04 | 3.48 | 1.80 | 0.26 | 2.78 | 1.03 | 0.39 | 1.41 | Serine/threonine-protein kinase SAPK2 |
| maker-VaccDscaff7-augustus-gene-78.36 | 1.08 | 1.32 | -0.29 | 5.79 | 15.30 | -1.40 | 6.82 | 13.86 | -1.02 | Serine/threonine-protein kinase SAPK2 |
| maker-VaccDscaff16-snap-gene-328.35 | 3.45 | 2.28 | 0.60 | 17.12 | 8.27 | 1.05 | 21.15 | 7.31 | 1.53 | Serine/threonine-protein kinase SAPK2 |
| maker-VaccDscaff18-snap-gene-70.38 | 6.17 | 2.04 | 1.60 | 20.85 | 13.10 | 0.67 | 24.72 | 13.65 | 0.86 | Serine/threonine-protein kinase SAPK2 |
| maker-VaccDscaff39-snap-gene-81.46 | 1.72 | 0.16 | 3.42 | 2.73 | 1.09 | 1.33 | 2.80 | 1.14 | 1.30 | Serine/threonine-protein kinase SAPK2 |
| maker-VaccDscaff6-snap-gene-342.30 | 2.36 | 0.43 | 2.45 | 2.87 | 1.64 | 0.81 | 3.51 | 1.35 | 1.38 | Serine/threonine-protein kinase SAPK2 |
| maker-VaccDscaff17-augustus-gene-130.28 | 5.55 | 7.00 | -0.34 | 5.35 | 25.82 | -2.27 | 6.08 | 27.76 | -2.19 | Serine/threonine-protein kinase SRK2A |
| maker-VaccDscaff25-augustus-gene-362.19 | 1.29 | 14.04 | -3.44 | 1.68 | 1.44 | 0.22 | 2.95 | 1.38 | 1.09 | Serine/threonine-protein kinase SRK2A |
| maker-VaccDscaff27-snap-gene-91.31 | 3.68 | 16.82 | -2.19 | 3.58 | 17.52 | -2.29 | 2.76 | 18.60 | -2.75 | Serine/threonine-protein kinase SRK2A |
| maker-VaccDscaff13-augustus-gene-134.25 | 6.57 | 0.44 | 3.90 | 9.40 | 1.04 | 3.17 | 8.63 | 0.14 | 5.94 | Serine/threonine-protein kinase SRK2E |
| maker-VaccDscaff32-augustus-gene-236.28 | 2.69 | 5.12 | -0.93 | 5.57 | 1.01 | 2.46 | 4.99 | 0.24 | 4.39 | Serine/threonine-protein kinase SRK2E |
| maker-VaccDscaff96-snap-gene-2.43 | 1.60 | 3.11 | -0.95 | 0.70 | 0.29 | 1.25 | 0.55 | 0.11 | 2.26 | Serine/threonine-protein kinase SRK2I |
| maker-VaccDscaff13-augustus-gene-376.23 | 24.17 | 33.16 | -0.46 | 15.91 | 6.22 | 1.36 | 13.17 | 7.29 | 0.85 | Protein phosphatase 2C 16 |
| maker-VaccDscaff30-augustus-gene-23.27 | 16.76 | 24.73 | -0.56 | 10.67 | 7.41 | 0.53 | 6.34 | 6.50 | -0.04 | Protein phosphatase 2C 16 |
| maker-VaccDscaff32-augustus-gene-60.19 | 22.40 | 38.30 | -0.77 | 14.01 | 7.65 | 0.87 | 12.08 | 7.02 | 0.78 | Protein phosphatase 2C 16 |
| maker-VaccDscaff16-augustus-gene-253.20 | 1.05 | 0.31 | 1.75 | 1.97 | 1.36 | 0.54 | 1.50 | 1.77 | -0.24 | Protein phosphatase 2C 3 |
| maker-VaccDscaff31-augustus-gene-198.19 | 0.41 | 0.37 | 0.17 | 0.67 | 3.64 | -2.45 | 0.93 | 3.50 | -1.91 | Protein phosphatase 2C 3 |
| maker-VaccDscaff47-augustus-gene-104.20 | 0.27 | 0.01 | 4.75 | 1.10 | 0.34 | 1.71 | 0.33 | 0.31 | 0.10 | Protein phosphatase 2C 37 |
| snap_masked-VaccDscaff20-processed-gene-48.29 | 6.93 | 0.92 | 2.92 | 15.73 | 8.59 | 0.87 | 11.56 | 11.06 | 0.06 | Protein phosphatase 2C 37 |
| maker-VaccDscaff19-augustus-gene-49.35 | 30.90 | 1.85 | 4.06 | 82.72 | 29.65 | 1.48 | 62.05 | 36.95 | 0.75 | Protein phosphatase 2C 37 |
| maker-VaccDscaff21-augustus-gene-141.31 | 18.18 | 5.78 | 1.65 | 37.17 | 6.45 | 2.53 | 10.38 | 6.36 | 0.71 | Protein phosphatase 2C 37 |
| maker-VaccDscaff29-augustus-gene-142.29 | 11.31 | 2.99 | 1.92 | 21.80 | 5.22 | 2.06 | 6.18 | 5.94 | 0.06 | Protein phosphatase 2C 37 |
| maker-VaccDscaff33-augustus-gene-195.31 | 15.88 | 3.86 | 2.04 | 35.62 | 5.51 | 2.69 | 8.93 | 5.42 | 0.72 | Protein phosphatase 2C 37 |
| maker-VaccDscaff43-augustus-gene-101.28 | 0.31 | 0.02 | 3.81 | 1.28 | 0.11 | 3.52 | 0.27 | 0.10 | 1.41 | Protein phosphatase 2C 37 |
| maker-VaccDscaff46-augustus-gene-140.22 | 0.18 | 0.06 | 1.45 | 1.15 | 0.08 | 3.77 | 0.27 | 0.13 | 1.06 | Protein phosphatase 2C 37 |
| maker-VaccDscaff20-augustus-gene-11.27 | 6.46 | 0.44 | 3.87 | 6.21 | 0.27 | 4.55 | 2.56 | 0.10 | 4.62 | Protein phosphatase 2C 51 |
| augustus_masked-VaccDscaff28-processed-gene-340.8 | 2.26 | 2.65 | -0.23 | 3.72 | 0.58 | 2.69 | 1.17 | 0.16 | 2.85 | Protein phosphatase 2C 51 |
| maker-VaccDscaff48-augustus-gene-100.30 | 2.34 | 0.91 | 1.36 | 2.40 | 0.53 | 2.19 | 0.83 | 0.10 | 3.13 | Protein phosphatase 2C 51 |
| augustus_masked-VaccDscaff31-processed-gene-337.9 | 0.00 | 0.04 | / | 8.95 | 0.04 | 7.82 | 1.69 | 0.01 | 7.18 | Abscisic acid Insensitive 5-like |
| maker-VaccDscaff14-augustus-gene-358.38 | 0.84 | 0.63 | 0.42 | 5.99 | 1.58 | 1.92 | 4.25 | 3.65 | 0.22 | Abscisic acid Insensitive 5-like |
| maker-VaccDscaff169-augustus-gene-0.20 | 0.23 | 0.09 | 1.37 | 42.53 | 0.86 | 5.63 | 8.69 | 0.51 | 4.10 | Abscisic acid Insensitive 5-like |
| maker-VaccDscaff18-augustus-gene-2.21 | 0.12 | 0.00 | / | 10.10 | 0.22 | 5.49 | 1.83 | 0.19 | 3.27 | Abscisic acid Insensitive 5-like |
| maker-VaccDscaff2-augustus-gene-411.30 | 1.68 | 0.28 | 2.61 | 9.98 | 2.34 | 2.09 | 8.26 | 5.19 | 0.67 | Abscisic acid Insensitive 5-like |
| maker-VaccDscaff40-augustus-gene-24.16 | 9.81 | 11.51 | -0.23 | 10.32 | 2.24 | 2.20 | 5.40 | 3.07 | 0.82 | Abscisic acid Insensitive 5-like |
| maker-VaccDscaff7-snap-gene-24.27 | 0.00 | 0.00 | / | 6.10 | 0.09 | 6.12 | 1.19 | 0.07 | 4.15 | Abscisic acid Insensitive 5-like |
| maker-VaccDscaff982-snap-gene-0.6 | 1.84 | 2.84 | -0.62 | 10.36 | 7.05 | 0.56 | 7.13 | 15.98 | -1.16 | Abscisic acid Insensitive 5-like |
| **Jasmonate-related DEGs** |  |  |  |  |  |  |  |  |  |  |
| maker-VaccDscaff22-snap-gene-8.34 | 10.95 | 12.22 | -0.16 | 51.47 | 25.84 | 0.99 | 50.30 | 17.48 | 1.52 | Jasmonic acid-amido synthetase JAR1 |
| augustus_masked-VaccDscaff20-processed-gene-43.7 | 11.25 | 5.04 | 1.16 | 3.94 | 2.23 | 0.82 | 3.09 | 1.87 | 0.72 | Coronatine-insensitive protein 1 |
| maker-VaccDscaff26-snap-gene-135.26 | 0.48 | 0.72 | -0.61 | 0.55 | 0.90 | -0.70 | 0.92 | 2.34 | -1.34 | Coronatine-insensitive protein 1 |
| maker-VaccDscaff33-augustus-gene-205.23 | 2.02 | 1.55 | 0.39 | 2.83 | 2.85 | -0.01 | 2.84 | 5.47 | -0.95 | Coronatine-insensitive protein 1 |
| maker-VaccDscaff48-augustus-gene-71.44 | 9.78 | 10.36 | -0.08 | 3.76 | 1.09 | 1.78 | 2.79 | 0.87 | 1.67 | Coronatine-insensitive protein 1 |
| snap_masked-VaccDscaff19-processed-gene-44.20 | 14.20 | 21.21 | -0.58 | 4.66 | 5.07 | -0.12 | 4.24 | 4.78 | -0.17 | Coronatine-insensitive protein 1 |
| **maker-VaccDscaff13-augustus-gene-391.28** | **47.03** | **2.07** | **4.50** | **248.19** | **82.06** | **1.60** | **262.55** | **61.61** | **2.09** | **Protein TIFY 10A** |
| **maker-VaccDscaff30-augustus-gene-5.33** | **99.60** | **9.89** | **3.33** | **449.60** | **157.69** | **1.51** | **459.64** | **119.45** | **1.94** | **Protein TIFY 10A** |
| **maker-VaccDscaff188-augustus-gene-0.17** | **114.84** | **10.74** | **3.42** | **505.35** | **249.31** | **1.02** | **529.72** | **205.50** | **1.37** | **Protein TIFY 10A** |
| **maker-VaccDscaff21-snap-gene-15.39** | **44.36** | **4.69** | **3.24** | **155.78** | **140.70** | **0.15** | **195.39** | **125.26** | **0.64** | **Protein TIFY 10A** |
| **maker-VaccDscaff26-snap-gene-12.21** | **51.56** | **5.42** | **3.25** | **191.93** | **128.83** | **0.58** | **221.31** | **114.48** | **0.95** | **Protein TIFY 10A** |
| **maker-VaccDscaff27-augustus-gene-335.51** | **30.08** | **1.03** | **4.87** | **110.15** | **56.82** | **0.96** | **100.50** | **21.59** | **2.22** | **Protein TIFY 10A** |
| **maker-VaccDscaff27-augustus-gene-344.21** | **31.13** | **3.30** | **3.24** | **132.46** | **185.97** | **-0.49** | **120.37** | **96.78** | **0.31** | **Protein TIFY 10A** |
| **maker-VaccDscaff29-snap-gene-11.26** | **79.62** | **40.58** | **0.97** | **230.77** | **123.85** | **0.90** | **260.08** | **108.65** | **1.26** | **Protein TIFY 10A** |
| **maker-VaccDscaff33-snap-gene-304.30** | **83.06** | **22.08** | **1.91** | **314.16** | **221.33** | **0.51** | **343.49** | **192.75** | **0.83** | **Protein TIFY 10A** |
| **maker-VaccDscaff513-augustus-gene-0.14** | **69.51** | **5.10** | **3.77** | **288.66** | **231.46** | **0.32** | **272.05** | **123.57** | **1.14** | **Protein TIFY 10A** |
| **maker-VaccDscaff71-augustus-gene-2.23** | **36.15** | **3.47** | **3.38** | **169.93** | **202.67** | **-0.25** | **173.61** | **163.43** | **0.09** | **Protein TIFY 10A like** |
| **snap_masked-VaccDscaff3-processed-gene-365.11** | **49.24** | **81.04** | **-0.72** | **145.23** | **173.63** | **-0.26** | **154.30** | **160.37** | **-0.06** | **Protein TIFY 6B** |
| **maker-VaccDscaff14-augustus-gene-67.26** | **59.04** | **17.84** | **1.73** | **148.73** | **24.64** | **2.59** | **150.09** | **21.23** | **2.82** | **Protein TIFY 6B** |
| **augustus_masked-VaccDscaff2-processed-gene-235.6** | **3.48** | **0.44** | **2.99** | **233.87** | **201.36** | **0.22** | **266.12** | **218.62** | **0.28** | **Protein TIFY 9** |
| **maker-VaccDscaff124-augustus-gene-0.28** | **0.19** | **0.00** | **/** | **14.32** | **162.64** | **-3.51** | **18.15** | **178.57** | **-3.30** | **Protein TIFY 9** |
| **maker-VaccDscaff124-augustus-gene-1.36** | **10.41** | **2.07** | **2.33** | **563.33** | **196.16** | **1.52** | **701.60** | **208.67** | **1.75** | **Protein TIFY 9** |
| **maker-VaccDscaff14-snap-gene-196.42** | **0.61** | **0.00** | **/** | **61.54** | **53.60** | **0.20** | **67.85** | **46.85** | **0.53** | **Protein TIFY 9** |
| **maker-VaccDscaff36-augustus-gene-199.26** | **3.89** | **0.05** | **6.21** | **121.73** | **88.76** | **0.46** | **122.72** | **39.23** | **1.65** | **Protein TIFY 9** |
| **maker-VaccDscaff4-augustus-gene-255.25** | **1.89** | **0.38** | **2.33** | **95.57** | **206.49** | **-1.11** | **94.96** | **84.05** | **0.18** | **Protein TIFY 9** |
| **maker-VaccDscaff9-augustus-gene-217.25** | **2.07** | **0.02** | **6.40** | **90.49** | **31.21** | **1.54** | **91.81** | **10.85** | **3.08** | **Protein TIFY 9** |
| **snap_masked-VaccDscaff35-processed-gene-156.16** | **0.52** | **0.00** | **/** | **20.94** | **14.13** | **0.57** | **20.67** | **5.96** | **1.79** | **Protein TIFY 9** |
| **Salicylic acid-related DEGs** |  |  |  |  |  |  |  |  |  |  |
| augustus_masked-VaccDscaff2-processed-gene-357.7 | 0.00 | 0.84 | / | 0.00 | 0.00 | / | 0.00 | 0.00 | / | Disease resistance protein |
| maker-VaccDscaff10-augustus-gene-89.22 | 1.02 | 0.53 | 0.94 | 1.62 | 3.96 | -1.29 | 1.40 | 3.59 | -1.36 | Disease resistance protein |
| maker-VaccDscaff14-augustus-gene-258.14 | 0.30 | 0.24 | 0.34 | 0.57 | 1.61 | -1.49 | 0.67 | 3.01 | -2.17 | Disease resistance protein |
| maker-VaccDscaff14-augustus-gene-333.25 | 0.17 | 0.02 | 3.07 | 0.41 | 1.55 | -1.92 | 0.44 | 1.55 | -1.81 | Disease resistance protein |
| maker-VaccDscaff14-augustus-gene-352.35 | 0.75 | 0.26 | 1.50 | 0.96 | 0.91 | 0.08 | 1.02 | 0.93 | 0.13 | Disease resistance protein |
| maker-VaccDscaff14-snap-gene-333.33 | 0.28 | 0.15 | 0.86 | 0.53 | 1.14 | -1.09 | 0.64 | 1.13 | -0.84 | Disease resistance protein |
| maker-VaccDscaff29-snap-gene-288.28 | 1.04 | 8.72 | -3.06 | 2.28 | 0.06 | 5.14 | 1.97 | 0.09 | 4.44 | Disease resistance protein |
| maker-VaccDscaff2-augustus-gene-357.31 | 2.62 | 1.22 | 1.11 | 10.48 | 5.43 | 0.95 | 11.46 | 5.12 | 1.16 | Disease resistance protein |
| maker-VaccDscaff2-augustus-gene-386.31 | 1.72 | 1.69 | 0.02 | 1.86 | 0.39 | 2.27 | 1.70 | 0.30 | 2.52 | Disease resistance protein |
| maker-VaccDscaff2-augustus-gene-387.28 | 0.52 | 0.07 | 2.80 | 1.20 | 0.70 | 0.78 | 1.58 | 0.66 | 1.25 | Disease resistance protein |
| maker-VaccDscaff2-snap-gene-387.32 | 0.17 | 0.01 | 4.25 | 0.20 | 0.85 | -2.11 | 0.33 | 0.89 | -1.45 | Disease resistance protein |
| maker-VaccDscaff2-snap-gene-394.55 | 0.14 | 0.05 | 1.32 | 0.22 | 0.20 | 0.13 | 0.39 | 0.20 | 0.93 | Disease resistance protein |
| maker-VaccDscaff34-snap-gene-215.33 | 1.57 | 0.24 | 2.74 | 4.23 | 3.40 | 0.32 | 5.38 | 3.03 | 0.83 | Disease resistance protein |
| maker-VaccDscaff3-snap-gene-19.25 | 0.16 | 0.04 | 1.91 | 0.22 | 2.50 | -3.50 | 0.46 | 2.08 | -2.18 | Disease resistance protein |
| maker-VaccDscaff3-snap-gene-20.25 | 0.03 | 0.03 | 0.29 | 0.11 | 1.16 | -3.39 | 0.27 | 1.09 | -2.01 | Disease resistance protein |
| snap_masked-VaccDscaff25-processed-gene-299.17 | 0.22 | 0.68 | -1.61 | 0.20 | 7.86 | -5.32 | 0.28 | 9.70 | -5.13 | Disease resistance protein |
| augustus_masked-VaccDscaff1-processed-gene-270.9 | 1.04 | 1.14 | -0.12 | 2.49 | 0.00 | / | 4.81 | 0.00 | / | Disease resistance protein |
| maker-VaccDscaff2-augustus-gene-385.40 | 0.36 | 0.03 | 3.56 | 0.71 | 0.70 | 0.03 | 1.14 | 0.58 | 0.97 | Disease resistance protein |
| maker-VaccDscaff3-snap-gene-22.55 | 0.37 | 0.55 | -0.58 | 0.73 | 1.83 | -1.33 | 0.83 | 2.21 | -1.41 | Disease resistance protein |
| snap_masked-VaccDscaff25-processed-gene-354.16 | 0.00 | 2.00 | / | 0.00 | 0.00 | / | 0.00 | 0.00 | / | Disease resistance protein |
| maker-VaccDscaff39-augustus-gene-275.30 | 1.43 | 0.60 | 1.26 | 7.06 | 7.53 | -0.09 | 14.86 | 7.49 | 0.99 | Disease resistance protein |
| maker-VaccDscaff3-augustus-gene-14.36 | 0.09 | 0.65 | -2.91 | 0.12 | 2.65 | -4.47 | 0.10 | 2.56 | -4.64 | Disease resistance protein |
| maker-VaccDscaff3-augustus-gene-19.19 | 0.28 | 0.14 | 1.06 | 0.51 | 1.08 | -1.09 | 0.54 | 0.85 | -0.65 | Disease resistance protein |
| maker-VaccDscaff3-snap-gene-13.29 | 0.22 | 0.47 | -1.12 | 0.30 | 1.56 | -2.38 | 0.37 | 1.42 | -1.94 | Disease resistance protein |
| maker-VaccDscaff3-snap-gene-18.31 | 0.02 | 0.21 | -3.17 | 0.08 | 2.98 | -5.25 | 0.19 | 2.37 | -3.60 | Disease resistance protein |
| augustus_masked-VaccDscaff14-processed-gene-332.5 | 0.15 | 0.11 | 0.40 | 0.20 | 0.55 | -1.46 | 0.36 | 0.45 | -0.34 | Disease resistance protein |
| augustus_masked-VaccDscaff634-processed-gene-0.1 | 0.80 | 0.21 | 1.92 | 0.46 | 0.96 | -1.07 | 0.75 | 1.68 | -1.16 | Disease resistance protein RFL1 |
| maker-VaccDscaff25-snap-gene-354.34 | 0.00 | 3.77 | / | 0.00 | 0.00 | / | 0.00 | 0.00 | / | Disease resistance protein RFL1 |
| maker-VaccDscaff2-augustus-gene-393.35 | 1.90 | 0.09 | 4.48 | 1.93 | 0.73 | 1.41 | 2.33 | 0.85 | 1.44 | Disease resistance protein RFL1 |
| maker-VaccDscaff6-augustus-gene-376.18 | 0.75 | 1.69 | -1.17 | 1.20 | 5.98 | -2.32 | 1.23 | 4.89 | -1.99 | Disease resistance protein RFL1 |
| maker-VaccDscaff4-snap-gene-140.55 | 2.45 | 1.69 | 0.54 | 7.17 | 0.00 | / | 7.89 | 0.00 | / | Disease resistance protein RPM1 |
| maker-VaccDscaff13-augustus-gene-294.22 | 0.07 | 0.05 | 0.46 | 0.04 | 1.23 | -4.80 | 0.05 | 0.89 | -4.07 | Disease resistance protein RPM1 |
| maker-VaccDscaff9-snap-gene-94.27 | 1.50 | 1.49 | 0.01 | 5.17 | 0.00 | / | 5.86 | 0.00 | / | Disease resistance protein RPM1 |
| augustus_masked-VaccDscaff13-processed-gene-80.3 | 3.83 | 0.87 | 2.14 | 4.08 | 2.83 | 0.53 | 3.05 | 3.29 | -0.11 | Disease resistance protein RPP13 |
| augustus_masked-VaccDscaff14-processed-gene-221.1 | 0.63 | 0.13 | 2.33 | 2.82 | 2.23 | 0.34 | 2.94 | 1.86 | 0.66 | Disease resistance protein RPP13 |
| augustus_masked-VaccDscaff28-processed-gene-85.6 | 0.68 | 0.46 | 0.55 | 2.23 | 3.63 | -0.70 | 3.44 | 3.44 | 0.00 | Disease resistance protein RPP13 |
| augustus_masked-VaccDscaff20-processed-gene-280.4 | 0.18 | 0.24 | -0.46 | 0.45 | 1.51 | -1.75 | 0.64 | 1.82 | -1.50 | Disease resistance protein RPP13 |
| augustus_masked-VaccDscaff25-processed-gene-267.7 | 0.14 | 0.06 | 1.13 | 0.85 | 6.55 | -2.95 | 0.85 | 6.13 | -2.85 | Disease resistance protein RPP13 |
| augustus_masked-VaccDscaff25-processed-gene-268.8 | 0.27 | 0.15 | 0.83 | 0.73 | 27.82 | -5.25 | 0.76 | 21.01 | -4.78 | Disease resistance protein RPP13 |
| augustus_masked-VaccDscaff2-processed-gene-257.0 | 0.22 | 1.30 | -2.57 | 0.69 | 6.61 | -3.26 | 0.65 | 6.15 | -3.25 | Disease resistance protein RPP13 |
| maker-VaccDscaff2-augustus-gene-304.22 | 1.07 | 0.44 | 1.29 | 5.78 | 4.19 | 0.46 | 10.98 | 5.14 | 1.09 | Disease resistance protein RPS2 |
| augustus_masked-VaccDscaff3-processed-gene-26.5 | 0.04 | 0.17 | -1.99 | 0.09 | 0.97 | -3.36 | 0.09 | 1.17 | -3.72 | Disease resistance protein RPS2 |
| augustus_masked-VaccDscaff3-processed-gene-50.1 | 1.81 | 1.68 | 0.11 | 3.97 | 6.97 | -0.81 | 4.45 | 6.34 | -0.51 | Disease resistance protein RPS2 |
| maker-VaccDscaff2-augustus-gene-405.43 | 0.47 | 0.37 | 0.35 | 0.71 | 3.78 | -2.41 | 1.68 | 3.83 | -1.19 | Disease resistance protein RPS2 |
| maker-VaccDscaff3-snap-gene-27.32 | 0.16 | 0.21 | -0.41 | 0.29 | 1.06 | -1.85 | 0.42 | 0.99 | -1.22 | Disease resistance protein RPS2 |
| augustus_masked-VaccDscaff28-processed-gene-88.0 | 1.04 | 0.55 | 0.91 | 3.11 | 4.75 | -0.61 | 3.19 | 9.35 | -1.55 | Disease resistance RPP13-like protein 3 |
| maker-VaccDscaff11-augustus-gene-236.26 | 5.05 | 4.12 | 0.29 | 10.67 | 8.81 | 0.28 | 15.55 | 6.23 | 1.32 | Regulatory protein NPR3 |
| maker-VaccDscaff24-augustus-gene-157.27 | 2.93 | 6.02 | -1.04 | 5.63 | 28.02 | -2.31 | 7.18 | 20.62 | -1.52 | Regulatory protein NPR3 |
| maker-VaccDscaff21-augustus-gene-334.26 | 0.97 | 1.50 | -0.62 | 0.24 | 0.29 | -0.29 | 0.13 | 0.17 | -0.43 | Regulatory protein NPR5 |
| maker-VaccDscaff26-augustus-gene-325.18 | 0.91 | 2.47 | -1.44 | 0.22 | 0.32 | -0.53 | 0.08 | 0.13 | -0.71 | Regulatory protein NPR5 |
| maker-VaccDscaff29-augustus-gene-312.19 | 0.68 | 1.48 | -1.11 | 0.24 | 0.48 | -1.01 | 0.03 | 0.19 | -2.85 | Regulatory protein NPR5 |
| augustus_masked-VaccDscaff24-processed-gene-58.1 | 17.16 | 5.18 | 1.73 | 14.39 | 19.14 | -0.41 | 31.11 | 29.73 | 0.07 | Pathogenesis-related genes transcriptional activator PTI6 |
| augustus_masked-VaccDscaff14-processed-gene-341.3 | 0.72 | 0.22 | 1.70 | 10.97 | 4.93 | 1.15 | 3.10 | 5.61 | -0.86 | Pathogenesis-related leaf protein 4 |
| maker-VaccDscaff14-augustus-gene-341.56 | 0.36 | 0.09 | 1.95 | 1.04 | 0.14 | 2.85 | 1.75 | 0.18 | 3.32 | Pathogenesis-related leaf protein 4 |
| maker-VaccDscaff3-snap-gene-17.37 | 0.06 | 2.89 | -5.70 | 1.25 | 2.16 | -0.79 | 0.26 | 2.29 | -3.16 | Pathogenesis-related leaf protein 4 |
| snap_masked-VaccDscaff2-processed-gene-394.25 | 0.03 | 3.15 | -6.88 | 1.39 | 1.57 | -0.18 | 0.29 | 2.07 | -2.85 | Pathogenesis-related leaf protein 4 |
| augustus_masked-VaccDscaff14-processed-gene-341.10 | 0.51 | 0.81 | -0.67 | 0.08 | 2.49 | -5.04 | 0.00 | 0.87 | / | Pathogenesis-related leaf protein 6 |
| augustus_masked-VaccDscaff14-processed-gene-342.1 | 11.33 | 5.59 | 1.02 | 0.64 | 4.78 | -2.89 | 0.00 | 0.70 | / | Pathogenesis-related protein 1 |
| maker-VaccDscaff3-snap-gene-17.35 | 1.48 | 27.49 | -4.21 | 1.68 | 11.65 | -2.79 | 2.97 | 3.25 | -0.13 | Pathogenesis-related protein 1A |
| augustus_masked-VaccDscaff14-processed-gene-341.7 | 0.00 | 0.00 | / | 3.62 | 3.45 | 0.07 | 2.08 | 1.89 | 0.14 | Pathogenesis-related protein PR-1 type |
| augustus_masked-VaccDscaff11-processed-gene-343.7 | 12.10 | 3.07 | 1.98 | 10.48 | 26.22 | -1.32 | 23.36 | 31.33 | -0.42 | Pathogenesis-related PTI6 |
| augustus_masked-VaccDscaff13-processed-gene-365.5 | 0.79 | 0.00 | / | 5.82 | 0.00 | / | 4.79 | 0.00 | / | BTB/POZ domain and ankyrin repeat-containing protein NPR1 |
| augustus_masked-VaccDscaff30-processed-gene-27.13 | 0.72 | 1.44 | -1.00 | 0.62 | 0.00 | / | 0.55 | 0.00 | / | BTB/POZ domain and ankyrin repeat-containing protein NPR1 |
| maker-VaccDscaff13-snap-gene-365.59 | 1.58 | 0.24 | 2.71 | 3.77 | 0.31 | 3.63 | 2.64 | 0.55 | 2.28 | BTB/POZ domain and ankyrin repeat-containing protein NPR1 |
| maker-VaccDscaff30-snap-gene-33.47 | 0.44 | 0.50 | -0.17 | 1.16 | 0.91 | 0.35 | 1.36 | 1.04 | 0.39 | BTB/POZ domain and ankyrin repeat-containing protein NPR1 |
| maker-VaccDscaff13-snap-gene-10.43 | 2.43 | 0.34 | 2.82 | 1.83 | 1.00 | 0.87 | 1.55 | 1.52 | 0.03 | Transcription factor TGA1 |
| maker-VaccDscaff30-augustus-gene-266.45 | 3.92 | 2.01 | 0.96 | 2.85 | 0.09 | 4.95 | 2.81 | 0.23 | 3.62 | Transcription factor TGA1 |
| maker-VaccDscaff37-augustus-gene-189.24 | 0.05 | 0.22 | -2.24 | 1.18 | 0.05 | 4.62 | 0.11 | 0.04 | 1.57 | Transcription factor TGA1 |
| maker-VaccDscaff37-snap-gene-220.62 | 0.10 | 0.44 | -2.20 | 2.63 | 0.10 | 4.71 | 0.27 | 0.09 | 1.68 | Transcription factor TGA1 |
| maker-VaccDscaff39-augustus-gene-97.23 | 0.18 | 0.69 | -1.94 | 3.87 | 0.14 | 4.75 | 0.30 | 0.08 | 1.96 | Transcription factor TGA1 |
| maker-VaccDscaff6-augustus-gene-323.25 | 0.03 | 0.33 | -3.50 | 1.92 | 0.19 | 3.30 | 0.22 | 0.22 | 0.00 | Transcription factor TGA1 |
| maker-VaccDscaff33-snap-gene-15.29 | 0.37 | 0.07 | 2.37 | 0.22 | 0.26 | -0.21 | 0.13 | 0.65 | -2.38 | Transcription factor TGA10 |
| maker-VaccDscaff1-snap-gene-277.30 | 10.70 | 15.82 | -0.56 | 8.20 | 4.74 | 0.79 | 7.04 | 4.79 | 0.56 | Transcription factor TGA2.1 |
| maker-VaccDscaff24-augustus-gene-350.40 | 2.21 | 7.28 | -1.72 | 4.99 | 5.84 | -0.23 | 4.27 | 4.20 | 0.03 | Transcription factor TGA2.1 |
| maker-VaccDscaff8-augustus-gene-169.11 | 5.41 | 6.60 | -0.29 | 4.43 | 2.10 | 1.08 | 3.97 | 1.48 | 1.43 | Transcription factor TGA2.1 |
| maker-VaccDscaff24-augustus-gene-350.41 | 2.91 | 10.68 | -1.87 | 5.32 | 5.92 | -0.15 | 4.35 | 4.48 | -0.04 | Transcription factor TGA2.2 |
| maker-VaccDscaff16-augustus-gene-318.35 | 0.28 | 0.11 | 1.38 | 1.19 | 0.48 | 1.30 | 1.03 | 0.47 | 1.14 | Transcription factor TGA3 |
| maker-VaccDscaff18-augustus-gene-83.29 | 1.41 | 1.12 | 0.33 | 4.27 | 1.56 | 1.46 | 4.12 | 1.67 | 1.30 | Transcription factor TGA3 |
| maker-VaccDscaff31-augustus-gene-259.18 | 0.35 | 0.33 | 0.07 | 1.45 | 0.42 | 1.80 | 1.31 | 0.60 | 1.14 | Transcription factor TGA3 |
| maker-VaccDscaff22-augustus-gene-238.25 | 1.22 | 6.01 | -2.31 | 0.33 | 0.02 | 3.87 | 0.28 | 0.09 | 1.63 | Transcription factor TGA9 |
| maker-VaccDscaff46-snap-gene-106.24 | 6.32 | 0.71 | 3.16 | 2.48 | 0.31 | 2.99 | 1.47 | 0.63 | 1.23 | Transcription factor TGA9 |
| snap_masked-VaccDscaff47-processed-gene-52.17 | 0.58 | 0.34 | 0.78 | 0.17 | 0.09 | 0.98 | 0.10 | 0.24 | -1.25 | Transcription factor TGA9 |
| maker-VaccDscaff14-snap-gene-340.48 | 0.47 | 0.12 | 1.97 | 0.75 | 2.62 | -1.80 | 1.01 | 2.25 | -1.16 | TMV resistance protein like |
| augustus_masked-VaccDscaff25-processed-gene-168.1 | 6.86 | 8.74 | -0.35 | 7.04 | 2.03 | 1.79 | 6.64 | 0.58 | 3.51 | PTI1-like tyrosine-protein kinase 3 |
| augustus_masked-VaccDscaff8-processed-gene-411.0 | 1.25 | 4.13 | -1.72 | 2.64 | 0.69 | 1.94 | 2.22 | 1.57 | 0.50 | Pto-interacting protein 1 |
| maker-VaccDscaff1-snap-gene-436.41 | 0.19 | 0.04 | 2.30 | 0.16 | 1.69 | -3.37 | 0.08 | 1.93 | -4.57 | Pto-interacting protein 1 |
| **Brassinosteroid-related transcripts** |  |  |  |  |  |  |  |  |  |  |
| augustus_masked-VaccDscaff17-processed-gene-121.4 | 0.00 | 0.25 | / | 0.00 | 2.89 | / | 0.00 | 2.39 | / | Brassinosteroid Insensitive 1-associated receptor kinase 1 |
| maker-VaccDscaff14-snap-gene-322.46 | 31.40 | 5.51 | 2.51 | 18.17 | 2.00 | 3.19 | 18.12 | 1.36 | 3.73 | Brassinosteroid Insensitive 1-associated receptor kinase 1 |
| maker-VaccDscaff17-augustus-gene-121.22 | 0.00 | 0.00 | / | 0.00 | 0.47 | -6.85 | 0.06 | 0.54 | -3.27 | Brassinosteroid Insensitive 1-associated receptor kinase 1 |
| maker-VaccDscaff17-augustus-gene-121.23 | 1.17 | 0.09 | 3.66 | 5.03 | 2.69 | 0.90 | 8.05 | 4.07 | 0.99 | Brassinosteroid Insensitive 1-associated receptor kinase 1 |
| maker-VaccDscaff17-snap-gene-122.34 | 0.09 | 1.99 | -4.44 | 0.23 | 2.64 | -3.53 | 0.45 | 2.33 | -2.38 | Brassinosteroid Insensitive 1-associated receptor kinase 1 |
| maker-VaccDscaff17-snap-gene-122.35 | 1.90 | 0.95 | 1.00 | 3.06 | 4.04 | -0.40 | 3.51 | 3.63 | -0.05 | Brassinosteroid Insensitive 1-associated receptor kinase 1 |
| maker-VaccDscaff27-augustus-gene-84.18 | 0.00 | 0.46 | / | 0.05 | 3.46 | -6.12 | 0.00 | 3.08 | / | Brassinosteroid Insensitive 1-associated receptor kinase 1 |
| maker-VaccDscaff27-augustus-gene-84.21 | 4.22 | 4.87 | -0.21 | 8.22 | 9.75 | -0.25 | 13.77 | 6.86 | 1.00 | Brassinosteroid Insensitive 1-associated receptor kinase 1 |
| maker-VaccDscaff27-snap-gene-84.25 | 0.04 | 0.42 | -3.22 | 0.04 | 2.14 | -5.69 | 0.20 | 2.48 | -3.64 | Brassinosteroid Insensitive 1-associated receptor kinase 1 |
| maker-VaccDscaff34-augustus-gene-232.36 | 12.68 | 0.54 | 4.54 | 8.39 | 1.34 | 2.65 | 5.60 | 1.35 | 2.05 | Brassinosteroid Insensitive 1-associated receptor kinase 1 |
| maker-VaccDscaff34-augustus-gene-232.37 | 21.99 | 3.00 | 2.88 | 14.18 | 4.13 | 1.78 | 13.12 | 4.60 | 1.51 | Brassinosteroid Insensitive 1-associated receptor kinase 1 |
| maker-VaccDscaff34-snap-gene-230.22 | 5.29 | 1.52 | 1.80 | 9.41 | 4.94 | 0.93 | 13.17 | 3.74 | 1.82 | Brassinosteroid Insensitive 1-associated receptor kinase 1 |
| maker-VaccDscaff45-augustus-gene-81.34 | 2.81 | 7.09 | -1.34 | 2.04 | 0.88 | 1.22 | 1.85 | 0.87 | 1.09 | Brassinosteroid Insensitive 1-associated receptor kinase 1 |
| maker-VaccDscaff45-augustus-gene-81.35 | 0.03 | 0.70 | -4.47 | 0.15 | 0.00 | / | 0.23 | 0.00 | / | Brassinosteroid Insensitive 1-associated receptor kinase 1 |
| maker-VaccDscaff45-snap-gene-80.23 | 7.32 | 6.43 | 0.19 | 4.66 | 1.53 | 1.61 | 4.52 | 2.24 | 1.01 | Brassinosteroid Insensitive 1-associated receptor kinase 1 |
| augustus_masked-VaccDscaff13-processed-gene-7.3 | 11.33 | 4.84 | 1.23 | 14.86 | 8.73 | 0.77 | 11.52 | 15.31 | -0.41 | Brassinosteroid Insensitive 1-associated receptor kinase 1 |
| maker-VaccDscaff31-snap-gene-332.45 | 4.37 | 3.25 | 0.43 | 0.96 | 0.97 | -0.02 | 0.50 | 1.18 | -1.23 | Serine/threonine-protein kinase BSK1 |
| maker-VaccDscaff1-augustus-gene-152.15 | 2.17 | 0.11 | 4.26 | 0.75 | 0.00 | / | 0.57 | 0.00 | / | Serine/threonine-protein kinase BSK3 |
| snap_masked-VaccDscaff24-processed-gene-76.15 | 2.40 | 10.02 | -2.06 | 3.08 | 17.49 | -2.50 | 1.28 | 19.45 | -3.93 | Serine/threonine-protein kinase BSK3 |
| snap_masked-VaccDscaff45-processed-gene-25.15 | 0.67 | 0.89 | -0.41 | 0.86 | 3.80 | -2.14 | 3.10 | 6.19 | -1.00 | Serine/threonine-protein kinase BSK3 |
| maker-VaccDscaff10-augustus-gene-258.25 | 0.04 | 1.39 | -5.01 | 0.08 | 0.03 | 1.34 | 0.07 | 0.11 | -0.66 | Serine/threonine-protein kinase BSK3 |
| maker-VaccDscaff17-augustus-gene-20.24 | 0.44 | 0.84 | -0.91 | 0.85 | 1.59 | -0.91 | 1.82 | 2.46 | -0.44 | Serine/threonine-protein kinase BSK3 |
| maker-VaccDscaff34-augustus-gene-253.27 | 0.27 | 0.59 | -1.14 | 0.32 | 0.61 | -0.94 | 1.26 | 0.78 | 0.68 | Serine/threonine-protein kinase BSK3 |
| maker-VaccDscaff8-augustus-gene-276.30 | 0.16 | 1.29 | -3.04 | 0.17 | 0.89 | -2.35 | 0.00 | 1.00 | / | Serine/threonine-protein kinase BSK3 |
| maker-VaccDscaff7-augustus-gene-33.28 | 5.30 | 4.17 | 0.34 | 16.12 | 8.09 | 0.99 | 10.94 | 8.58 | 0.35 | Brassinazole-Resistant 1 |
| maker-VaccDscaff16-augustus-gene-366.27 | 4.75 | 2.79 | 0.77 | 12.24 | 14.15 | -0.21 | 8.81 | 15.93 | -0.85 | Brassinazole-Resistant 1 |
| maker-VaccDscaff31-snap-gene-311.34 | 0.94 | 0.82 | 0.19 | 2.66 | 9.58 | -1.85 | 2.08 | 10.40 | -2.32 | Brassinazole-Resistant 1 |
| augustus_masked-VaccDscaff41-processed-gene-49.12 | 1.83 | 2.96 | -0.70 | 2.75 | 0.49 | 2.48 | 1.30 | 0.52 | 1.33 | BRI1 kinase inhibitor 1 |
| maker-VaccDscaff30-augustus-gene-220.32 | 29.14 | 19.81 | 0.56 | 35.41 | 44.58 | -0.33 | 35.67 | 68.29 | -0.94 | BES1/BZR1 homolog protein 2 |
| maker-VaccDscaff30-augustus-gene-222.17 | 9.45 | 8.37 | 0.17 | 10.61 | 15.87 | -0.58 | 11.14 | 25.28 | -1.18 | BES1/BZR1 homolog protein 2 |
| maker-VaccDscaff32-augustus-gene-212.35 | 6.97 | 24.95 | -1.84 | 9.10 | 4.59 | 0.99 | 8.30 | 7.30 | 0.18 | BES1/BZR1 homolog protein 2 |
| **Ca^2+^-related DEGs** |  |  |  |  |  |  |  |  |  |  |
| augustus_masked-VaccDscaff10-processed-gene-388.3 | 52.66 | 10.86 | 2.28 | 123.11 | 165.33 | -0.43 | 120.68 | 125.11 | -0.05 | Ca2+-binding protein 1 |
| augustus_masked-VaccDscaff5-processed-gene-12.15 | 82.02 | 23.87 | 1.78 | 188.78 | 497.49 | -1.40 | 193.08 | 378.89 | -0.97 | Ca2+-binding protein 1 |
| augustus_masked-VaccDscaff8-processed-gene-401.2 | 2.61 | 0.55 | 2.23 | 6.14 | 15.56 | -1.34 | 6.45 | 12.18 | -0.92 | Ca2+-binding protein 1 |
| augustus_masked-VaccDscaff45-processed-gene-67.3 | 1.40 | 1.92 | -0.46 | 0.97 | 7.47 | -2.95 | 1.19 | 5.05 | -2.09 | Calcium-binding protein CAST |
| augustus_masked-VaccDscaff34-processed-gene-292.7 | 5.36 | 0.62 | 3.11 | 2.45 | 4.94 | -1.01 | 3.20 | 4.34 | -0.44 | Calcium-binding protein CAST |
| augustus_masked-VaccDscaff29-processed-gene-81.0 | 6.14 | 9.14 | -0.57 | 20.37 | 55.01 | -1.43 | 23.31 | 66.64 | -1.52 | Calcium-binding protein CML13 |
| augustus_masked-VaccDscaff1226-processed-gene-0.1 | 125.48 | 5.49 | 4.51 | 67.82 | 85.35 | -0.33 | 74.93 | 52.62 | 0.51 | Calcium-binding protein CML18 |
| augustus_masked-VaccDscaff13-processed-gene-104.7 | 115.24 | 5.37 | 4.42 | 61.80 | 82.47 | -0.42 | 70.15 | 51.00 | 0.46 | Calcium-binding protein CML18 |
| augustus_masked-VaccDscaff35-processed-gene-236.4 | 14.13 | 22.06 | -0.64 | 52.19 | 72.75 | -0.48 | 57.18 | 57.55 | -0.01 | Calcium-binding protein CML18 |
| augustus_masked-VaccDscaff36-processed-gene-114.6 | 43.14 | 43.94 | -0.03 | 154.28 | 135.72 | 0.18 | 167.92 | 108.58 | 0.63 | Calcium-binding protein CML18 |
| augustus_masked-VaccDscaff3-processed-gene-384.6 | 0.24 | 0.13 | 0.86 | 0.82 | 1.63 | -0.99 | 1.38 | 1.45 | -0.07 | Calcium-binding protein CML18 |
| augustus_masked-VaccDscaff9-processed-gene-306.3 | 1.29 | 1.45 | -0.17 | 3.13 | 23.99 | -2.94 | 3.42 | 17.16 | -2.33 | Calcium-binding protein CML18 |
| augustus_masked-VaccDscaff30-processed-gene-310.2 | 3.34 | 0.30 | 3.47 | 9.41 | 8.64 | 0.12 | 11.00 | 3.24 | 1.76 | Calcium-binding protein CML19 |
| augustus_masked-VaccDscaff16-processed-gene-2.5 | 29.24 | 3.21 | 3.19 | 11.45 | 45.72 | -2.00 | 5.00 | 15.84 | -1.66 | Calcium-binding protein CML19 |
| augustus_masked-VaccDscaff18-processed-gene-381.7 | 2.38 | 0.45 | 2.41 | 2.11 | 6.66 | -1.66 | 0.95 | 5.84 | -2.62 | Calcium-binding protein CML19 |
| augustus_masked-VaccDscaff42-processed-gene-7.6 | 8.93 | 0.60 | 3.90 | 23.62 | 15.86 | 0.57 | 24.65 | 4.88 | 2.34 | Calcium-binding protein CML19 |
| augustus_masked-VaccDscaff7-processed-gene-407.2 | 42.25 | 1.05 | 5.34 | 18.47 | 46.72 | -1.34 | 7.72 | 20.14 | -1.38 | Calcium-binding protein CML19 |
| augustus_masked-VaccDscaff7-processed-gene-407.3 | 13.42 | 0.04 | 8.32 | 11.77 | 4.82 | 1.29 | 7.67 | 5.96 | 0.36 | Calcium-binding protein CML19 |
| snap_masked-VaccDscaff32-processed-gene-308.18 | 2.02 | 0.47 | 2.10 | 5.52 | 10.19 | -0.89 | 6.45 | 3.63 | 0.83 | Calcium-binding protein CML19 |
| maker-VaccDscaff3-augustus-gene-341.19 | 0.10 | 0.08 | 0.47 | 0.07 | 0.85 | -3.62 | 0.00 | 0.45 | / | Calcium-binding protein CML22 |
| augustus_masked-VaccDscaff44-processed-gene-179.3 | 2.89 | 8.41 | -1.54 | 1.09 | 1.37 | -0.33 | 1.70 | 1.39 | 0.29 | Calcium-binding protein CML25 |
| augustus_masked-VaccDscaff18-processed-gene-278.5 | 34.01 | 1.57 | 4.44 | 46.37 | 28.12 | 0.72 | 64.89 | 16.47 | 1.98 | Calcium-binding protein CML27 |
| augustus_masked-VaccDscaff31-processed-gene-25.5 | 86.78 | 10.66 | 3.03 | 129.23 | 99.23 | 0.38 | 144.67 | 67.74 | 1.09 | Calcium-binding protein CML27 |
| augustus_masked-VaccDscaff7-processed-gene-315.8 | 47.76 | 3.61 | 3.73 | 68.33 | 115.38 | -0.76 | 95.93 | 67.20 | 0.51 | Calcium-binding protein CML27 |
| snap_masked-VaccDscaff31-processed-gene-25.16 | 148.60 | 26.53 | 2.49 | 215.80 | 555.10 | -1.36 | 275.02 | 280.14 | -0.03 | Calcium-binding protein CML27 |
| snap_masked-VaccDscaff7-processed-gene-315.23 | 18.34 | 0.99 | 4.21 | 30.49 | 10.84 | 1.49 | 34.22 | 6.45 | 2.41 | Calcium-binding protein CML27 |
| maker-VaccDscaff1-augustus-gene-282.18 | 59.26 | 13.89 | 2.09 | 64.23 | 4.26 | 3.91 | 46.36 | 2.86 | 4.02 | Calcium-binding protein CML36 |
| snap_masked-VaccDscaff13-processed-gene-55.19 | 1.77 | 0.23 | 2.93 | 4.81 | 14.33 | -1.58 | 5.52 | 4.28 | 0.37 | Calcium-binding protein CML39 |
| augustus_masked-VaccDscaff32-processed-gene-217.2 | 13.14 | 19.13 | -0.54 | 9.93 | 1.63 | 2.60 | 1.61 | 1.38 | 0.22 | Calcium-binding protein CML41 |
| augustus_masked-VaccDscaff32-processed-gene-222.0 | 2.81 | 5.42 | -0.95 | 5.06 | 0.56 | 3.17 | 0.54 | 0.16 | 1.71 | Calcium-binding protein CML41 |
| maker-VaccDscaff14-snap-gene-157.32 | 0.83 | 0.00 | / | 0.71 | 3.21 | -2.18 | 1.42 | 1.59 | -0.17 | calcium-binding protein CML44 |
| maker-VaccDscaff29-augustus-gene-316.30 | 26.59 | 68.33 | -1.36 | 8.70 | 18.42 | -1.08 | 9.26 | 15.16 | -0.71 | Calcium-binding protein CML50 |
| maker-VaccDscaff36-augustus-gene-78.33 | 9.28 | 14.53 | -0.65 | 8.89 | 4.21 | 1.08 | 9.46 | 5.09 | 0.89 | Calcium-dependent protein kinase 10 |
| maker-VaccDscaff33-augustus-gene-77.27 | 13.28 | 0.19 | 6.12 | 9.36 | 9.61 | -0.04 | 8.36 | 3.68 | 1.19 | Calcium-dependent protein kinase 11 |
| maker-VaccDscaff26-augustus-gene-273.30 | 6.24 | 0.16 | 5.24 | 4.50 | 6.23 | -0.47 | 4.87 | 2.34 | 1.05 | Calcium-dependent protein kinase 17 |
| maker-VaccDscaff29-augustus-gene-262.33 | 19.78 | 0.19 | 6.71 | 13.54 | 10.13 | 0.42 | 13.33 | 3.36 | 1.99 | Calcium-dependent protein kinase 17 |
| maker-VaccDscaff12-snap-gene-44.37 | 0.21 | 0.08 | 1.43 | 2.18 | 0.57 | 1.94 | 2.47 | 1.07 | 1.21 | Calcium-dependent protein kinase 19 |
| maker-VaccDscaff32-augustus-gene-324.19 | 2.11 | 2.19 | -0.05 | 9.31 | 8.88 | 0.07 | 6.30 | 7.06 | -0.16 | Calcium-dependent protein kinase 28 |
| maker-VaccDscaff41-augustus-gene-274.23 | 5.55 | 3.21 | 0.79 | 23.53 | 27.05 | -0.20 | 47.34 | 26.80 | 0.82 | Calcium-dependent protein kinase 28 |
| maker-VaccDscaff12-augustus-gene-391.26 | 1.08 | 1.73 | -0.69 | 6.32 | 19.84 | -1.65 | 14.20 | 20.05 | -0.50 | Calcium-dependent protein kinase 28 |
| maker-VaccDscaff13-snap-gene-41.44 | 0.81 | 1.61 | -0.99 | 3.11 | 5.07 | -0.71 | 2.70 | 4.55 | -0.75 | Calcium-dependent protein kinase 28 |
| maker-VaccDscaff23-augustus-gene-5.20 | 2.79 | 2.12 | 0.40 | 15.13 | 20.49 | -0.44 | 33.32 | 20.70 | 0.69 | Calcium-dependent protein kinase 28 |
| maker-VaccDscaff53-augustus-gene-5.27 | 5.68 | 3.13 | 0.86 | 26.60 | 21.73 | 0.29 | 42.56 | 22.25 | 0.94 | Calcium-dependent protein kinase 28 |
| snap_masked-VaccDscaff30-processed-gene-297.14 | 2.30 | 1.20 | 0.93 | 11.16 | 5.28 | 1.08 | 8.11 | 3.66 | 1.15 | Calcium-dependent protein kinase 28 |
| maker-VaccDscaff35-augustus-gene-269.29 | 4.20 | 7.70 | -0.87 | 4.02 | 1.85 | 1.12 | 1.96 | 0.00 | / | Calcium-dependent protein kinase 30 |
| maker-VaccDscaff5-augustus-gene-275.26 | 4.93 | 2.72 | 0.86 | 4.10 | 11.19 | -1.45 | 4.15 | 10.59 | -1.35 | Calcium-dependent protein kinase 32 |
| maker-VaccDscaff24-augustus-gene-323.24 | 3.89 | 31.11 | -3.00 | 1.12 | 6.14 | -2.45 | 1.11 | 5.36 | -2.27 | Calcium-dependent protein kinase 7 |
| maker-VaccDscaff24-augustus-gene-354.24 | 14.48 | 31.49 | -1.12 | 48.05 | 95.89 | -1.00 | 46.50 | 114.60 | -1.30 | Calmodulin-1/11/16 |
| augustus_masked-VaccDscaff17-processed-gene-238.5 | 3.30 | 9.13 | -1.47 | 3.77 | 0.59 | 2.68 | 5.09 | 1.58 | 1.69 | Calmodulin-like protein 1 |
| augustus_masked-VaccDscaff206-processed-gene-1.8 | 3.79 | 11.62 | -1.62 | 4.86 | 0.86 | 2.50 | 6.29 | 3.59 | 0.81 | Calmodulin-like protein 1 |
| augustus_masked-VaccDscaff27-processed-gene-222.4 | 2.35 | 5.05 | -1.10 | 2.72 | 0.37 | 2.87 | 3.49 | 1.37 | 1.35 | Calmodulin-like protein 1 |
| augustus_masked-VaccDscaff34-processed-gene-135.9 | 8.07 | 12.01 | -0.57 | 8.61 | 1.69 | 2.35 | 12.61 | 5.86 | 1.11 | Calmodulin-like protein 1 |
| augustus_masked-VaccDscaff36-processed-gene-213.11 | 1.41 | 0.06 | 4.48 | 5.17 | 2.31 | 1.16 | 5.74 | 0.98 | 2.55 | Calmodulin-like protein 3 |
| snap_masked-VaccDscaff35-processed-gene-51.31 | 0.62 | 0.00 | / | 2.79 | 6.36 | -1.19 | 3.48 | 3.06 | 0.19 | Calmodulin-like protein 3 |
| snap_masked-VaccDscaff4-processed-gene-138.33 | 1.17 | 0.04 | 4.75 | 4.57 | 3.55 | 0.36 | 5.25 | 1.38 | 1.93 | Calmodulin-like protein 3 |
| augustus_masked-VaccDscaff3-processed-gene-273.6 | 2.69 | 0.36 | 2.92 | 9.50 | 20.91 | -1.14 | 9.90 | 14.30 | -0.53 | Calmodulin-like protein 5 |
| augustus_masked-VaccDscaff4-processed-gene-32.8 | 0.00 | 0.00 | / | 0.33 | 2.53 | -2.95 | 0.19 | 1.09 | -2.49 | Calmodulin-like protein 6 |
| **Other signal transduction factor-related DEGs** |  |  |  |  |  |  |  |  |  |  |
| maker-VaccDscaff2-snap-gene-152.19 | 0.18 | 0.24 | -0.44 | 0.20 | 1.13 | -2.47 | 0.35 | 1.20 | -1.77 | Cyclin-D3-1 |
| maker-VaccDscaff266-augustus-gene-0.64 | 0.08 | 0.58 | -2.90 | 0.33 | 1.47 | -2.15 | 0.52 | 2.91 | -2.49 | Cyclin-D3-2 |
| maker-VaccDscaff12-augustus-gene-100.18 | 0.99 | 0.92 | 0.10 | 2.68 | 3.57 | -0.42 | 4.04 | 6.84 | -0.76 | Cyclin-D3-3 |
| maker-VaccDscaff18-augustus-gene-6.29 | 1.82 | 0.65 | 1.48 | 1.53 | 4.00 | -1.39 | 1.97 | 4.03 | -1.03 | Cyclin-D3-3 |
| maker-VaccDscaff23-augustus-gene-312.33 | 0.43 | 1.47 | -1.76 | 0.52 | 4.08 | -2.98 | 2.35 | 8.75 | -1.90 | Cyclin-D3-3 |
| maker-VaccDscaff41-augustus-gene-39.28 | 1.54 | 2.06 | -0.42 | 3.93 | 10.58 | -1.43 | 5.94 | 19.68 | -1.73 | Cyclin-D3-3 |
| maker-VaccDscaff41-augustus-gene-40.31 | 1.72 | 3.42 | -0.99 | 3.90 | 7.21 | -0.89 | 5.05 | 13.13 | -1.38 | Cyclin-D3-3 |
| maker-VaccDscaff39-augustus-gene-0.29 | 1.56 | 0.21 | 2.92 | 2.46 | 0.36 | 2.76 | 1.97 | 0.07 | 4.74 | Xyloglucan endotransglucosylase/hydrolase 2 |
| maker-VaccDscaff160-augustus-gene-2.40 | 69.14 | 8.50 | 3.02 | 124.98 | 111.18 | 0.17 | 63.75 | 44.30 | 0.53 | Xyloglucan endotransglucosylase/hydrolase protein 23 |
| maker-VaccDscaff6-snap-gene-422.30 | 0.23 | 0.29 | -0.30 | 0.78 | 0.12 | 2.69 | 0.82 | 0.08 | 3.34 | Xyloglucan endotransglucosylase/hydrolase protein 23 |
| maker-VaccDscaff160-snap-gene-1.36 | 0.62 | 0.00 | / | 1.20 | 0.02 | 6.08 | 1.58 | 0.05 | 4.90 | Xyloglucan endotransglucosylase/hydrolase protein 23 |
| maker-VaccDscaff160-snap-gene-2.44 | 10.06 | 0.29 | 5.11 | 21.62 | 13.24 | 0.71 | 13.96 | 4.20 | 1.73 | Xyloglucan endotransglucosylase/hydrolase protein 23 |
| maker-VaccDscaff37-augustus-gene-274.38 | 0.03 | 15.99 | -8.85 | 0.00 | 3.31 | / | 0.00 | 2.26 | / | Xyloglucan endotransglucosylase/hydrolase protein 23 |
| maker-VaccDscaff37-augustus-gene-302.21 | 25.25 | 1.13 | 4.48 | 38.43 | 28.86 | 0.41 | 27.69 | 13.32 | 1.06 | Xyloglucan endotransglucosylase/hydrolase protein 23 |
| maker-VaccDscaff37-snap-gene-302.33 | 7.02 | 0.89 | 2.98 | 17.20 | 56.47 | -1.72 | 12.28 | 18.15 | -0.56 | Xyloglucan endotransglucosylase/hydrolase protein 23 |
| maker-VaccDscaff38-augustus-gene-100.23 | 2.40 | 1.85 | 0.38 | 0.73 | 0.00 | / | 0.88 | 0.00 | / | Xyloglucan endotransglucosylase/hydrolase protein 23 |
| maker-VaccDscaff39-snap-gene-0.31 | 2.69 | 0.00 | / | 10.59 | 2.53 | 2.06 | 9.53 | 1.29 | 2.89 | Xyloglucan endotransglucosylase/hydrolase protein 23 |
| maker-VaccDscaff6-augustus-gene-390.27 | 0.03 | 29.21 | -10.17 | 0.00 | 4.84 | / | 0.00 | 2.71 | / | Xyloglucan endotransglucosylase/hydrolase protein 23 |
| maker-VaccDscaff6-augustus-gene-421.32 | 6.72 | 0.13 | 5.71 | 10.41 | 2.19 | 2.25 | 5.78 | 0.70 | 3.04 | Xyloglucan endotransglucosylase/hydrolase protein 23 |
| maker-VaccDscaff6-snap-gene-421.41 | 0.52 | 0.00 | / | 12.09 | 0.08 | 7.22 | 11.86 | 0.00 | / | Xyloglucan endotransglucosylase/hydrolase protein 23 |
| maker-VaccDscaff8-augustus-gene-19.29 | 2.67 | 0.30 | 3.16 | 6.36 | 7.11 | -0.16 | 7.86 | 1.83 | 2.10 | Xyloglucan endotransglucosylase/hydrolase protein 23 |
| maker-VaccDscaff35-augustus-gene-62.23 | 0.19 | 2.09 | -3.45 | 0.81 | 0.18 | 2.16 | 0.50 | 0.16 | 1.65 | Mitogen-activated protein kinase homolog NTF4 |
| maker-VaccDscaff4-augustus-gene-152.27 | 1.50 | 2.19 | -0.54 | 2.01 | 0.34 | 2.55 | 2.33 | 0.23 | 3.34 | Mitogen-activated protein kinase homolog NTF4 |
| maker-VaccDscaff13-snap-gene-334.35 | 5.51 | 21.59 | -1.97 | 1.82 | 0.88 | 1.04 | 6.76 | 1.43 | 2.24 | Mitogen-activated protein kinase kinase 1 |
| maker-VaccDscaff30-snap-gene-65.32 | 2.57 | 10.57 | -2.04 | 0.88 | 2.48 | -1.50 | 2.90 | 2.08 | 0.48 | Mitogen-activated protein kinase kinase 1 |
| maker-VaccDscaff2-augustus-gene-356.30 | 0.01 | 1.21 | -7.53 | 0.00 | 0.90 | / | 0.01 | 0.97 | -6.03 | Mitogen-activated protein kinase kinase 5 |
| maker-VaccDscaff3-augustus-gene-50.38 | 1.13 | 0.62 | 0.86 | 1.17 | 2.52 | -1.10 | 1.07 | 2.81 | -1.39 | Mitogen-activated protein kinase kinase 5 |
| maker-VaccDscaff32-snap-gene-16.34 | 1.78 | 17.01 | -3.26 | 1.11 | 3.04 | -1.46 | 3.47 | 2.12 | 0.71 | Mitogen-activated protein kinase kinase 6 |
| augustus_masked-VaccDscaff25-processed-gene-229.8 | 2.34 | 4.72 | -1.01 | 2.58 | 0.80 | 1.69 | 3.10 | 0.90 | 1.78 | Mitogen-activated protein kinase kinase kinase 1 |
| maker-VaccDscaff13-snap-gene-348.27 | 2.05 | 0.37 | 2.46 | 2.98 | 1.24 | 1.27 | 3.76 | 1.26 | 1.57 | Mitogen-activated protein kinase kinase kinase 1 |
| maker-VaccDscaff42-snap-gene-247.27 | 2.15 | 0.53 | 2.02 | 3.50 | 1.79 | 0.97 | 4.41 | 1.72 | 1.36 | Mitogen-activated protein kinase kinase kinase 1 |
| maker-VaccDscaff43-snap-gene-127.52 | 0.30 | 1.25 | -2.06 | 0.11 | 0.12 | -0.03 | 0.21 | 0.11 | 0.92 | Voltage dependent potassium channe l |
| maker-VaccDscaff47-snap-gene-74.42 | 0.77 | 0.69 | 0.15 | 0.28 | 0.18 | 0.68 | 0.14 | 0.14 | -0.02 | Voltage dependent potassium channe l |
| maker-VaccDscaff22-snap-gene-259.46 | 0.30 | 1.62 | -2.42 | 0.12 | 0.11 | 0.18 | 0.18 | 0.11 | 0.67 | Voltage dependent potassium channe l |
| maker-VaccDscaff78-augustus-gene-6.60 | 8.57 | 7.79 | 0.14 | 6.09 | 0.06 | 6.68 | 5.90 | 0.04 | 7.36 | Transcription factor WRKY 2 |
| maker-VaccDscaff12-snap-gene-296.36 | 1.98 | 0.12 | 4.07 | 3.89 | 1.61 | 1.27 | 9.83 | 0.53 | 4.22 | Transcription factor WRKY22 |
| maker-VaccDscaff22-augustus-gene-15.21 | 3.81 | 1.86 | 1.04 | 2.74 | 8.26 | -1.59 | 10.34 | 4.56 | 1.18 | Transcription factor WRKY22 |
| maker-VaccDscaff23-augustus-gene-96.33 | 0.37 | 0.04 | 3.11 | 0.73 | 5.02 | -2.78 | 1.64 | 1.96 | -0.26 | Transcription factor WRKY22 |
| maker-VaccDscaff45-augustus-gene-55.32 | 1.49 | 0.07 | 4.44 | 0.88 | 5.98 | -2.76 | 1.75 | 4.14 | -1.24 | Transcription factor WRKY24 |
| maker-VaccDscaff19-augustus-gene-368.29 | 20.52 | 3.14 | 2.71 | 32.49 | 60.64 | -0.90 | 24.96 | 36.34 | -0.54 | Transcription factor WRKY24 |
| maker-VaccDscaff22-augustus-gene-298.44 | 29.04 | 3.03 | 3.26 | 38.67 | 0.75 | 5.68 | 13.78 | 0.28 | 5.62 | Transcription factor WRKY24 |
| maker-VaccDscaff27-augustus-gene-12.38 | 2.88 | 0.08 | 5.22 | 2.15 | 1.20 | 0.84 | 3.79 | 0.50 | 2.91 | Transcription factor WRKY24 |
| maker-VaccDscaff34-augustus-gene-280.25 | 0.62 | 0.06 | 3.34 | 0.29 | 1.18 | -2.01 | 0.50 | 0.77 | -0.62 | Transcription factor WRKY24 |
| maker-VaccDscaff43-augustus-gene-79.16 | 20.42 | 1.55 | 3.72 | 25.59 | 7.82 | 1.71 | 10.19 | 3.57 | 1.51 | Transcription factor WRKY24 |
| maker-VaccDscaff47-augustus-gene-120.37 | 13.63 | 1.51 | 3.18 | 17.31 | 33.61 | -0.96 | 7.41 | 16.05 | -1.12 | Transcription factor WRKY24 |
| maker-VaccDscaff24-snap-gene-64.33 | 6.66 | 4.43 | 0.59 | 10.91 | 80.62 | -2.88 | 8.48 | 50.28 | -2.57 | Transcription factor WRKY24 |
| maker-VaccDscaff19-snap-gene-10.40 | 2.02 | 0.44 | 2.21 | 2.95 | 1.41 | 1.06 | 2.73 | 1.27 | 1.11 | Cyclic nucleotide-gated ion channel 1 |
| maker-VaccDscaff20-snap-gene-11.41 | 10.29 | 0.68 | 3.93 | 22.99 | 2.27 | 3.34 | 20.50 | 1.73 | 3.57 | Cyclic nucleotide-gated ion channel 1 |
| maker-VaccDscaff19-augustus-gene-56.71 | 0.58 | 10.26 | -4.15 | 1.11 | 0.03 | 5.39 | 2.26 | 0.00 | / | Cyclic nucleotide-gated ion channel 14 |
| maker-VaccDscaff39-snap-gene-6.40 | 6.00 | 4.03 | 0.57 | 7.29 | 0.75 | 3.29 | 7.04 | 0.68 | 3.36 | Cyclic nucleotide-gated ion channel 17 |
| maker-VaccDscaff421-snap-gene-0.23 | 0.93 | 0.45 | 1.04 | 4.27 | 3.90 | 0.13 | 3.90 | 3.01 | 0.37 | Cyclic nucleotide-gated ion channel 19 |
| augustus_masked-VaccDscaff61-processed-gene-0.4 | 10.47 | 25.38 | -1.28 | 12.31 | 8.06 | 0.61 | 12.25 | 8.99 | 0.45 | Cyclic nucleotide-gated ion channel 5 |
| maker-VaccDscaff21-snap-gene-77.27 | 0.01 | 3.42 | -9.06 | 0.01 | 0.42 | -4.91 | 0.00 | 0.65 | / | Cysteine and histidine-rich domain-containing protein RAR1 |
| maker-VaccDscaff17-augustus-gene-337.44 | 4.77 | 5.60 | -0.23 | 6.18 | 0.28 | 4.48 | 5.74 | 0.92 | 2.64 | Glycerol kinase |
| maker-VaccDscaff27-augustus-gene-300.32 | 5.64 | 6.20 | -0.14 | 6.46 | 0.00 | / | 6.53 | 1.16 | 2.49 | Glycerol kinase |
| augustus_masked-VaccDscaff83-processed-gene-1.3 | 102.66 | 167.99 | -0.71 | 96.76 | 43.63 | 1.15 | 77.36 | 90.07 | -0.22 | Heat shock cognate protein 80 |
| maker-VaccDscaff33-augustus-gene-283.25 | 1.31 | 0.65 | 1.02 | 2.30 | 4.59 | -1.00 | 1.79 | 80.07 | -5.49 | Heat shock protein 83 |
| maker-VaccDscaff39-augustus-gene-20.40 | 0.05 | 1.25 | -4.65 | 0.00 | 0.00 | / | 0.00 | 0.00 | / | Heat shock protein 83 |
| maker-VaccDscaff2-augustus-gene-307.40 | 0.67 | 0.94 | -0.50 | 0.83 | 0.07 | 3.54 | 0.84 | 0.10 | 3.05 | Hypothetical protein Pyn_39352 |
| maker-VaccDscaff15-augustus-gene-78.28 | 9.93 | 7.51 | 0.40 | 0.80 | 2.33 | -1.55 | 0.63 | 0.89 | -0.49 | LRR receptor-like serine/threonine-protein kinase FLS2 |
| maker-VaccDscaff24-augustus-gene-346.35 | 16.52 | 14.67 | 0.17 | 1.18 | 2.63 | -1.16 | 1.03 | 1.20 | -0.21 | LRR receptor-like serine/threonine-protein kinase FLS2 |
| augustus_masked-VaccDscaff5-processed-gene-331.4 | 2.30 | 1.14 | 1.01 | 0.26 | 0.16 | 0.65 | 0.72 | 0.18 | 2.01 | LysM domain receptor-like kinase 3 |
| maker-VaccDscaff1-snap-gene-351.55 | 6.10 | 2.25 | 1.44 | 0.67 | 0.34 | 1.00 | 0.68 | 0.15 | 2.22 | LysM domain receptor-like kinase 3 |
| maker-VaccDscaff1-snap-gene-352.45 | 2.97 | 1.60 | 0.89 | 6.88 | 8.33 | -0.28 | 16.00 | 7.94 | 1.01 | LysM domain receptor-like kinase 3 |
| maker-VaccDscaff8-snap-gene-104.38 | 1.60 | 2.74 | -0.78 | 2.62 | 14.24 | -2.44 | 4.36 | 11.82 | -1.44 | LysM domain receptor-like kinase 3 |
| maker-VaccDscaff34-snap-gene-59.36 | 20.95 | 2.78 | 2.92 | 23.07 | 20.09 | 0.20 | 17.05 | 11.39 | 0.58 | NADPH oxidase A |
| maker-VaccDscaff50-augustus-gene-20.17 | 21.17 | 1.91 | 3.47 | 25.76 | 27.42 | -0.09 | 20.37 | 14.09 | 0.53 | NADPH oxidase A |
| maker-VaccDscaff21-snap-gene-87.47 | 10.45 | 9.59 | 0.12 | 2.48 | 2.38 | 0.06 | 2.60 | 2.22 | 0.23 | NO-associated protein 1 |
| maker-VaccDscaff33-augustus-gene-252.45 | 31.18 | 20.77 | 0.59 | 6.37 | 6.42 | -0.01 | 6.85 | 4.65 | 0.56 | NO-associated protein 1 |
| maker-VaccDscaff2-snap-gene-405.55 | 0.61 | 0.03 | 4.34 | 0.75 | 1.78 | -1.25 | 1.15 | 1.19 | -0.05 | Plant basic secretory protein BSP |
| maker-VaccDscaff18-augustus-gene-8.29 | 5.90 | 5.44 | 0.12 | 2.20 | 0.62 | 1.82 | 3.16 | 1.21 | 1.38 | Probable serine/threonine-protein kinase |
| maker-VaccDscaff48-snap-gene-95.48 | 0.38 | 0.42 | -0.14 | 1.88 | 3.62 | -0.94 | 2.05 | 2.91 | -0.51 | Protein SGT1 B like |
| maker-VaccDscaff19-snap-gene-15.45 | 1.58 | 0.79 | 0.99 | 6.08 | 4.09 | 0.57 | 5.68 | 3.33 | 0.77 | Protein SGT1 homolog |
| augustus_masked-VaccDscaff24-processed-gene-132.3 | 0.03 | 0.00 | / | 0.14 | 0.46 | -1.67 | 0.08 | 0.45 | -2.46 | Receptor kinase-like protein Xa21 |
| maker-VaccDscaff24-augustus-gene-131.17 | 0.05 | 0.03 | 1.05 | 0.13 | 1.72 | -3.70 | 0.29 | 1.01 | -1.81 | Receptor kinase-like protein Xa21 |
| augustus_masked-VaccDscaff11-processed-gene-71.4 | 9.41 | 5.42 | 0.80 | 1.11 | 1.44 | -0.38 | 0.66 | 0.42 | 0.63 | receptor-like serine/threonine-protein kinase FLS2 |
| maker-VaccDscaff20-snap-gene-338.29 | 0.18 | 0.08 | 1.20 | 2.36 | 0.81 | 1.54 | 1.70 | 0.53 | 1.68 | Respiratory burst oxidase homolog protein A |
| maker-VaccDscaff28-augustus-gene-35.27 | 0.27 | 0.33 | -0.27 | 5.46 | 2.50 | 1.13 | 2.85 | 2.33 | 0.29 | Respiratory burst oxidase homolog protein A |
| maker-VaccDscaff44-augustus-gene-27.21 | 0.15 | 0.10 | 0.63 | 2.26 | 1.40 | 0.69 | 1.52 | 1.24 | 0.30 | Respiratory burst oxidase homolog protein A |
| maker-VaccDscaff17-augustus-gene-321.35 | 19.54 | 1.15 | 4.09 | 20.07 | 16.29 | 0.30 | 14.92 | 9.24 | 0.69 | Respiratory burst oxidase homolog protein C |
| maker-VaccDscaff4-augustus-gene-336.22 | 2.04 | 0.12 | 4.07 | 1.05 | 0.65 | 0.70 | 1.34 | 0.72 | 0.90 | Ribonuclease H2 subunit A |
| augustus_masked-VaccDscaff15-processed-gene-346.8 | 1.69 | 3.20 | -0.92 | 0.05 | 0.17 | -1.83 | 0.03 | 0.17 | -2.32 | RPM1-interacting protein |
| augustus_masked-VaccDscaff24-processed-gene-47.2 | 0.93 | 9.60 | -3.37 | 0.80 | 2.56 | -1.67 | 0.59 | 2.17 | -1.88 | RPM1-interacting protein |
| maker-VaccDscaff11-snap-gene-354.58 | 9.88 | 17.30 | -0.81 | 14.03 | 0.36 | 5.29 | 11.50 | 0.30 | 5.25 | RPM1-interacting protein |
| maker-VaccDscaff25-augustus-gene-66.20 | 0.10 | 2.50 | -4.70 | 0.18 | 0.83 | -2.19 | 0.20 | 0.72 | -1.81 | RPM1-interacting protein 4 |
| maker-VaccDscaff23-snap-gene-172.28 | 0.44 | 0.54 | -0.30 | 0.44 | 2.35 | -2.43 | 0.45 | 1.78 | -1.97 | Serine/threonine-protein kinase |
| maker-VaccDscaff13-augustus-gene-315.41 | 4.16 | 0.53 | 2.98 | 7.40 | 5.27 | 0.49 | 4.20 | 5.26 | -0.32 | Serine/threonine-protein kinase |
| maker-VaccDscaff14-augustus-gene-390.37 | 2.83 | 0.67 | 2.07 | 3.70 | 0.84 | 2.13 | 6.98 | 1.25 | 2.48 | Serine/threonine-protein kinase |
| maker-VaccDscaff16-augustus-gene-391.46 | 4.42 | 2.48 | 0.83 | 1.39 | 0.64 | 1.12 | 1.46 | 0.71 | 1.03 | Serine/threonine-protein kinase |
| maker-VaccDscaff19-augustus-gene-21.26 | 0.00 | 3.65 | / | 0.00 | 0.00 | / | 0.00 | 0.00 | / | Serine/threonine-protein kinase |
| maker-VaccDscaff23-augustus-gene-357.25 | 18.44 | 37.12 | -1.01 | 7.21 | 6.42 | 0.17 | 8.07 | 5.94 | 0.44 | Serine/threonine-protein kinase |
| maker-VaccDscaff28-augustus-gene-50.29 | 4.95 | 12.62 | -1.35 | 4.81 | 0.09 | 5.69 | 5.23 | 0.12 | 5.44 | Serine/threonine-protein kinase |
| maker-VaccDscaff7-snap-gene-5.36 | 3.75 | 2.24 | 0.75 | 1.05 | 0.58 | 0.85 | 0.73 | 0.09 | 3.09 | Serine/threonine-protein kinase |
| snap_masked-VaccDscaff102-processed-gene-4.12 | 0.44 | 0.28 | 0.65 | 0.39 | 1.13 | -1.55 | 0.56 | 1.18 | -1.07 | Serine/threonine-protein kinase PBS1 |
| maker-VaccDscaff16-augustus-gene-317.35 | 3.04 | 0.38 | 3.02 | 2.83 | 1.47 | 0.95 | 3.14 | 1.13 | 1.47 | Somatic embryogenesis receptor kinase 1 |
| maker-VaccDscaff40-snap-gene-189.38 | 0.35 | 0.00 | / | 0.77 | 2.35 | -1.61 | 2.77 | 0.92 | 1.59 | Transcription factor 22 |
| maker-VaccDscaff41-augustus-gene-176.26 | 0.26 | 0.06 | 2.20 | 0.86 | 1.76 | -1.04 | 1.67 | 0.61 | 1.46 | Transcription factor 22 |
| maker-VaccDscaff1457-augustus-gene-0.4 | 0.00 | 0.00 | / | 0.28 | 1.00 | -1.81 | 1.12 | 1.49 | -0.41 | Transcription factor bHLH14 |
| maker-VaccDscaff66-augustus-gene-0.18 | 0.27 | 0.01 | 4.70 | 91.93 | 19.97 | 2.20 | 115.58 | 33.75 | 1.78 | Transcription factor bHLH14 |
| maker-VaccDscaff6-augustus-gene-252.32 | 0.07 | 0.01 | 2.73 | 26.91 | 9.70 | 1.47 | 35.20 | 14.61 | 1.27 | Transcription factor bHLH14 |
| maker-VaccDscaff108-augustus-gene-1.26 | 0.04 | 0.01 | 2.14 | 33.54 | 24.26 | 0.47 | 40.49 | 41.19 | -0.02 | Transcription factor bHLH14 |
| maker-VaccDscaff67-augustus-gene-7.67 | 0.20 | 0.02 | 3.25 | 59.21 | 30.08 | 0.98 | 73.74 | 45.62 | 0.69 | Transcription factor MYC2 |

**Table S6.** The expression levels and foldchanges of key homologous regulatory genes involved in *Solanum lycopersicum* fruit weight/size variation.

| **Gene_ID** | **FPKM** | | **Log_2_(ratio)** | **FPKM** | | **Log_2_(ratio)** | **FPKM** | | **Log_2_(ratio)** |
| --- | --- | --- | --- | --- | --- | --- | --- | --- | --- |
|  | **BrSO** | **ONS0** | **(BrS0/ONS0)** | **BrS1** | **ONS1** | **(BrS1/ONS1)** | **BrS2** | **ONS2** | **(BrS2/ONS2)** |
| ***VcFW2.2*** |  |  |  |  |  |  |  |  |  |
| VaccDscaff5-processed-gene-169.8 | 23.22 | 29.49 | -0.34 | 21.17 | 35.38 | -0.74 | 21.52 | 32.55 | -0.60 |
| VaccDscaff8-augustus-gene-244.19 | 39.53 | 47.30 | -0.26 | 38.31 | 34.70 | 0.14 | 40.63 | 32.61 | 0.32 |
| VaccDscaff10-snap-gene-228.26 | 0.51 | 31.96 | -5.98 | 0.44 | 8.43 | -4.25 | 0.51 | 7.08 | -3.79 |
| VaccDscaff1-snap-gene-180.23 | 56.11 | 34.45 | 0.70 | 80.89 | 0.71 | 6.83 | 71.44 | 0.71 | 6.66 |
| ***VcFW11.3*** |  |  |  |  |  |  |  |  |  |
| VaccDscaff43-snap-gene-145.14 | 0.02 | 0.22 | -3.68 | 0.11 | 0.23 | -1.03 | 0.13 | 1.40 | -3.41 |
| VaccDscaff46-snap-gene-114.13 | 0.36 | 0.18 | 0.97 | 0.50 | 0.50 | 0.00 | 0.70 | 0.99 | -0.51 |
| VaccDscaff47-processed-gene-42.1 | 0.26 | 0.14 | 0.93 | 0.46 | 0.72 | -0.65 | 0.48 | 1.40 | -1.53 |
| VaccDscaff22-processed-gene-232.6 | 0.39 | 0.31 | 0.34 | 0.24 | 0.20 | 0.23 | 0.49 | 0.70 | -0.51 |
| ***VcFW3.2*** |  |  |  |  |  |  |  |  |  |
| VaccDscaff4-augustus-gene-348.22 | 0.04 | 0.00 | / | 0.03 | 0.35 | -3.63 | 0.01 | 0.58 | -5.85 |
| VaccDscaff36-augustus-gene-94.16 | 0.52 | 0.07 | 2.81 | 0.39 | 0.82 | -1.05 | 0.52 | 1.06 | -1.02 |
| VaccDscaff42-augustus-gene-147.21 | 0.04 | 0.01 | 2.00 | 0.01 | 0.06 | -2.77 | 0.02 | 0.04 | -1.14 |
| VaccDscaff9-augustus-gene-324.20 | 0.38 | 0.13 | 1.57 | 0.26 | 0.48 | -0.91 | 0.49 | 0.60 | -0.31 |
| VaccDscaff30-augustus-gene-169.20 | 0.15 | 0.09 | 0.74 | 0.03 | 0.12 | -2.16 | 0.12 | 0.14 | -0.20 |
| VaccDscaff13-augustus-gene-231.18 | 0.02 | 0.02 | -0.62 | 0.01 | 0.09 | -3.48 | 0.00 | 0.22 | / |
| VaccDscaff32-augustus-gene-151.27 | 0.02 | 0.00 | / | 0.03 | 0.02 | 0.04 | 0.01 | 0.00 | / |
| ***VcENO*** |  |  |  |  |  |  |  |  |  |
| VaccDscaff17-processed-gene-336.36 | 0.00 | 0.02 | / | 0.00 | 0.00 | / | 0.00 | 0.00 | / |
| VaccDscaff50-processed-gene-25.22 | 0.00 | 0.00 | / | 0.00 | 0.00 | / | 0.00 | 0.00 | / |
| VaccDscaff27-processed-gene-296.10 | 0.00 | 0.00 | / | 0.00 | 0.00 | / | 0.00 | 0.00 | / |

**Table S7.** Primers used in this study.

| **Gene name** | **Primer sequences (5'-3')** | **Predicted**  **Lenth (nt)** | **Tm (℃)** |  |
| --- | --- | --- | --- | --- |
| *Probable indole-3-pyruvate monooxygenase*  (VaccDscaff12-processed-gene-345.9-mRNA-1) | F：TTGCGTAGATGGGACATGC  R：AGTGAGGATGGAGCGAGAG | 149 | 57 |  |
| *Protein PIN-like 1*  [(VaccDscaff23-snap-gene-55.28-mRNA-1](https://www.vaccinium.org/feature/VaccDscaff23-snap-gene-55.28-mRNA-1)) | F：GCAGTGATGGTAAAGAGAAACAAG  R：AATGCCACAATAAACAGATCAAGG | 130 | 58 |  |
| *Protein PIN-like 7*  (VaccDscaff32-snap-gene-331.35-mRNA-1) | F：TGATGGCACGACTATAGCAAC  R：GAAGCTCGGCATTGAAATATAAGG | 140 | 58 |  |
| *Auxin-responsive protein IAA26*  (maker-VaccDscaff17-augustus-gene-358.28) | F：CCACTACAACTACATGCTATACGG  R：GGGAATATTCAGAAGGGTGCC | 114 | 52 |  |
| *IAA-amino acid hydrolase ILR1*  (VaccDscaff26-snap-gene-262.43-mRNA-1) | F：CCACTACAACTACATGCTATACGG  R：GGGAATATTCAGAAGGGTGCC | 114 | 52 |  |
| *Zeatin O-glucosyltransferase*  (VaccDscaff10-snap-gene-142.39-mRNA-1) | F：GACTTGCACTAAACATCGACAAC  R：CTTCTGCTTCATCTCGTCCG | 133 | 58 |  |
| *Transcription factor PIF3*  (maker-VaccDscaff17-snap-gene-157.22) | F：ACCAAATTCCCCACTCACC  R：GTGGATGACGATAACCCTTCTC | 117 | 57 |  |
| *Gibberellin-regulated protein 11*  ([VaccDscaff4-augustus-gene-308.25-mRNA-1](https://www.vaccinium.org/feature/VaccDscaff4-augustus-gene-308.25-mRNA-1)) | F：ACACGACCACAAGTACATCAC  R：TCTTGATATAGACCGTCCCCTC | 150 | 58 |  |
| *1-aminocyclopropane-1-carboxylate*  (VaccDscaff6-augustus-gene-92.23-mRNA-1) | F：AATGGACAGCTCGGATGC  R：GCAGTCTAGGAAGTTAGGTATAAGAG | 285 | 61 |  |
| *Ethylene-insensitive protein 2*  (snap_masked-VaccDscaff27-processed-gene-53.13) | F：CCCCGTCTTTTGTATCCCAG  R：GGCATGTCGTTGTCTTATGTTG | 142 | 58 |  |
| *1-aminocyclopropane-1-carboxylate*  (VaccDscaff5-snap-gene-79.27-mRNA-1) | F：CTTGACTGCTCGTTGAATTGG  R：TGGGAGTTTGGGTGTTGG | 148 | 57 |  |
| *Protein TIFY 10A*  (maker-VaccDscaff26-snap-gene-12.21) | F：CCAAGACCTGTACAAGCTCAG  R：CTTTTCGGTCGCAGAAAGTTG | 143 | 58 |  |
| *Topless-related protein 1*  (VaccDscaff35-augustus-gene-295.15-mRNA-1) | F：GATACAAAATCTGCAAGGGCG  R：AGCTTTGGTTAATGAGAGTCCG | 133 | 57 |  |
| *Protein C2-DOMAIN ABA-RELATED 4*  (VaccDscaff36-snap-gene-115.30-mRNA-1) | F：GAGAAATGATCGGTACCCTCC  R：ACCTGTTACCAAAAGAGCAGTG | 150 | 57 |  |
| *Cytokinin riboside 5'-monophosphate phosphoribohydrolase*  (VaccDscaff3-augustus-gene-415.21-mRNA-1) | F：CGATTGACTTCCTCTCACCTG  R：ATCGTCTCTTTTCTCCAACCC | 117 | 57 |  |

**Fig. S1.** Equatorial sections of *V. corymbosum* 'O'Neal' and 'Bluerain' hypanthium/fruit at different developmental stages.

**
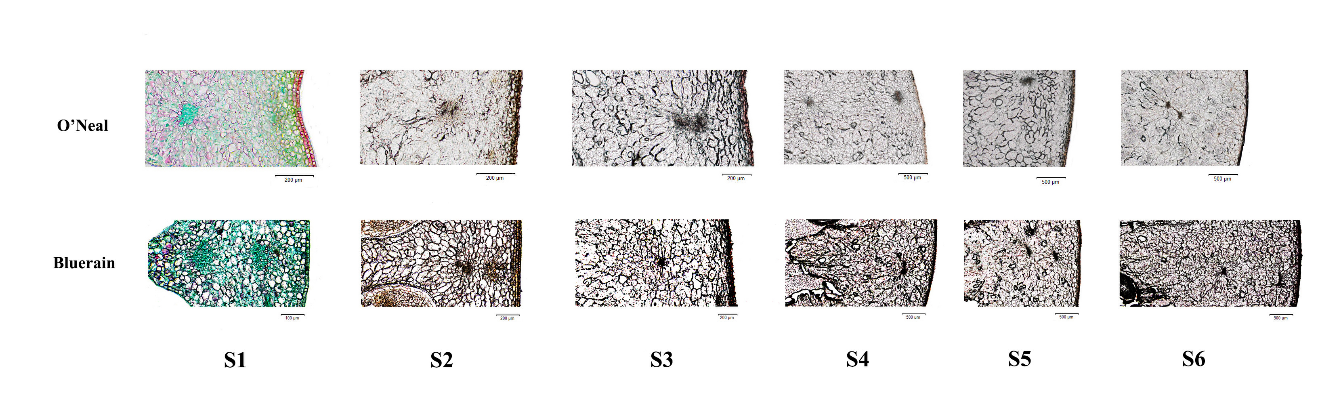

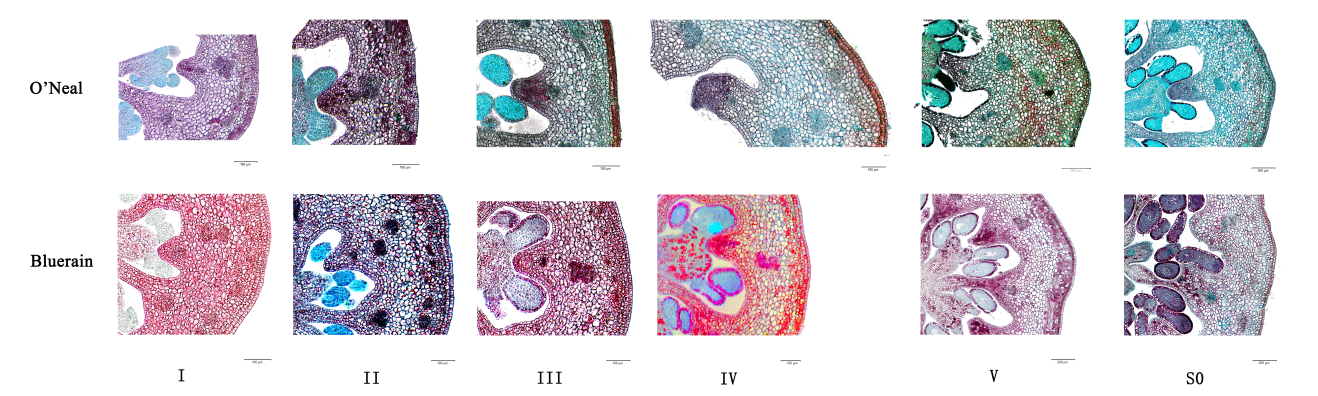
**

**Fig. S2****.** Total area of outer mesocarp, middle mesocarp and inner mesocarp during *V. corymbosum* 'O'Neal' and 'Bluerain' flower bud and fruit development.

**Fig. S3**. Total cell number and fruit area increase patterns during *V. corymbosum* 'O'Neal' and 'Bluerain' flower bud and fruit development.

**Fig. S4.** Relative expression levels of 15 randomly selected DEGs determined by qPCR.

**Fig. S5.** Spearman correlation coefficient of transcriptomic profiles of early *V. corymbosum* 'O'Neal' and 'Bluerain' fruit development.

**
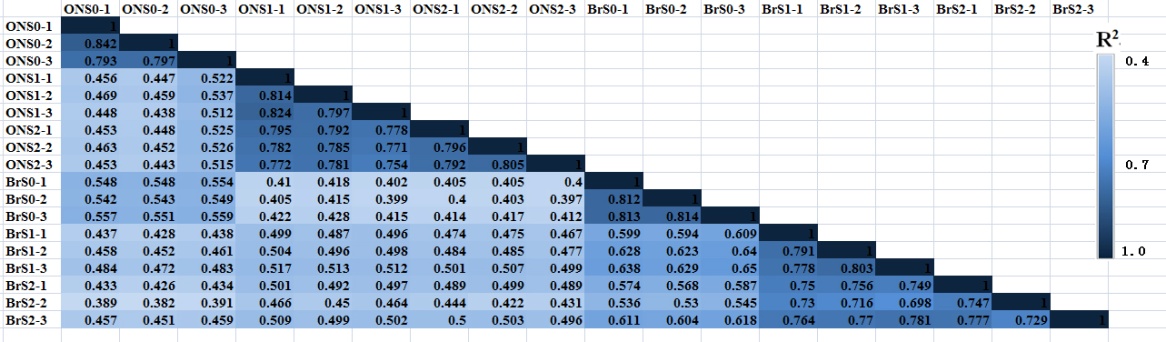
**

**Fig. S6.** KEGG function classification of DEGs associated with early *V. corymbosum* 'O'Neal' and 'Bluerain' fruit development.


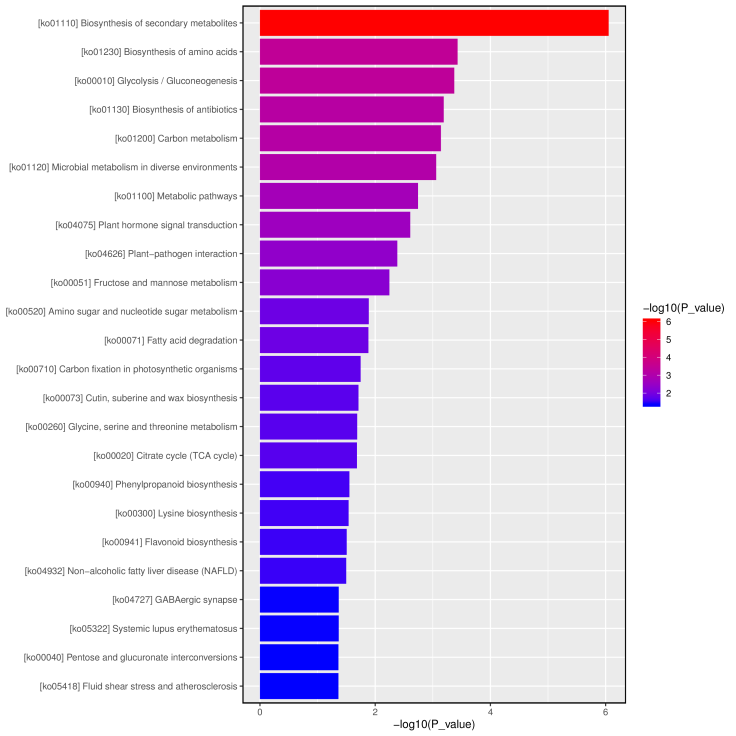


BrS0 vs ONS0

**
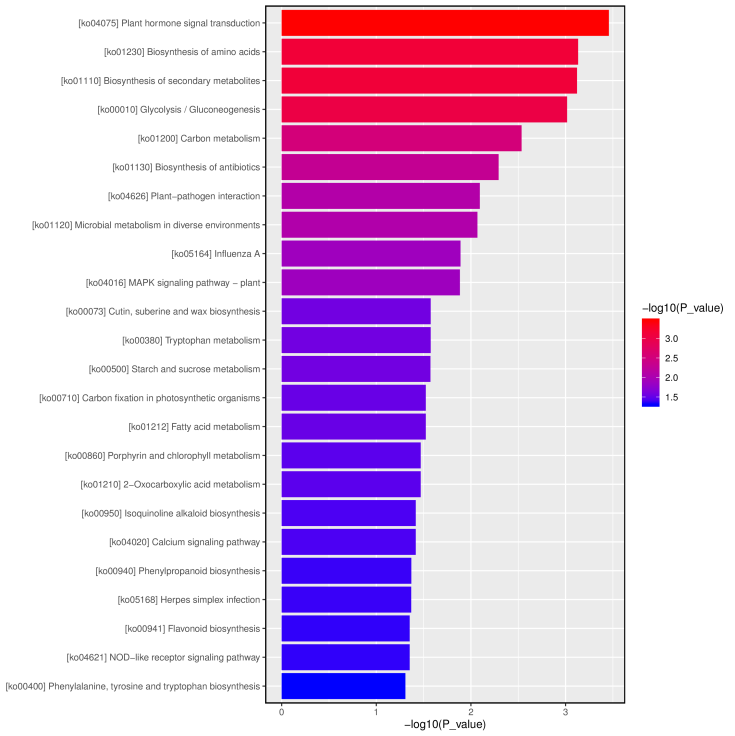

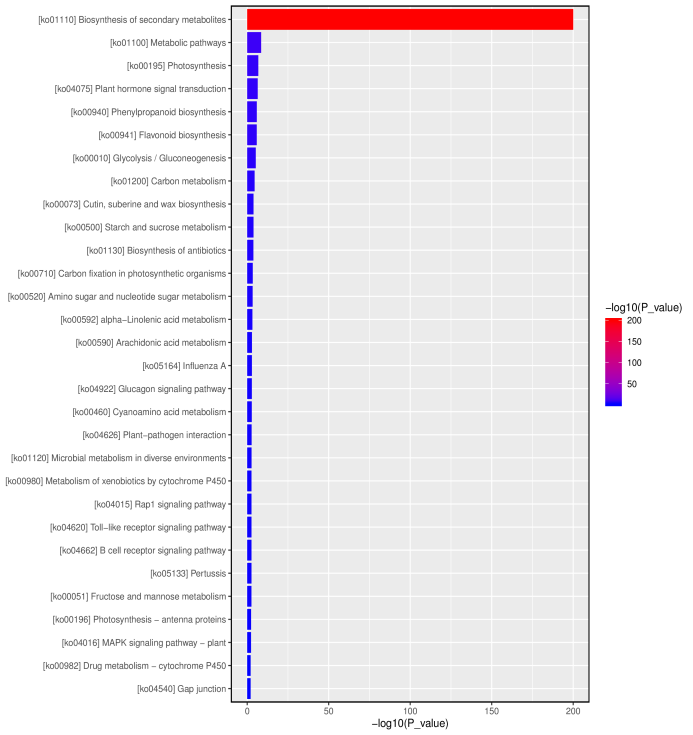
**

BrS1 vs BrS0 ONS1 vs ONS0

**
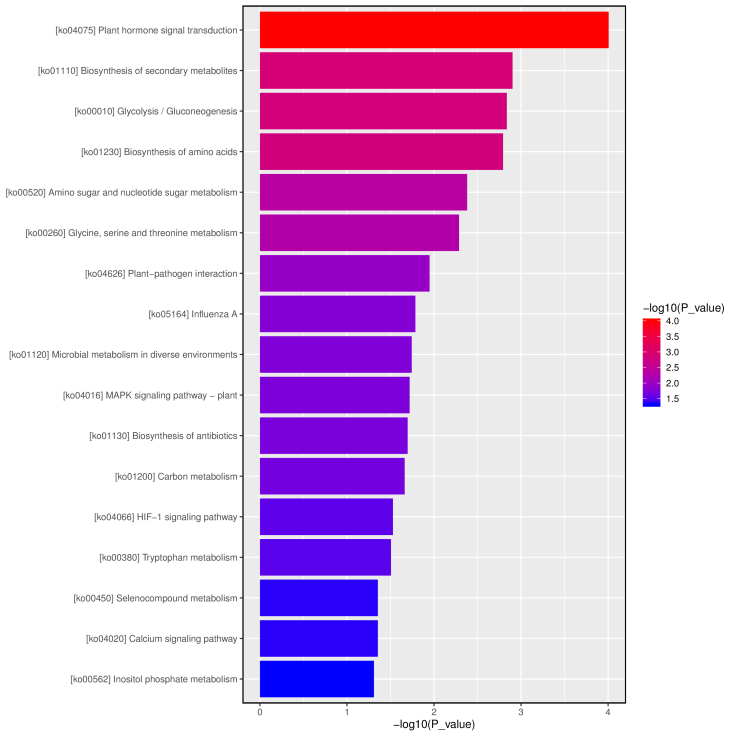

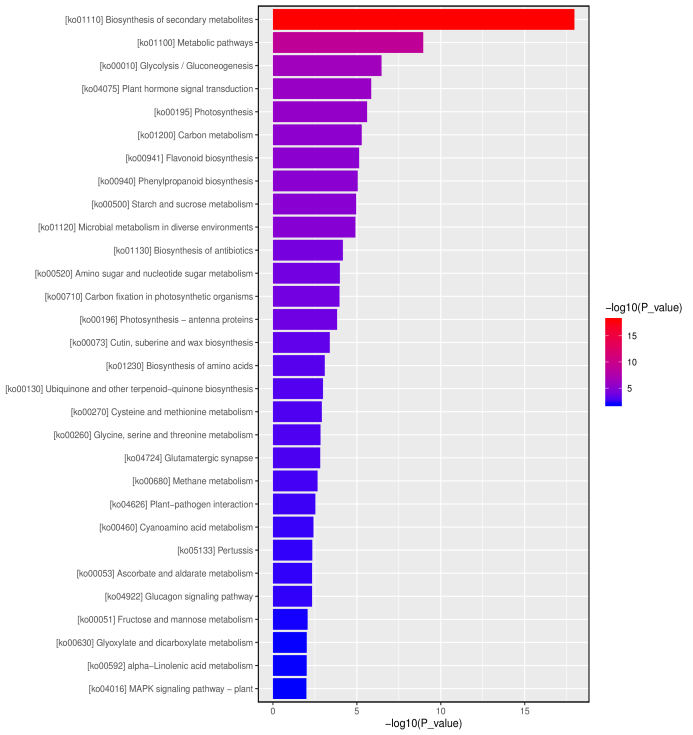
**

BrS2 vs BrS0 ONS2 vs ONS0

**Fig. S7.** GO function classification of the DEGs involved in biological processes during early *V. corymbosum* 'O'Neal' and 'Bluerain' fruit development.


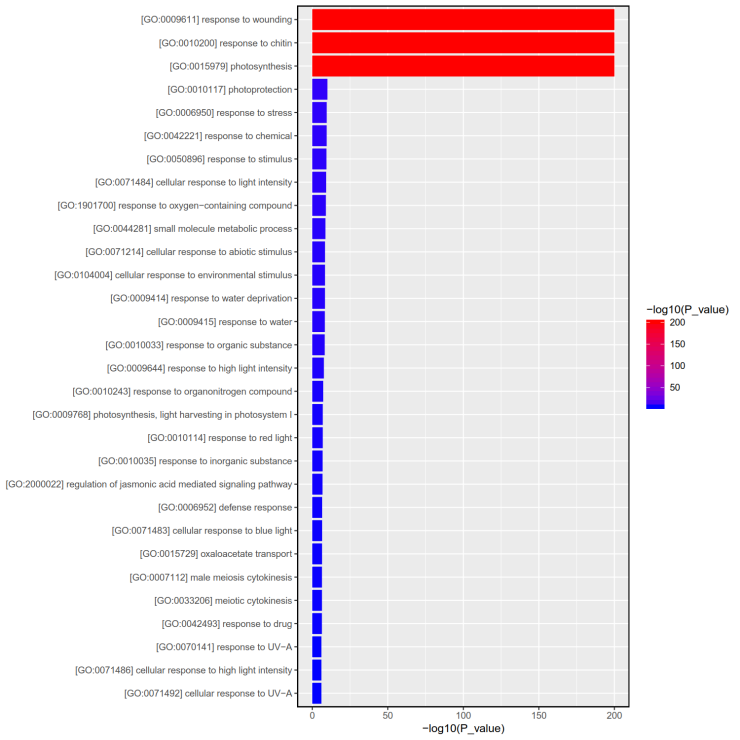


BrS0 vs ONS0


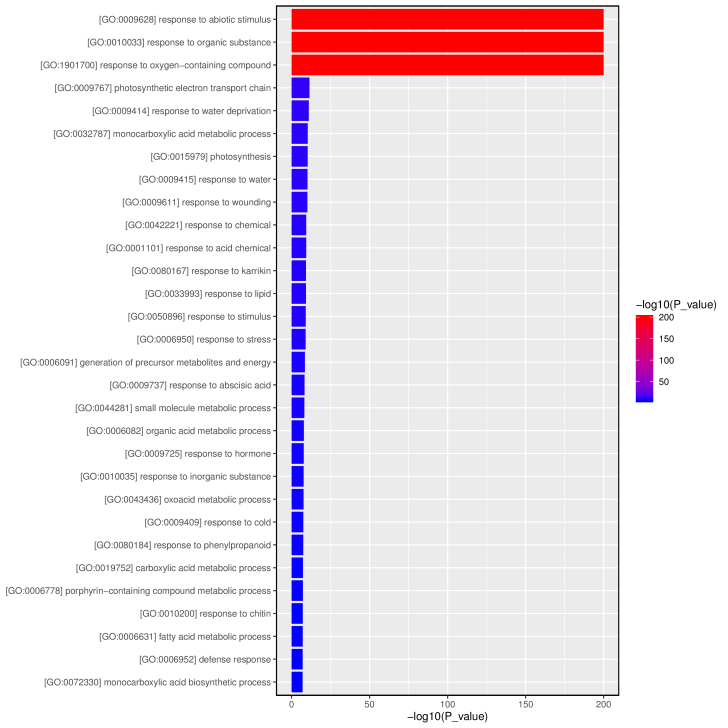

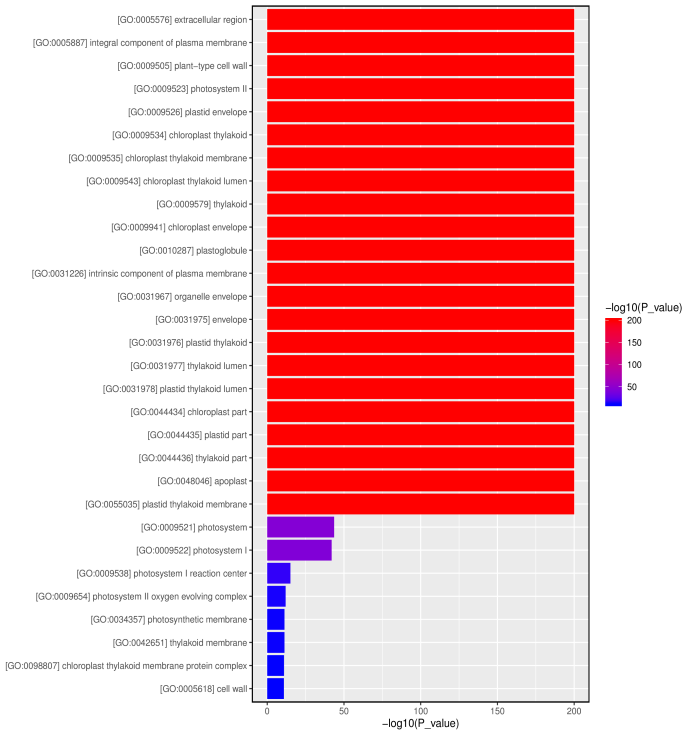


BrS1 vs BrS0 ONS1 vs ONS0


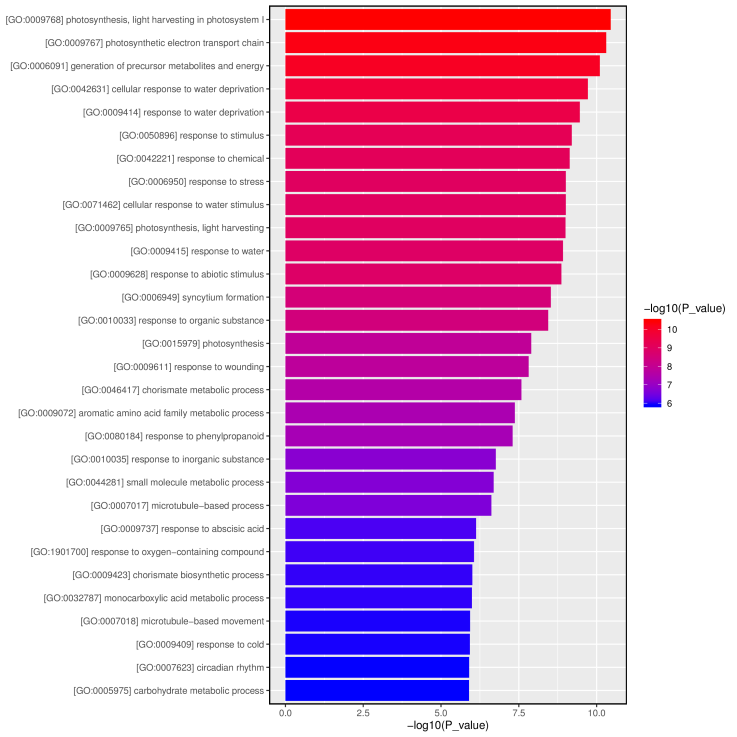

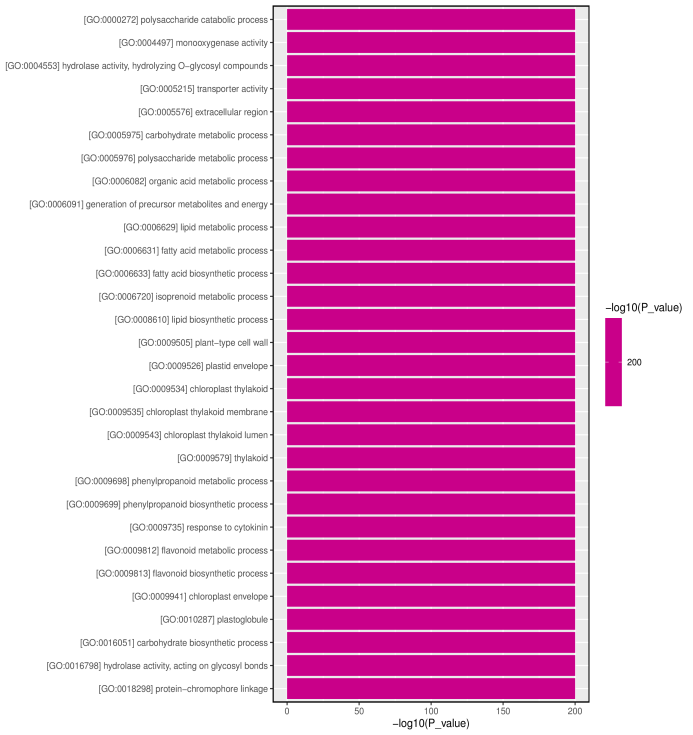


BrS2 vs BrS0 ONS2 vs ONS0

1. Correspondence: [yangli@zjnu.cn](#mailto:yangli@zjnu.cn) ; [gwd@zjnu.cn](#mailto:gwd@zjnu.cn)

   **^†^** Li Yang and Liang-miao Liu contributed equally to this work

   ^1^College of Chemistry and Life Sciences, Zhejiang Normal University, Jinhua, Zhejiang 321004, P. R. China

   ^2^Zhejiang Provincial Key Laboratory of Biotechnology on Specialty Economic Plants, Zhejiang Normal University, Jinhua, Zhejiang 321004, P. R. China

   ^3^Zhejiang College of Security Technology, Wenzhou, Zhejiang 325000, P. R. China [↑](#footnote-ref-2)
